# Exposure to worrisome topics can increase cognitive performance when incentivized by a performance goal

**DOI:** 10.1038/s41598-023-50036-0

**Published:** 2024-01-12

**Authors:** Timothée Demont, Daniela Horta-Sáenz, Eva Raiber

**Affiliations:** Aix Marseille University, CNRS, AMSE, Marseille, 5-9 Boulevard Maurice Bourdet, 13001 Marseille, France

**Keywords:** Human behaviour, Psychology and behaviour

## Abstract

Worrisome topics, such as climate change, economic crises, or pandemics including Covid-19, are increasingly present and pervasive due to digital media and social networks. Do worries triggered by such topics affect the cognitive capacities of young adults? In an online experiment during the Covid-19 pandemic (N=1503), we test how the cognitive performance of university students responds when exposed to topics discussing (i) current adverse mental health consequences of social restrictions or (ii) future labor market hardships linked to the economic contraction. Moreover, we study how such a response is affected by a performance goal. We find that the labor market topic increases cognitive performance when it is motivated by a goal, consistent with a ‘tunneling effect’ of scarcity or a positive stress effect. However, we show that the positive reaction is mainly concentrated among students with larger financial and social resources, pointing to an inequality-widening mechanism. Conversely, we find limited support for a negative stress effect or a ‘cognitive load effect’ of scarcity, as the mental health topic has a negative but insignificant average effect on cognitive performance. Yet, there is a negative response among psychologically vulnerable individuals when the payout is not conditioned on reaching a goal.

## Introduction

Distressing topics regarding current problems or future uncertainties are increasingly pervasive and salient. Young people in particular are constantly confronted with worrisome issues through digital media and social networks. Current examples include the Covid-19 pandemic, economic slowdown, and climate change. Recent studies have highlighted the difficulty of resisting the impulse to consume negative news, especially during periods of crisis, with negative health consequences^1,2^. At the same time, the rate of anxiety in the population has been increasing, reaching very high levels in many contexts, including France, and affecting particularly young adults^3–5^.

This paper studies the impact of exposure to such worrisome topics on cognitive performance. Worries can be distracting and make it hard to concentrate on the task at hand. The behavioral economics literature has shown that stressful events and economic hardships may impair cognitive performance and economic decisions^6–8^. The psychology of poverty and scarcity theory suggests that anxiety, linked to financial vulnerability or uncertainty regarding future resources, can increase ‘cognitive load’, thus imposing a tax on ‘mental bandwidth’^9–13^. For instance, making financially-constrained people think about a worrisome financial decision has been found to decrease cognitive performance^14^. Financially-constraint workers can be more productive after receiving their cash payments, which alleviates their financial worries^15,16^. Students from a lower socio-economic background were found to score worse on mathematical exam questions that make large sums of money salient, suggesting that financial salience can capture the attention of those financially vulnerable^17^. In a lab experiment, asset losses have been found to decrease cognitive performance by decreasing accuracy and increasing response times^18^. Closer to this paper, a recent study found that people affected by negative Covid-19 shocks performed worse in a cognitive reflection task. Yet, reminding participants of negative emotions did not affect their cognitive performance^19^.

However, the psychological literature has highlighted the possibility of an opposite effect, called ‘tunneling’^20,21^. Given the high cost of bad decisions when resources are limited, a scarcity mindset can cause an attentional focus on the problem at hand. Studies have shown that this effect can increase the efficiency of resource use, memory-encoding, and rationality^9,22,23^. Yet, other studies find no effect of financial scarcity on cognitive function and decision-making^24–26^. These mixed results led some researchers to argue recently that economic rationality might be unaffected by temporary impairments in cognitive resources^27,28^. A recent review of scarcity theory finds some support for both tunneling and cognitive load mechanisms in the literature, though it concludes that important methodological issues prevent firm conclusions^21^.

A similar story can hold when considering the effect of stress on cognitive outcomes. Exposure to worries can increase stress levels, which have been shown to increase cognitive performance until a certain point and decrease performance thereafter (the so-called ‘Yerkes-Dodson law’)^29^. Yet, later studies failed to find empirical support for such an inverted-U relationship between stress and performance^30–33^. Moreover, how stressful a topic is perceived can depend on its contents but also on the profile of the person being exposed.

As a result, different effects can be expected based on the type and the consequences of the worries being considered. While certain worries might alter cognitive functions and decrease performance, others might be motivating, in particular when they relate to future hardships that can still be mitigated through effort. People with the time and means to cope with the consequences might even see those issues as a challenge^34–36^. Yet, such capacity might be available only to individuals with certain favorable characteristics, such as good mental health or financial stability.

Furthermore, the overall response to worries and the relative strength of these opposite effects are also likely to depend on what is at stake. For instance, if there is a clear performance goal to strive for, worries may push individuals to focus on the goal and exert greater effort in scarcity-related tasks. Various laboratory studies have shown that the financial incentive structure can affect effort and task performance^37^. Yet, the literature on the effect of stake size and the amount of bonus payments on performance is also mixed^38–40^.

Despite the potentially important implications of the different effects discussed, there is limited causal evidence regarding their existence, relative strength, and underlying mechanisms – especially in settings that involve real-life worries and stakes^21^. This paper aims to fill the gap by generating meaningful exogenous variations in both the type of worry and emphasis on a performance goal.

## Study setting

In an online experiment conducted during the Covid-19 pandemic, we investigate the impact of different types of worries on the cognitive performance of university students. We leverage real-life sources of anxiety made salient by the pandemic: mental health issues related to social restrictions and future labor market uncertainties linked to the economic contraction. The treatments are motivated by the evidence that, during the pandemic, young people worried mostly about uncertain employment opportunities and that social restrictions burden their psychological well-being^41–46^. Moreover, the different topics of the two treatments allow us to discuss different mechanisms.

**Figure 1:**
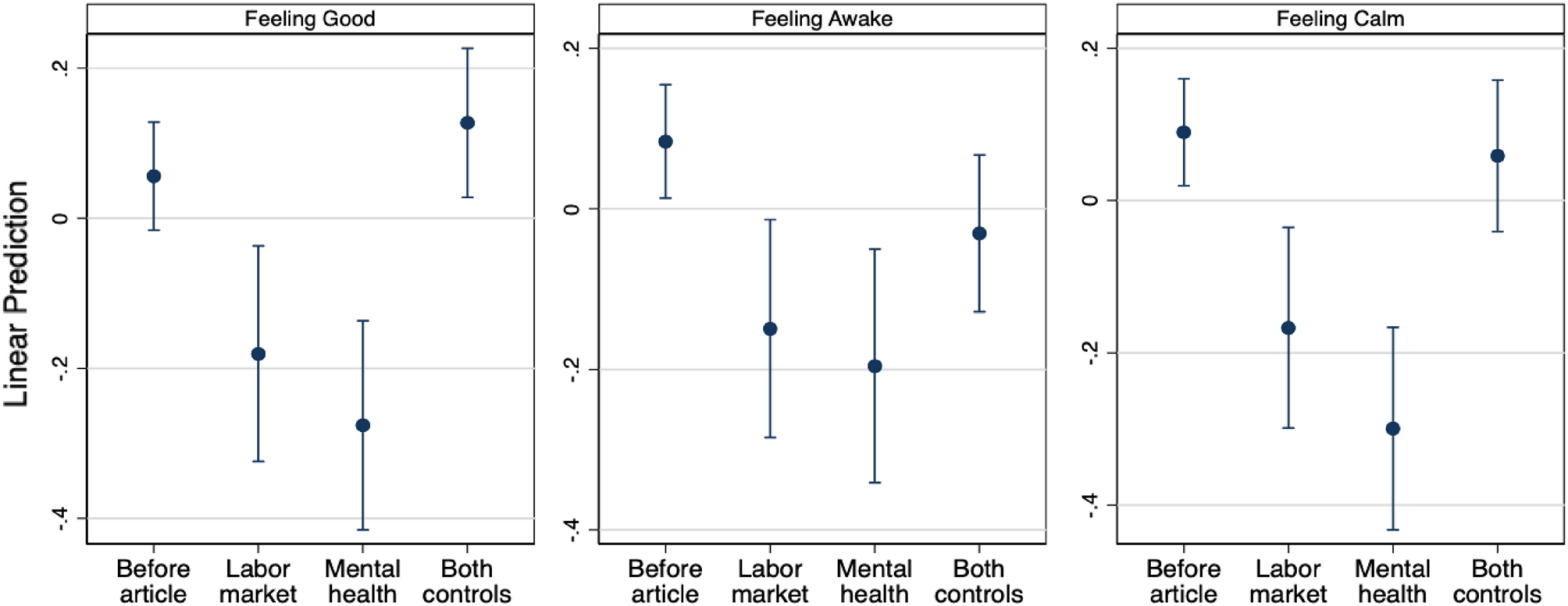
Emotional states before and after the topic treatments. Note: Linear prediction of emotional state before and after the topic treatment, with 95% confidence intervals. The standardized scores are based on four questions for each mood (two positively phrased, two negatively). Includes pre-registered baseline controls: gender, field of study, undergraduate, scholarship recipient, as well as age and number of correct matrices in the first round. See SI Table 1.

In addition, we cross-randomized the way performance is rewarded. Participants were either compensated for each correct answer or received payment upon reaching a specified threshold, set as correctly solving half of the tasks. This goal was chosen to be achievable for most participants (based on pilot data) and mirrored exam conditions where half of the points are needed to pass. This reflects many real-life situations, such as passing an academic, qualifying, or entrance exam or reaching a performance threshold to qualify for a bonus, job, or promotion. We thus have a between-subject design where individuals were randomly exposed to one topic - either a treatment topic or a control/placebo topic - and do the performance task under one payment scheme. Supplementary Information (SI) Figure 1 illustrates the experimental design and SI Figure 2 the survey design.

Our main outcome is cognitive performance – the ability to solve challenging problems, by processing information quickly and going beyond memorization or imitation (also sometimes referred to as cognitive ability, abstract reasoning, or fluid intelligence)^47,48^. It is a strong predictor of key real outcomes, such as educational and professional success or good health^49–52^. Beyond average treatment effects, we study carefully the heterogeneity of such effects, focusing particularly on characteristics linked to financial or mental health vulnerability that can decrease the ability to cope with negative facts.

In practice, we ran the experiment on a sample of 1503 students at a French public university with a mixed student body, between February and April 2021. The treatment and control topics included a newspaper article and two graphics, followed by comprehension and reflection questions. The mental health (MH) topic discussed the situation of students during a lockdown. At the time, France was just out of its second national lockdown, partial lockdowns were progressively put in place at the regional level, and vaccines were not yet available for the general population. The topic thus increased the salience of a current stressful issue which participants had been unwillingly confronted with. The labor market (LM) topic discussed the projected difficult labor market situation of young adults. With students not being in the labor market yet, the topic could stimulate anxiety about an uncertain future. Yet, by nature, the topic leaves room for action, as students are potentially able to influence their personal outcomes through increased effort over the coming months and years. The articles were included to give context and substance to the treatment but generally did not provide novel information: only 16% and 9% of participants stated that they “learned a lot” from the LM and the MH topics, respectively. Participants in the control group were presented with topics that were intentionally selected to be non-distressing. These topics included animal welfare and NASA’s space program, and they were designed to occupy participants for an equal amount of time and to induce a similar “reflective state” without causing any worry or stress.

## Results

### Treatement effect on emotions

We first assess the effectiveness of the treatment by measuring participants’ emotional responses. We administer a multidimensional mood questionnaire^53^, randomly directly before or after the treatment topics. Figure 1 illustrates the responses for the three mood dimensions measured with the questionnaire. The regression results are displayed in SI Table 1. We control for baseline characteristics in every specification (see SI section 2.1. for specification details). We focus on the results that pool both control topics, as the emotional states of participants after either of the control topics are nearly identical. SI section 2.2 discusses the differences between the two control topics.

We find that the two control topics do not affect the participants’ emotional state significantly in any dimension compared to those asked before, except for weakly making respondents more tired (0.115 Standard Deviation (SD), p=0.062) and feeling better (0.098 SD, p=0.098). By contrast, we find that participants state feeling significantly worse after facing either of the two treatment topics compared to the control topics (LM: -0.308 SD, p=0.001, MH: -0.403 SD, p=0.000). Participants are also weakly more tired after reading the mental health article (-0.165 SD, p=0.065). Importantly, they are significantly less calm after reading the two treatment articles compared to those reading the control articles (LM: -0.226 SD, p=0.007; MH: -0.358 SD, p=0.000). The effects of the two treatments are not significantly different from each other on any of the three mood dimensions. Overall, the treatments had a negative effect on the emotional state of the participants. Yet, we cannot exclude an experimenter demand effect as participants could feel that after the treatment topics they are expected to feel worse.

### Treatment effect on cognitive performance

We measure cognitive performance through a Raven-matrices-like task, in which participants have to find the missing element in an incomplete series of colorful and abstract forms^54^. Students were either faced with a standard payment for each correct answer (piece-rate payment) or received this same payment only upon reaching an achievable minimum level (threshold payment).

**Figure 2:**
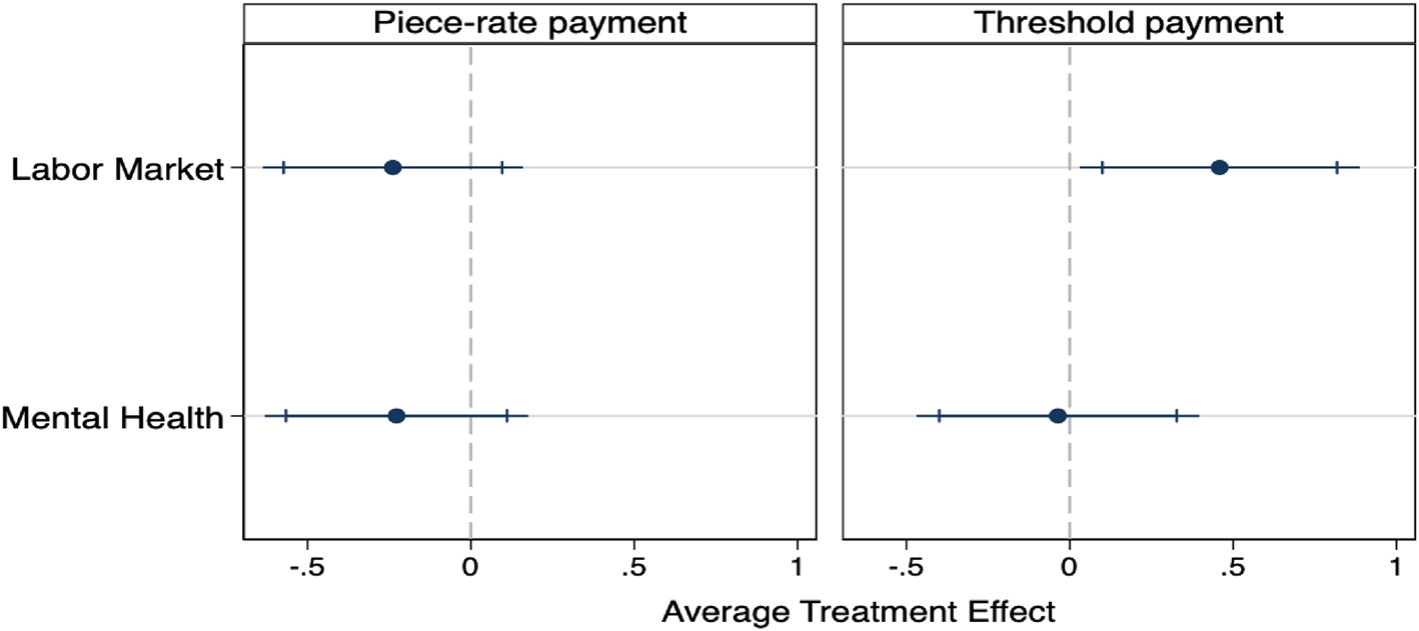
Treatment effect on cognitive performance. Note: Treatment effects on cognitive performance according to the payment scheme, with 90% and 95% confidence intervals. The dependent variable is the number of correct matrices (Minimum possible: 0, maximum possible: 10). Includes pre-registered baseline controls: gender, the field of study, undergraduate, scholarship recipient, as well as age and number of correct matrices in the first round. See SI Table 2.

Figure 2 illustrates the treatment effects on cognitive performance, measured through the number of correct matrices (out of 10) for each payment scheme. Under the piece-rate payment, the coefficients for both treatments are negative but not significantly different from zero. The coefficients (LM =-0.238 matrices, MH = -0.227 matrices, see SI Table 2) are smaller in absolute terms than 0.73 matrices – the minimal detectable effect pre-registered based on pilot data – which we consider a ‘medium-size effect’. However, based on a “two one-sided tests” procedure^55,56^, we cannot reject the presence of a ‘small negative effect’ defined as -0.37 matrices – i.e. half of the minimal detectable effect (see SI Section 2.3 for discussion). Furthermore, while the treatment effects are not significant when compared to the pooled control groups, they are negative and significant when compared to the space exploration control only (LM = -0.317 matrices, p=0.039; MH =-0.306 matrices, p=0.049; SI Table 19).

However, under the threshold payment, the LM treatment positively and significantly affects students’ cognitive performance (+0.459 matrices, p=0.036). Treated students improved their performance by 7% relative to the control group mean. In contrast, the MH treatment does not lead to any significant effect. Interestingly, the payment scheme alone does not have a significant effect on cognitive performance (SI Table 4). It is the combination of the threshold payment and LM treatment that enhances the cognitive performance of students.

Moreover, we find that the effect is larger (+0.584 matrices, an increase of 9%, p=0.009, SI Table 5) when we include only individuals who answered correctly at least three of the four comprehension questions (88% of the participants). This suggests that the effect is indeed driven by those who were attentive to the articles and questions. Yet, students might have given wrong answers for different reasons in the different treatment groups limiting the interpretation of these results.

The positive effect of the LM treatment under the threshold payment scheme holds when compared to each control group separately (see SI section 2.2). The coefficient is still significant at 10% when controlling for the testing of two hypotheses (p=0.062, SI Table 3), corresponding to within-payment scheme multiple hypothesis testing (MHT). The p-value drops to 0.133 when controlling for the testing of four hypotheses (between-and-within-payment scheme MHT).

### Investigating heterogeneity in the treatment effects

**Figure 3:**
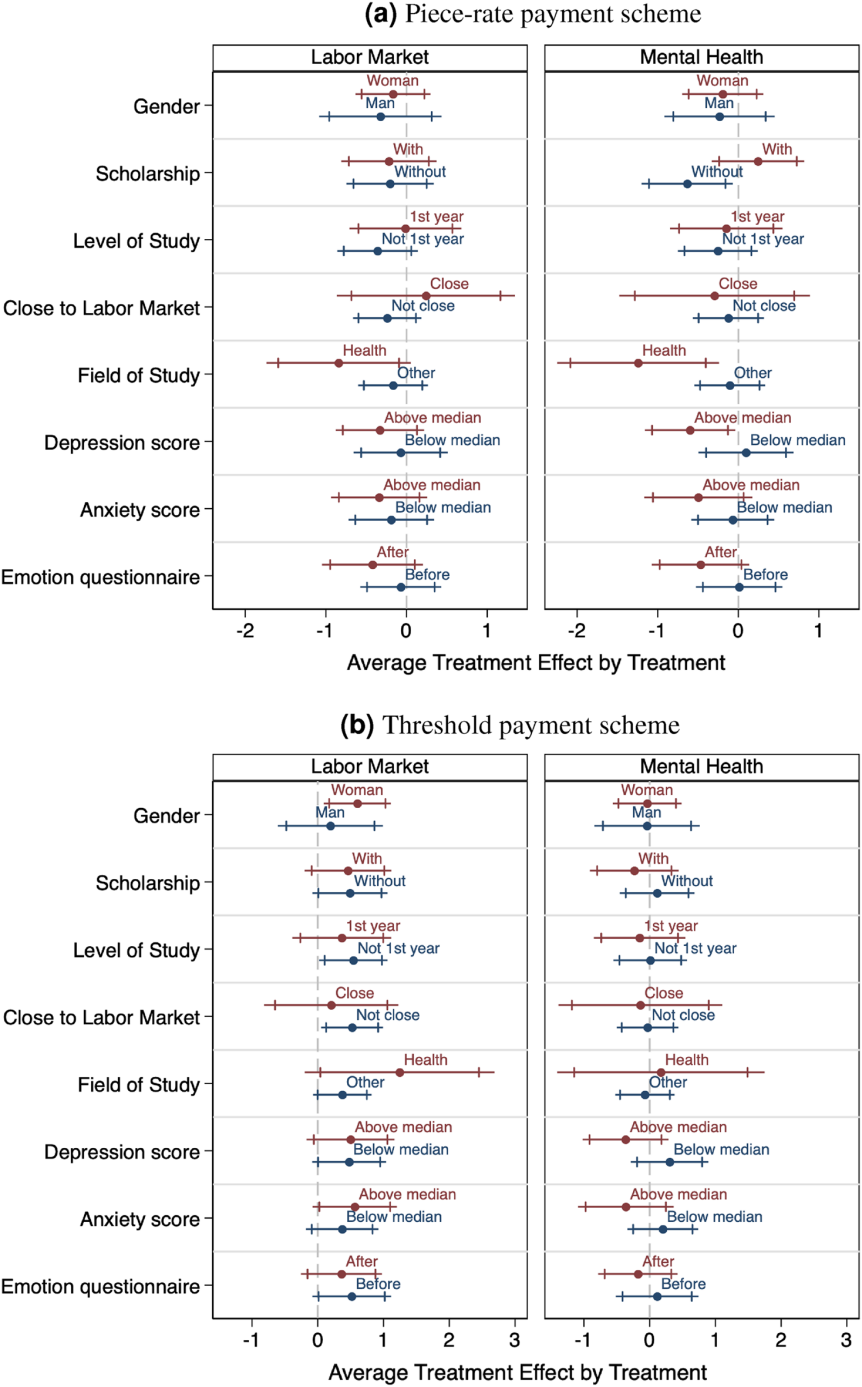
Treatment effect on cognitive performance: pre-registered heterogeneity. Note: Differential treatment effects on cognitive performance for the piece-rate payment (panel a) and the threshold payment (panel b) for the pre-registered groups, with 90% and 95% confidence intervals. The dependent variable is the number of correct matrices (Minimum possible: 0, maximum possible: 10). Includes pre-registered baseline controls: gender, the field of study, undergraduate, scholarship recipient, as well as age and number of correct matrices in the first round. “Emotion questionnaire” refers to the emotions questionnaire being asked before or after the topic treatment. See SI Tables 6 and 7.

We pre-registered heterogeneity for gender, receiving a state-funded scholarship as a measure of parental income, the field of study, the level of study, being close to finishing their studies, depression and anxiety score, and whether the mood questionnaire was asked before or after the treatment topics. Figure 3 Panel A illustrates the treatment effect for the different subgroups for the piece-rate treatment and Panel B for the threshold payment (see SI Tables 6 and 7 for the regression results).

Under piece-rate payment, we find no significant treatment effect of the LM topic in any pre-registered dimension. For the MH topic, we find that it decreases cognitive performance among those without a scholarship (-0.634 matrices, a decrease of 9%, p=0.028), those with a depression score above the median (-0.600 matrices, a decrease of 9%, p=0.036), and among students in “health science” (-1.244 matrices, a decrease of 17%, p=0.016) - though the sample of health students is too small to draw clear conclusions.

We find that the MH topic negatively affects the depression score though the questions were asked about the previous weeks (see SI Table 13 column (1)). We verify if assignment to treatment changes the group composition into those below and above the median. If treatment changed the composition, the subgroups would not be comparable between treatments. However, we find that the median for each treatment cell is the same, such that using the overall median to divide the participants into two groups and the group-specific median leads to the same results. Results are also the same if we correct the mental health score by the treatment effect (see SI Table 8). We also find in a quantile regression that the MH topic negatively affects those with an already high score (see SI Table 9).

We expected those with a scholarship to be more vulnerable as they come from a poorer background. Yet, receiving a state scholarship might also give students a stable income and thus improve their financial stability compared to those who do not receive it but have a similar financial background. The effect on those with poor mental health is in line with expectations: those who are especially vulnerable perform worse. The coefficient for the anxiety score goes in the same direction, though is not significant (-0.496 matrices, p=0.146).

Under the threshold payment, the treatment effect of the LM topic is consistently positive for all subgroups. The treatment effect appears to be especially strong for women (+0.601 matrices, an increase of 9%, p=0.021), those not in the first year of their studies (+0.540, an increase of 8%, p=0.042), and those not close to graduation and labor market entry (+0.521, an increase of 8%, p=0.030) - though none of the coefficients is significantly different from each other. The MH topic does not have a significant effect on cognitive performance for any pre-registered subgroup.

### Causal machine learning

 We use a “Causal Forest” to uncover subgroups that react differently to our treatments in a data-driven approach^57^. This heterogeneity analysis allows us to go beyond the pre-defined subgroup analysis by accounting for high dimensional combinations of covariates. We estimate the Conditional Average Treatment Effect (CATE) on a vector of observable characteristics, including baseline controls and a large number of covariates that provide information on participants’ financial situation, expectations, family background, mental health measures, Covid-19 experience, and some self-perception questions. We then use the predicted CATE to rank the observations from those with the lowest CATE to the highest CATE and group them into quartiles.

We apply the causal forest to each of our treatments. However, after assessing the quality of the forest’s estimates, we only detect heterogeneity in the LM treatment and the threshold payment — the only treatment where we find an average treatment effect (see SI Section 2.4 for further explanation). Therefore, we limit our analysis to this treatment arm. To compare the two most contrasting groups, we analyze the difference between those in the first and the fourth quartiles (see SI Tables 24 and 25). For the first quartile, the average treatment effect is close to zero and not significant (-0.35 matrices, p=0.508) while for the fourth quartile, the average treatment effect is strongly positive and significant (+1.19 matrices, an increase of 18%, p=0.02). We compare the characteristics of those within these groups to uncover what predicts whether or not respondents benefit from the treatment (focusing on those with a difference significant at 1%). Table 1 displays the results.

**Table 1:** Causal Forest: cognitive performance - labor market and threshold treatment.

Variable	Highest quartile	Lowest quartile	Diff.	P-values
	*Panel A. Baseline controls*			
Age	20.346	22.928	$-$2.582	0.00***
Woman	0.713	0.703	0.010	0.85
Scholarship	0.346	0.543	$-$0.198	0.00***
1st year student	0.404	0.261	0.144	0.01**
Close to labor market	0.066	0.196	$-$0.129	0.00***
Fatigued	0.779	0.804	$-$0.025	0.61
First round matrices	1.875	1.993	$-$0.118	0.45
	*Panel B. Field of study*			
Health Sciences	0.088	0.101	$-$0.013	0.71
Arts and Languages	0.162	0.094	0.068	0.10*
Law, Economics, Management	0.250	0.254	$-$0.004	0.95
Science and Technology	0.257	0.370	$-$0.112	0.05**
Humanities and Social Science	0.235	0.181	0.054	0.27
	*Panel C. Financial Situation*			
Having financial struggles	0.118	0.341	$-$0.223	0.00***
Can afford extra expenses	0.875	0.717	0.158	0.00***
Having own salary	0.118	0.203	$-$0.085	0.05*
	*Panel D. Expectations*			
Low prob. success career	0.140	0.210	$-$0.070	0.13
Low prob. success studies	0.449	0.420	0.028	0.64
Pessimistic about the next 5 years	0.324	0.377	$-$0.053	0.36
Pressure to have diploma	0.434	0.297	0.137	0.02**
Pressure to have good grades	0.250	0.217	0.033	0.53
	*Panel E. Family Background*			
Migrant	0.029	0.304	$-$0.275	0.00***
Living alone	0.250	0.290	$-$0.040	0.46
Father university degree	0.551	0.174	0.378	0.00***
Mother university degree	0.816	0.094	0.722	0.00***
Both parents work	0.787	0.406	0.381	0.00***
	*Panel F. Mental Health*			
Depression	0.441	0.543	$-$0.102	0.09*
Anxiety	0.397	0.471	$-$0.074	0.22
	*Panel G. Covid-19 Experience*			
Had Covid-19	0.103	0.036	0.067	0.03**
Family member had Covid-19	0.272	0.304	$-$0.032	0.56
Personal traumatic Covid experience	0.184	0.268	$-$0.084	0.10*
Family member lost job	0.176	0.225	$-$0.048	0.32
Positive attitude tw vaccination	0.449	0.348	0.101	0.09*
Lock-down alone	0.029	0.109	$-$0.079	0.01***
Seeing friends	2.985	0.949	2.036	0.00***
Going to the university	1.463	0.935	0.528	0.01***
	*Panel I. Self- perception*			
Cognitive rigidity	3.110	3.761	$-$0.651	0.00***
Cognitive control	2.867	2.993	$-$0.126	0.27
Locus of control	17.945	20.007	$-$2.062	0.00***

Note: * $p< 0.1$, ** $p < 0.05$, *** $p < 0.01$. Compares the characteristics of students who belong to the highest and lowest quartiles of the CATE distribution. Those in the highest quartile are predicted to have a positive treatment effect while those in the lowest quartile are predicted to have no treatment effect.

Similar to the pre-registered heterogeneity analysis, we find that students without a scholarship and students not close to graduation and labor market entry are more likely to be in the first quartile than in the last, thus benefiting from being treated with the LM topic and the threshold payment (Table 1, Panel A). Furthermore, we can pin down other characteristics that seem relevant to generating a positive effect. First, the financial situation and family background plays an important role: on average, participants in the highest quartile are less likely to struggle financially, more likely to be able to cover additional expenses, and less likely to work beside their studies (Panel C). They are more likely to be non-migrants, have highly educated parents, and have both their parents working (Panel E). Second, participants who were more socially active during the lockdown are more likely to respond positively to the treatment: students in the highest quartile state not having passed the lockdown alone and claim seeing friends and going to the university more often than those in the lowest quartile (Panel H). Finally, being able to switch from task to task easily (lower cognitive rigidity) and having a lower locus of control seem related to performing better (Panel I).

### Effect on other outcomes

For the other outcomes (see Methods section), we do not have variation in the payment scheme. Hence, we only study the effect of being exposed to the different topics. We do not find any significant effect on cognitive reasoning or risk-taking (SI Table 10 columns 1-4). We also verify if the treatment topics affect the willingness to pay for a lottery ticket to win a real individual online coaching session by a leading student counselling firm (SI Table 10 columns 5-6). We do not find any significant effect (though the coefficients are positive as expected) when controlling for baseline characteristics. With extended controls, we find a weak positive effect of the LM treatment (significant at 10%). Investigating further, the LM treatment significantly increases the likelihood of respondents choosing the module “interview simulation” as one of their two module choices by almost 20%. We find no other significant effect for either of the two treatments (SI Table 11).

## Discussion

### Different effects of the two topics and interaction with the goal-based payment scheme

The LM topic is a reminder for students that finding a job at the end of their studies might not be easy. Yet, it is not an immediate problem and it is partly endogenous, as students can take action to face up to the adverse situation, in particular through academic effort. Hence, the topic can be motivating – at least for those who believe that they have the possibility to cope with the consequences. This interpretation is underlined by the observation that the positive result is driven by those not too close to labor market entry. We also find that the LM treatment significantly increases the perceived importance of finding a well-paid job after university (see SI Table 12 column 2), as well as the willingness to pay for a real career coaching session. The LM treatment thus increased the salience of job-finding challenges at the end of the studies. Psychological studies have shown that a higher stress level can increase performance when the stressor is seen as a challenge rather than a threat^35,58^. For this, the stressor might need to be ‘controllable’^36^. This can explain why we see a positive effect for the LM topic that leaves ‘scope for action’.

This motivation effect seems to have been picked up by the more challenging or motivating threshold-payment scheme. Payment schemes that provide explicit and achievable goals can enhance motivation and performance more than schemes where payment is linked to the individual unit of output^59^. Indeed, the threshold in our experiment is achievable: 76% of the participants reach the minimum level of 5 correct answers. The threshold payment should be especially motivating for those who believe they are just below the threshold. We indeed find that the treatment effect is the strongest among students with a lower “baseline cognitive performance score” (see SI Figure 3). The score measures how many matrices they got right in the incentivized training task before the treatment. This result suggests that the threshold-payment scheme successfully motivated those students to increase effort.

We also find that the threshold payment increases the perceived importance of having good grades as well as a well-paying career (see SI Table 12 columns 1 and 2). The threshold condition seemed to make academic performance more salient and connect it with the cognitive performance task. The results are thus consistent with a ‘tunneling effect’ where the attention of the respondent focuses on a task that is related to the source of scarcity or worry^9,21^. It is likely that the combination of the specific topic and the goal-based payment created the conditions to improve students’ cognitive performance.

Contrary to the LM topic, the MH topic exposed students to a current and certain ‘state of affairs’, with very limited scope for action, and not linked with academic performance. This decreases to likelihood of observing a ‘tunneling effect’ since the cognitive performance task is unrelated to the topic. The topic thus is only expected to increase stress levels and to tax ‘mental bandwidth’. We indeed find that the MH topic worsens participants’ depression score (SI Table 13 column 1) as well as locus of control – their belief that their outcomes are mainly driven by their actions rather than chance and circumstances (SI Table 13 column 2). In the piece-rate treatment, we find a negative effect among different subgroups, including those with a high depression score. Thus, there is a potential detrimental effect of this topic on the most vulnerable. The negative effect, albeit weak, is in line with the ‘mental bandwidth’ effect or a negative stress effect, caused by the different content (mental health vs. job uncertainty) or the deterministic nature of the issue.

Under the threshold payment, we see an average null effect of the MH topic, and no sub-group with a positive or negative effect. The threshold payment is only expected to be motivating if people believe that they will reach the goal and effort is useful. The MH topic might just do the opposite. Moreover, if the treatment topic taxes the ‘mental bandwidth’ and thus increases the cost of effort, people who are not sure to pass the threshold might decrease their effort because the expected benefits are lower. This could counteract the otherwise motivating effect of the threshold payment.

### Inequality-widening mechanism

We find that the increase in performance, after the exposure to a worrisome topic with scope for action and given a goal-based payment scheme, is driven by participants from a better-off background. Students with larger socioeconomic resources seem to be able to draw motivation from future uncertainty, provided the right incentives are in place. This is not the case for those with a more vulnerable profile. Hence, during periods when students are faced with worrisome news, we expect those who face financial, social, or psychological vulnerabilities to perform worse than better-off students. We hypothesize that they are less able to perceive the opportunities in the situation. Therefore, they are more often blocked by worries about negative consequences. Such a mechanism implies that students with unequal preexisting socioeconomic characteristics will perform differently. The consequence is a deepening of preexisting inequalities. That is, our study highlights a new inequality-widening or poverty-preserving mechanism.

In the case of the Covid pandemic, this adds to the ample evidence that the negative consequences of lockdowns on student learning were especially severe among students from less-educated and poorer families^60–64^. More generally, our finding is in line with previous studies showing that financially vulnerable people experience a worse cognitive performance when faced with financially worrying tasks or situations^14,17^. It also echoes the so-called ‘broaden-and-build’ theory in psychology about the role of emotions^65^. Some experiments showed that positive emotions are linked to a broader scope of action or psychological resilience^66,67^. While our experiment induced negative emotions, it is likely that better-off participants benefited from having a more positive baseline emotional level. Indeed, we observe that facing financial struggles is associated with lower emotional scores along the three dimensions, among those who answered the emotions questionnaire before the treatment (see SI Table 14).

### Effect sizes and external validity

When students were invited to sign up and when they started the survey, they were informed about it including pandemic-related questions. While the invitation was sent to all students and, based on limited administrative data available, our sample looks similar to the general French student population, those who participated might differ in unobserved characteristics. Specifically, those who were the most vulnerable might have not taken part in the survey. Furthermore, we selected topics that were negative but correct and not sensationalist. On social media, people are often confronted with much more negative framing. Also, we only test the effect of one reminder of a topic that they probably have already heard a lot about and contemplated on several occasions. Therefore, we most likely find a lower bound of the potential negative effect.

In this online experiment, respondents were faced with worrisome topics in an online format – which mirrors how young adults generally consume news and are confronted with such topics. However, their cognitive performance response could have been different if the task was in person rather than online. Within a lab or face-to-face setting, participants might have already been under more stress. Again, this points towards us finding a lower bound of the potential negative effect. Yet, many professional tasks are nowadays done in a setting similar to the one in our experiment (online and distant clients). We thus argue that the online setting might be more relevant than a lab setting for this type of question. It also allowed us the run the experiment at a larger scale and recruit students who would not have participated in a lab experiment, which was important for the study of heterogeneity.

The question arises if these results hold beyond a sample of university students. First, students might be more responsive to our goal-based payment scheme, which resembles an academic exam situation. Yet, other papers have shown that similar payment schemes can affect the performance of adults^37,68^. Second, for students, the LM topic implies future difficulties, for which they can prepare individually through effort. Arguably, those already at work might see the topic more often as a threat and less often as a challenge. Yet, the key distinction between worries about topics with and without scope for action is general. For instance, a topic such as climate change can be seen as a challenge by some - who then get motivated to become active - and as a distracting worry by others.

## Methods

### Recruitment

Participants were recruited from the Aix-Marseille University (AMU), a large public French university. Interested students were invited by email to sign up for a paid online survey, approved by the AMU ethics committee. Students signed up with their unique official university email addresses. Between February and April 2021, for six weeks, 500 students who had signed up were randomly selected and sent an individual survey link on a Tuesday that was valid until the Friday of the same week. Participants had 90 minutes to finish the survey once started. Participants received payment for completing the survey of 7€ paid in the form of a voucher. The final amount received depended on the outcome of the different tasks and could vary between 3 and 28€, with an average payment of 16€. Out of the 500 students invited each week, on average, 52% started it. For the last week of invites, all those previously not selected received the survey link, as well as those who had been invited but had not started the survey.

### Ethics

All methods were carried out in accordance with relevant guidelines and regulations. The experimental procedure (recruitment process, consent form, treatments, and questionnaire) received approval from the ethics committee of AMU, reference number 2020-12-03-00. Informed consent was obtained from all participants at the beginning of the experiment.

In the experiment, students were purposely faced with reflection topics that can be expected to trigger negative emotions. To minimize the risk of an effect that extends beyond the duration of the experiment, the following steps were undertaken. First, students were informed that the survey would deal with the pandemic when they signed up and when they started the survey. Students could end the anonymous survey at any moment. Second, the informational material in the reflection topics (article, graphics), though negatively framed, were taken from standard newspapers and official organisations and judged “non-sensational”. They thus reflect information in a format that young people are constantly confronted with, presumably multiple times a day. The reflection questions were questions that young people are generally faced with as well. Third, at the end of the survey, participants were provided with additional information about the university’s support system and other relevant Covid information if they were interested. Participants who did not complete the survey after reaching the topic treatment stage were sent an email to inform them about the university’s support system.

### Survey structure

The survey, illustrated in SI Figure 2, started with an information and consent page which described the survey structure and the topics covered. The students were told that the survey would cover topics related to the pandemic. Following, respondents were asked some basic socio-demographic questions in the pre-questionnaire (age, scholarship recipient, field of study, gender). All participants then faced the first round of the cognitive performance task, incentivized by a linear payment scheme. Before the treatment articles, half of the sample randomly selected were asked questions about their current mood. The other half were asked the same questions after the treatment section. Participants were then moved on to the treatment topics.

After the treatment topics and the questions about their current mood, participants were faced with the second round of the cognitive performance task with the different payment structures. This was followed by incentivized measures for cognitive reasoning, risk-taking, and the willingness to pay for an individual online coaching program (see below).

Respondents were then asked questions about their studies, their career expectations and pressures, their Covid and lockdown experience, their current social habits, and their financial situation. This part also included questions for eliciting mental health, anxiety, and locus of control. The survey ended with a questionnaire on the socio-demographics of the student and their family. After the survey, participants were informed of their payment and could choose the method of payment (Amazon or Cultura voucher). Finally, they were provided information and links to the university’s and general support programs.

### Treatments

In the experiment, we cross-randomize the reflection topic and the payment scheme (see SI Figure 1). A translation of the treatment topics can be found in the SI section 3. The original French questionnaire can be found in section 5. 

#### Topic treatments

Participants were randomly shown one of four topics. Each topic contained an article of around 600 words including two graphical illustrations followed by non-incentivized comprehension questions. The topics also included several reflective questions to motivate the students to think about the topic and their situation. The format, length and number of questions were the same for all topics.

Both the Labor Market (LM) and Mental Health (MH) topic included information on the negative consequences of the Covid pandemic and the lockdowns. For the control groups, we chose two different topics: one article about the progressive elimination of cage rearing in France (Animal Welfare) and one article on the future of the Artemis program to land humans on the moon again (Space Program). The two control topics differed in some dimensions (potential emotional response, forward-looking perspective) but were both chosen to not make respondents anxious or worried about their own situation (see discussion in SI section 2.2).

All articles were taken from online platforms of actual newspapers and reflect information that students are confronted with daily. While addressing negative topics, we purposely chose articles that were factual and not sensational. The treatments were designed to make the labor market or mental health consequences of the Covid pandemic salient and to have participants reflect on their situation.

**Labor market (LM) topic**: The LM topic started with an article about the difficulty of young graduates entering the labor market. It mentioned a decreasing number of job offers due to the pandemic and described expected increases in unemployment. It included two graphs, one illustrating the expected increase in unemployment, and one highlighting the pessimistic view that many young people have about their labor market prospects. The reflective questions asked about the participant’s views on their labor market perspectives and their economic situation.

**Mental health (MH) topic**: The MH topic included an article about the psychological effects of the pandemic, focusing on the isolation of young people due to national lockdowns. It included a graph that illustrated the depression rate for different age groups and a graph displaying how prevalent mental health problems, stress and anxiety are. The reflective questions asked about the participants’ stress, and feelings of isolation and regret about their social life. Although France was not in lockdown at the time of the survey, there were still heavy restrictions in place, especially affecting students (e.g. remote or hybrid classes, a curfew, closed bars and cultural institutions).

#### Payment schemes

For the second round of cognitive performance, participants were randomly allocated to one of two payment structures, cross-randomized with the topic treatments. In the **piece-rate treatment**, participants received 1€ per correctly solved matrix. In the **threshold treatment**, participants received 1€ per correctly solved matrix only if they correctly solved at least 5 matrices. If they solved less than 5 matrices, their payout was 0€. If they solved 5 or more, their payout was the same as in the piece-rate treatment. The payoff structure was illustrated in a table.

### Main outcomes

**Cognitive performance:** To measure cognitive performance we use matrices from a collection of open-access abstract reasoning items (the matrix reasoning item bank^54^), similar to the Raven’s Matrices^69^. Participants were shown an incomplete matrix containing colorful, abstract forms with one missing field and were asked to select the missing item among six options.

In the first round - the training task -, participants were shown one example and then asked to correctly solve 4 matrices. They had a time limit of 3 minutes (45 seconds per matrix). For each correctly solved matrix, they received 0.5€. We use the number of correctly solved matrices of this first round as “baseline cognitive performance score”. In the second round, which took place after the treatment, participants were asked to correctly solve 10 items with a time limit of 6 minutes and 40 seconds (40 seconds per matrix). The payment scheme for the second round varied by treatment. The number of correctly solved matrices in the second round is our main outcome of cognitive performance.

**Emotional state:** Participants were asked a short translated version of the multidimensional mood questionnaire^53^, MDMQ to measure their current emotional state. This version of the MDMQ consists of 12 questions along three dimensions: feeling good versus bad, feeling awake versus tired, and feeling calm versus nervous. For each mood dimension, four questions are asked, two phrased positively and two negatively. Importantly, the MDMQ explicitly asks how the respondent feels at this current moment. Half of the participants were asked about their emotional state before the treatment and half after the treatment but before the incentivized tasks. This was cross-randomized with the topic treatments and the payment schemes to verify if administering the questionnaire in itself after the treatment topics had an effect.

### Other tasks and measures

We measure cognitive reasoning with three questions in the style of Frederick (2005)^70^. Participants had 4 minutes and 30 seconds to answer the questions and could earn 1€ per question.

To measure risk-taking, we used a lottery choice in the style of Gneezy and Potters (1997)^71^. Participants could invest up to 3€ from their baseline payment. They had a 50% chance to triple their investment, and a 50% chance to lose their investment.

Participants were offered to participate in a lottery to win an individual online coaching program of a market value of 385€. The coaching program included an orientation test and three individual sessions with a coach. Participants could choose between different modules: interview simulation, work methodology, self-confidence and stress management, and psychological support.

We compute a Depression score through the Patient Health Questionnaire-9^72^, PHQ-9. We changed the last question from the PHQ-9, which explicitly asked for the presence of suicidal thoughts, to one related to depression from HADS. We also compute an Anxiety score through a short version of the Hospital Anxiety and Depression Score^73^, HADS.

Finally, we use a short version of The Internal Locus of Control Index^74^, ICI. This index measures to what extent subjects feel they have control over their lives. Highly internal subjects feel responsible for the things that happen in their lives, while low internal subjects believe that factors beyond their control determine their lives.

### Descriptive statistics

Of the 1562 students that started the questionnaire, 1503 students finished it. As specified in the pre-analysis plan, we excluded respondents who took less than 8 minutes (20% of the median time) or more than 100 minutes to respond to the survey, as well as participants younger than 18 years and older than 30 years. The overall rate of attrition is 3.8%, with no differential attrition between the treatment groups. 779 participants played under the piece rate and 724 under the threshold payment scheme. 352 participants saw the labor market, 359 the mental health, 386 the animal welfare, and 406 the space topic. 66% of the respondents are female, the average age is 21.6 years. 70% are in their undergraduate studies, 27% in their masters or equivalent, and 3% are doing a PhD. 37% are from Science and Technology, 25% of the students are within the field of Law, Economics and Management, followed by Humanities and Social Sciences (17%), Art and Languages (14%) and Health Science (11%). Around 45% receive the means-tested state scholarship that depends on their parent’s income. The distribution of the covariates is balanced across the treatments on all pre-registered covariates (see SI Table 15). The joint orthogonality test is insignificant when comparing the topic treatments and the payment structures. As pre-registered, we include these variables as baseline controls in all our specifications.

### Statistical analysis

The main analysis was done by OLS in Stata 15. The displayed results are based on regression including the specified pre-registered controls with robust standard errors (see SI Section 2.1). The Causal Forest was run in R version 4.2.1 using the randomForest version 4.7-1.1 package. The regression tables are reproduced in SI Section 1. The Causal Forest method, as well as the related test, are described in SI Section 2.4. The experiment was pre-registered at https://aspredicted.org/h69ht.pdf. A discussion of the pre-registration plan can be found in SI Section 4.1, and the original pre-registration plan in SI Section 4.2.

### Supplementary Information

Supplementary Information.

## Data Availability

The dataset generated and analysed during the current study is available at Harvard Dataverse DOI https://doi.org/10.7910/DVN/VLQXJG. It includes the replication codes. The questionnaire is available in the Supplementary Information.
