# Supplementary material for: Exposure to worrisome topics can increase cognitive performance when incentivized by a performance goal
Source: Sci Rep. 2024 Jan 12;14:1204. doi: 10.1038/s41598-023-50036-0 (PMC10786830; doi:10.1038/s41598-023-50036-0)
Supplement: Supplementary file 1 — Supplementary Information. [file 41598_2023_50036_MOESM1_ESM.pdf]

Supplementary Information for:

Exposure to worrisome topics can increase cognitive performance when incentivized by a performance goal

Timothée Demont[1], Daniela Horta Sáenz,[1], and Eva Raiber[1,*]

[1]Aix Marseille University, CNRS, AMSE, Marseille, 5-9 Boulevard Maurice Bourdet, 13001 Marseille, France

[*]Correponding author: eva.raiber@univ-amu.fr

November 28, 2023

Contents

1 Main tables and figures **2**

1.1 Tables 2

1.2 Figures 12

2 Statistical methods and additional results **13**

2.1 Specification for treatment effects 13

2.2 Comparison of the control treatments 13

2.3 Equivalence testing and power calculations 19

2.4 Causal forest 21

3 Treatment topics (translated) **26**

3.1 Labor market 26

3.2 Mental health. 29

3.3 Animal welfare. 33

3.4 Space exploration. 37

4 Pre-registration **40**

4.1 Discussion 40

4.2 Original pre-registration plan 41

5 Questionnaire (original, in French, version with control treatment (space), emotional questionnaire before the treatment, and threshold payment) **44**

1 Main tables and figures

1.1 Tables

Table 1: Treatment effect on emotional states.

	Dependent variable:					
	Feeling good		Feeling awake		Feeling calm	
	(1)	(2)	(3)	(4)	(5)	(6)
Before Article	-0.0709	-0.0949*	0.115*	0.0778	0.0310	0.0130
	(0.257)	(0.098)	(0.062)	(0.123)	(0.618)	(0.815)
Labor Market	-0.308***	-0.318***	-0.119	-0.131*	-0.226***	-0.230***
	(0.001)	(0.000)	(0.164)	(0.069)	(0.007)	(0.004)
Mental Health	-0.403***	-0.406***	-0.165*	-0.168**	-0.358***	-0.362***
	(0.000)	(0.000)	(0.064)	(0.020)	(0.000)	(0.000)
Observations	1482	1482	1497	1497	1492	1492
Control Mean	15.82	15.82	11.96	11.96	12.69	12.69
LM - MH (p-val)	0.35	0.34	0.65	0.67	0.17	0.14
Baseline Controls	yes	yes	yes	yes	yes	yes
Extended controls	no	yes	no	yes	no	yes

Note: P-values in parentheses. Robust standard errors. * $p < 0.1$, ** $p < 0.05$, *** $p < 0.01$. The standardized scores are based on four questions for each mood (two positively phrased, two negatively). Baseline controls include dummy variables for gender, field of study, undergraduate, scholarship recipient, as well as continuous variables for age and number of correct matrices in the first round. Extended controls include dummy variables for the week, day, and time, fatigue, French mother tongue, French nationality, and having French parents.

Table 2: Treatment effect on cognitive performance.

	Dependent variable: Cognitive performance			
	Piece-rate payment		Threshold payment	
	(1)	(2)	(3)	(4)
Labor Market	-0.238	-0.242	0.459**	0.450**
	(0.240)	(0.238)	(0.036)	(0.043)
Mental Health	-0.227	-0.218	-0.0360	-0.0816
	(0.269)	(0.284)	(0.871)	(0.718)
Observations	779	779	724	724
Control Mean	6.97	6.97	6.64	6.64
LM - MH (p-val)	0.96	0.92	0.06	0.04
Baseline Controls	yes	yes	yes	yes
Extended controls	no	yes	no	yes

Note: P-values in parentheses. Robust standard errors. * $p < 0.1$, ** $p < 0.05$, *** $p < 0.01$. The dependent variable is the number of correct matrices. Minimum possible 0, maximum possible 10. Baseline controls include dummy variables for gender, field of study, undergraduate, scholarship recipient, as well as continuous variables for age and number of correct matrices in the first round. Extended controls include dummy variables for the week, day, and time, fatigue, French mother tongue, French nationality, and having French parents."LM - MH (p-val)" indicates the p-value for the equivalence test between the coefficient for the Labor Market and the Mental Health treatment.

Table 3: Effect of the treatment on cognitive performance - Multiple Hypothesis Test.

	Dependent variable: Cognitive performance			
	Within test		Within and Between test	
	Piece-rate	Threshold	Piece-rate	Threshold
	(1)	(2)	(3)	(4)
Labor Market	-0.238	0.459	-0.238	0.459
	(0.240)	(0.036)**	(0.240)	(0.036)**
	[0.395]	[0.062]*	[0.523]	[0.133]
Mental Health	-0.227	-0.036	-0.227	-0.036
	(0.269)	(0.871)	(0.269)	(0.871)
	[0.395]	[0.858]	[0.523]	[0.858]
Observations	779	724	779	724
Control Mean	6.97	6.64	6.97	6.64
Baseline Controls	yes	yes	yes	yes

Note: P-values in parentheses. Robust standard errors. * $p < 0.1$, ** $p < 0.05$, *** $p < 0.01$. Adjusted P-values for multiple hypothesis testing in square brackets with 1,000 replications (see (Romano & Wolf, 2005)).The dependent variable is the number of correct matrices. Minimum possible 0, maximum possible 10. Baseline controls include dummy variables for gender, field of study, undergraduate, scholarship recipient, as well as continuous variables for age and number of correct matrices in the first round. Extended controls include dummy variables for the week, day, and time, fatigue, French mother tongue, French nationality, and having French parents.

Table 4: Treatment effect on cognitive performance.

Dependent variable: Cognitive performance		
	(1)	(2)
Labor Market	-0.245	-0.240
	(0.225)	(0.237)
Mental Health	-0.228	-0.235
	(0.267)	(0.246)
Threshold	-0.118	-0.103
	(0.460)	(0.523)
Labor Market X Threshold	0.694**	0.649**
	(0.020)	(0.029)
Mental Health X Threshold	0.193	0.157
	(0.521)	(0.602)
Observations	1503	1503
Control Mean	6.97	6.97
Baseline Controls	yes	yes
Extended controls	no	yes

Note: Robust standard errors in parentheses. Robust standard errors. * $p < 0.1$, ** $p < 0.05$, *** $p < 0.01$. The dependent variable is the number of correct matrices. Minimum possible 0, maximum possible 10. Baseline controls include dummy variables for gender, field of study, undergraduate, scholarship recipient, as well as continuous variables for age and number of correct matrices in the first round. Extended controls include dummy variables for the week, day, and time, fatigue, French mother tongue, French nationality, and having French parents.

Table 5: Treatment effect on cognitive performance (focusing on individuals who paid attention to the treatments).

	Dependent variable: Cognitive performance			
	Piece-rate payment		Threshold payment	
	(1)	(2)	(3)	(4)
Labor Market	-0.205	-0.164	0.584***	0.596***
	(0.329)	(0.441)	(0.009)	(0.009)
Mental Health	-0.290	-0.255	-0.000607	-0.0397
	(0.163)	(0.218)	(0.998)	(0.862)
Observations	685	685	640	640
Control Mean	7.19	7.19	6.77	6.77
Baseline Controls	yes	yes	yes	yes
Extended controls	no	yes	no	yes

Note: P-values in parentheses. Robust standard errors. * $p < 0.1$, ** $p < 0.05$, *** $p < 0.01$. The dependent variable is the number of correct matrices. Minimum possible 0, maximum possible 10. We exclude individuals who answered wrong to more than one comprehension question out of four (13.6% of sample). Baseline controls include dummy variables for gender, field of study, undergraduate, scholarship recipient, as well as continuous variables for age and number of correct matrices in the first round. Extended controls include dummy variables for the week, day, and time, fatigue, French mother tongue, French nationality, and having French parents.

Table 6: Pre-registered heterogeneity: Treatment effect on cognitive performance under piece-rate payment.

	Gender		Scholarship		Level of study		Labor Market	
	Woman	Man	With	Without	1st year	Not 1st year	Close	Not close
	(1)	(2)	(3)	(4)	(5)	(6)	(7)	(8)
Labor Market	-0.168	-0.323	-0.219	-0.204	-0.014	-0.360	0.241	-0.240
	(0.477)	(0.402)	(0.466)	(0.458)	(0.968)	(0.157)	(0.666)	(0.268)
	[0.887]	[0.887]	[0.805]	[0.805]	[0.969]	[0.477]	[0.901]	[0.690]
Mental Health	-0.194	-0.234	0.243	-0.634	-0.150	-0.254	-0.295	-0.123
	(0.448)	(0.501)	(0.405)	(0.028)**	(0.672)	(0.314)	(0.621)	(0.582)
	[0.887]	[0.887]	[0.805]	[0.093]*	[0.897]	[0.685]	[0.901]	[0.901]
Observations	513	266	349	430	257	522	114	665
Control Mean	6.83	6.71	6.50	7.03	6.54	6.91	7.20	6.72

	Field		Depression		Anxiety		Emotion qnt	
	Health Sciences	Others	Above median	Below median	Above median	Below median	After	Before
	(1)	(2)	(3)	(4)	(5)	(6)	(7)	(8)
Labor Market	-0.842	-0.167	-0.331	-0.073	-0.340	-0.190	-0.422	-0.071
	(0.065)**	(0.448)	(0.236)	(0.806)	(0.263)	(0.481)	(0.185)	(0.781)
	[0.190]	[0.681]	[0.522]	[0.941]	[0.590]	[0.720]	[0.448]	[0.938]
Mental Health	-1.244	-0.106	-0.600	0.095	-0.496	-0.070	-0.468	0.010
	(0.016)**	(0.637)	(0.036)**	(0.752)	(0.146)	(0.791)	(0.128)	(0.971)
	[0.080]*	[0.681]	[0.128]	[0.941]	[0.447]	[0.770]	[0.417]	[0.968]
Observations	87	692	379	400	309	470	372	407
Control Mean	7.47	6.71	6.72	6.86	6.47	7.00	6.75	6.83

Note: P-values in parentheses. Robust standard errors. * $p < 0.1$, ** $p < 0.05$, *** $p < 0.01$. Adjusted P-values for multiple hypothesis testing in square brackets with 1,000 replications (see (Romano & Wolf, 2005)). The dependent variable is the number of correct matrices. Minimum possible 0, maximum possible 10. All specifications include baseline controls: gender, field of study, undergraduate, scholarship recipient, as well as continuous variables for age and number of correct matrices in the first round.

Table 7: Pre-registered heterogeneity: Treatment effect on cognitive performance under threshold payment.

	Gender		Scholarship		Level of study		Labor Market	
	Woman	Man	With	Without	1st year	Not 1st year	Close	Not close
	(1)	(2)	(3)	(4)	(5)	(6)	(7)	(8)
Labor Market	0.601	0.191	0.459	0.488	0.364	0.540	0.203	0.521
	(0.021)**	(0.638)	(0.172)	(0.094)*	(0.341)	(0.042)**	(0.694)	(0.030)**
	[0.092]*	[0.935]	[0.437]	[0.328]	[0.710]	[0.145]	[0.963]	[0.129]
Mental Health	-0.037	-0.040	-0.234	0.114	-0.153	0.010	-0.141	-0.032
	(0.890)	(0.921)	(0.495)	(0.694)	(0.666)	(0.972)	(0.822)	(0.894)
	[0.986]	[0.986]	[0.764]	[0.764]	[0.874]	[0.972]	[0.966]	[0.966]
Observations	486	238	321	403	251	473	96	628
Control Mean	6.92	6.47	6.41	7.07	6.57	6.88	7.21	6.71

	Field		Depression		Anxiety		Emotion qnt	
	Health Sciences	Others	Above median	Below median	Above median	Below median	After	Before
	(1)	(2)	(3)	(4)	(5)	(6)	(7)	(8)
Labor Market	1.244	0.372	0.498	0.476	0.561	0.370	0.360	0.516
	(0.090)*	(0.101)	(0.143)	(0.097)*	(0.086)*	(0.188)	(0.251)	(0.092)*
	[0.319]	[0.319]	[0.353]	[0.325]	[0.307]	[0.444]	[0.569]	[0.300]
Mental Health	0.171	-0.072	-0.367	0.304	-0.363	0.200	-0.178	0.114
	(0.829)	(0.754)	(0.270)	(0.312)	(0.327)	(0.466)	(0.563)	(0.721)
	[0.927]	[0.927]	[0.436]	[0.436]	[0.526]	[0.526]	[0.785]	[0.785]
Observations	74	650	345	379	307	417	365	359
Control Mean	6.99	6.75	6.89	6.67	6.89	6.70	6.88	6.67

Note: P-values in parentheses. Robust standard errors. * $p < 0.1$, ** $p < 0.05$, *** $p < 0.01$. Adjusted P-values for multiple hypothesis testing in square brackets with 1,000 replications (see (Romano & Wolf, 2005)). The dependent variable is the number of correct matrices. Minimum possible 0, maximum possible 10. All specifications include baseline controls: gender, field of study, undergraduate, scholarship recipient, as well as continuous variables for age and number of correct matrices in the first round.

Table 8: Treatment effect by depression level (corrected depression score).

Dependent variable: Cognitive performance	Piece-rate payment		Threshold payment	
	Above median	Below median	Above median	Below median
	(1)	(2)	(3)	(4)
Labor Market	-0.352	-0.0496	0.502	0.464
	(0.203)	(0.870)	(0.120)	(0.124)
Mental Health	-0.721**	0.213	-0.366	0.305
	(0.014)	(0.468)	(0.271)	(0.310)
Observations	398	381	352	372
Control Mean	7.10	6.83	6.90	6.39

Note: P-values in parentheses. Robust standard errors. * $p < 0.1$, ** $p < 0.05$, *** $p < 0.01$. To account for the negative treatment effect on the Mental Health score, we regressed the treatment topic, payment treatment, and their interaction with the mental health score. Next, we retrieved the residuals, which will contain everything that is not explained by the treatment effects. Afterwards, we split the sample into two groups (below and above the median) using the residuals as the new mental health score. All specifications include baseline controls: gender, field of study, undergraduate, scholarship recipient, as well as continuous variables for age and number of correct matrices in the first round.

Table 9: Treatment effect on mental health score - Quantile regression.

Dependent variable: Mental health score	All	Piece-rate	Threshold
	(1)	(2)	(3)
Q25			
Labor Market	0.000	0.736**	-0.540
	(1.000)	(0.047)	(0.270)
Mental Health	0.360	0.614	-0.180
	(0.395)	(0.300)	(0.637)
Q50			
Labor Market	-0.277	0.635	-0.470
	(0.564)	(0.419)	(0.431)
Mental Health	0.346	0.476	0.293
	(0.389)	(0.357)	(0.707)
Q75			
Labor Market	0.573	1.440**	-1.132
	(0.353)	(0.018)	(0.320)
Mental Health	1.613**	2.408***	-0.0290
	(0.048)	(0.002)	(0.979)
Observations	1489	767	722
Baseline Controls	yes	yes	yes

Note: P-values in parentheses. Robust standard errors. * $p < 0.1$, ** $p < 0.05$, *** $p < 0.01$. All specifications include baseline controls: gender, field of study, undergraduate, scholarship recipient, as well as continuous variables for age and number of correct matrices in the first round.

Table 10: Treatment effect on cognitive reasoning, risk-taking and maximum WTP.

	Dependent variable:					
	Cognitive reasoning		Risk-taking		Maximum wtp	
	(1)	(2)	(3)	(4)	(5)	(6)
Labor Market	-0.002	0.006	-0.017	-0.012	0.088	0.106*
	(0.978)	(0.925)	(0.784)	(0.851)	(0.161)	(0.089)
Mental Health	-0.004	0.009	-0.054	-0.059	0.072	0.078
	(0.952)	(0.890)	(0.394)	(0.353)	(0.245)	(0.213)
Observations	1503	1503	1503	1503	1503	1503
Control Mean	1.20	1.20	1.56	1.56	0.61	0.61
Baseline Controls	yes	yes	yes	yes	yes	yes
Extended controls	no	yes	no	yes	no	yes

Note: P-values in parentheses. * $p < 0.1$, ** $p < 0.05$, *** $p < 0.01$. From columns (1) and (2) the dependent variable, cognitive reasoning, is the number of correct answers to three questions. From columns (3) and (4) the dependent variable, risk-taking, is the amount of money (up to 3€) they invest in a lottery. From columns (5) and (6) the dependent variable, Max. WTP, is the maximum willingness to pay to participate in a lottery for a coaching program. Baseline controls include dummy variables for gender, field of study, undergraduate, scholarship recipient, as well as continuous variables for age and number of correct matrices in the first round. Extended controls include dummy variables for the week, day, and time, fatigue, French mother tongue, French nationality, and having French parents.

Table 11: Treatment effect on coaching choice.

	Dependent variable:							
	Interview simulation		Work methodology		Self-confidence & Stress Mgmt.		Psychological support	
	(1)	(2)	(3)	(4)	(5)	(6)	(7)	(8)
Labor Market	0.056*	0.064**	-0.007	-0.007	-0.030	-0.026	-0.020	-0.015
	(0.054)	(0.029)	(0.830)	(0.821)	(0.320)	(0.381)	(0.466)	(0.587)
Mental Health	0.020	0.019	-0.005	-0.010	0.006	0.007	0.021	0.028
	(0.478)	(0.496)	(0.863)	(0.752)	(0.839)	(0.807)	(0.436)	(0.316)
Observations	1503	1503	1503	1503	1503	1503	1503	1503
Control Mean	0.26	0.26	0.42	0.42	0.65	0.65	0.24	0.24
Baseline Controls	yes	yes	yes	yes	yes	yes	yes	yes
Extended controls	no	yes	no	yes	no	yes	no	yes

Note: P-values in parentheses. * $p < 0.1$, ** $p < 0.05$, *** $p < 0.01$. This table shows the treatment effect on the demand for coaching. The dependent variable is a dummy equal to 1 for students who chose the respective module as their first option for the coaching session. Baseline controls include dummy variables for gender, field of study, undergraduate, scholarship recipient, as well as continuous variables for age and number of correct matrices in the first round. Extended controls include dummy variables for the week, day, and time, fatigue, French mother tongue, French nationality, and having French parents.

Table 12: Treatment effect on career goals

	Dependent variable: Importance of having			
	Good grades	Good career	University diploma	Enjoyable job
	(1)	(2)	(3)	(4)
Labor Market	0.055	0.176**	-0.027	-0.065
	(0.541)	(0.044)	(0.760)	(0.496)
Mental Health	0.118	0.054	-0.108	-0.057
	(0.172)	(0.539)	(0.224)	(0.541)
Threshold	0.156**	0.149**	-0.019	0.064
	(0.028)	(0.035)	(0.790)	(0.368)
Labor Market X Threshold	-0.002	-0.228*	0.021	0.129
	(0.988)	(0.061)	(0.869)	(0.305)
Mental Health X Threshold	-0.109	-0.145	0.192	0.097
	(0.388)	(0.259)	(0.126)	(0.445)
Observations	1495	1494	1499	1494
Control Mean	3.06	3.68	4.42	4.64

Note: P-values in parentheses. * $p < 0.1$, ** $p < 0.05$, *** $p < 0.01$. Dependent variables range from 1 to 5, where 5 indicates that students rate the respective claim as indispensable. All specifications include baseline controls: gender, field of study, undergraduate, scholarship recipient, as well as continuous variables for age and number of correct matrices in the first round.

Table 13: Treatment effect on depression score and locus of control

	Dependent variable:				
	Depression score	Locus of control	Hard work	Chances are determined	What has to happen will happen
	(1)	(2)	(3)	(4)	(5)
Labor Market	0.134	-0.138	-0.0637	0.0318	0.0506
	(0.115)	(0.148)	(0.144)	(0.457)	(0.223)
Mental Health	0.238***	-0.193**	-0.0803*	0.140***	0.0683*
	(0.005)	(0.032)	(0.065)	(0.000)	(0.084)
Threshold	0.112	-0.0478	-0.0205	0.0359	-0.00106
	(0.101)	(0.507)	(0.550)	(0.293)	(0.975)
Labor Market X Threshold	-0.232*	0.143	-0.00859	-0.0221	-0.0476
	(0.064)	(0.285)	(0.891)	(0.717)	(0.432)
Mental Health X Threshold	-0.198	0.0990	0.0435	-0.144**	-0.00206
	(0.122)	(0.450)	(0.487)	(0.014)	(0.972)
Observations	1489	1453	1503	1503	1503
Control Mean	10.38	19.22	0.64	0.63	0.63

Note: P-values in parentheses. * $p < 0.1$, ** $p < 0.05$, *** $p < 0.01$. This table shows the treatment effect on Depression score and Locus of control. The dependent variable in column (1) is a depression score measured through a short version of the PHG-9. The higher the score, the greater the depression symptoms. Column (2) is the locus of control index (ICI), a higher locus of control indicates that subjects believe they have control over life events. From columns (2) to (4) the dependent variable is a dummy equal to 1 for students who agree with the following statements. Column (3): *To succeed, you have to work hard; success has nothing to do with luck.* Column (4): *The opportunities a person has in life depend on the social conditions in which they live.* Column (5): *I often tell myself that what has to happen will happen somehow.* All specifications include baseline controls: gender, field of study, undergraduate, scholarship recipient, as well as continuous variables for age and number of correct matrices in the first round.

Table 14: Financial struggles and baseline emotional state

	Dependent variable:					
	Feeling good		Feeling awake		Feeling calm	
	(1)	(2)	(3)	(4)	(5)	(6)
Have financial struggles	-0.385***	-0.238***	-0.414***	-0.228***	-0.339***	-0.225***
	(0.000)	(0.003)	(0.000)	(0.000)	(0.000)	(0.002)
Observations	751	751	765	765	761	761
Baseline Controls	yes	yes	yes	yes	yes	yes
Extended Controls	no	yes	no	yes	no	yes

Note: P-values in parentheses. Robust standard errors * $p < 0.1$, ** $p < 0.05$, *** $p < 0.01$. This table shows the association between stated financial struggles and the baseline emotional state. Includes participants who answered the emotional questionnaire before seeing the treatment topics. The dependent variables are the emotional state on three dimensions. The standardized scores are based on four questions for each mood (two positively phrased, two negatively): Feeling calm vs feeling nervous (columns 1 and 2), feeling awake vs feeling tired (columns 3 and 4) and feeling calm vs feeling nervous (columns 5 and 6). Having financial struggles is an indicator for those stating "often" or "nearly every month" struggling to cover their finances. Baseline controls include dummy variables for gender, field of study, undergraduate, scholarship recipient, as well as continuous variables for age and number of correct matrices in the first round. Extended controls include dummy variables for the week, day, and time, fatigue, French mother tongue, French nationality, and having French parents.

Table 15: Balance table on baseline characteristics

		Labor Market vs Controls		Mental Health vs Controls		Threshold vs Piece-rate	
Variable	Mean	Diff	P-values	Diff	P-values	Diff	P-values
Woman	0.66	0.05	0.08*	0.01	0.65	0.01	0.60
Scholarship	0.45	-0.00	0.91	0.05	0.14	-0.00	0.86
Age	21.56	0.04	0.79	-0.10	0.55	-0.19	0.14
First Round Matrices	2.04	0.03	0.72	-0.08	0.29	-0.08	0.22
Undergrad	0.70	-0.00	0.89	-0.00	0.97	0.02	0.37
- *Field of study:*							
Arts and Languages	0.14	-0.00	0.85	0.00	0.84	-0.01	0.68
Health Sciences	0.11	-0.00	0.86	-0.00	0.89	-0.01	0.55
Law, Economics, Management	0.25	0.01	0.66	0.01	0.72	0.01	0.82
Humanities and Social Sciences	0.17	-0.02	0.34	0.01	0.83	0.01	0.72
Science and Technology	0.34	0.02	0.55	-0.02	0.58	0.00	0.85
Observations	1503						
Joint orthogonality test			0.81		0.91		0.83

Note: * $p < 0.1$, ** $p < 0.05$, *** $p < 0.01$. P-values reported are from a t-test of equality of means.

1.2 Figures

Figure 1: Experimental design

		Topic Treatments			
		Labor Market Topic	Mental Health Topic	Control Topic I (Animal Welfare)	Control Topic II (Space Exploration)
**Payment scheme**	Piece-rate payment	N = 374	N = 372	N = 370	N = 478
	Threshold payment	N = 352	N = 356	N = 422	N = 348

Note: Experimental design matrix of the between-subject 2 by 4 design with the corresponding number of observations. We treat each payment scheme separately.

Figure 2: Survey structure

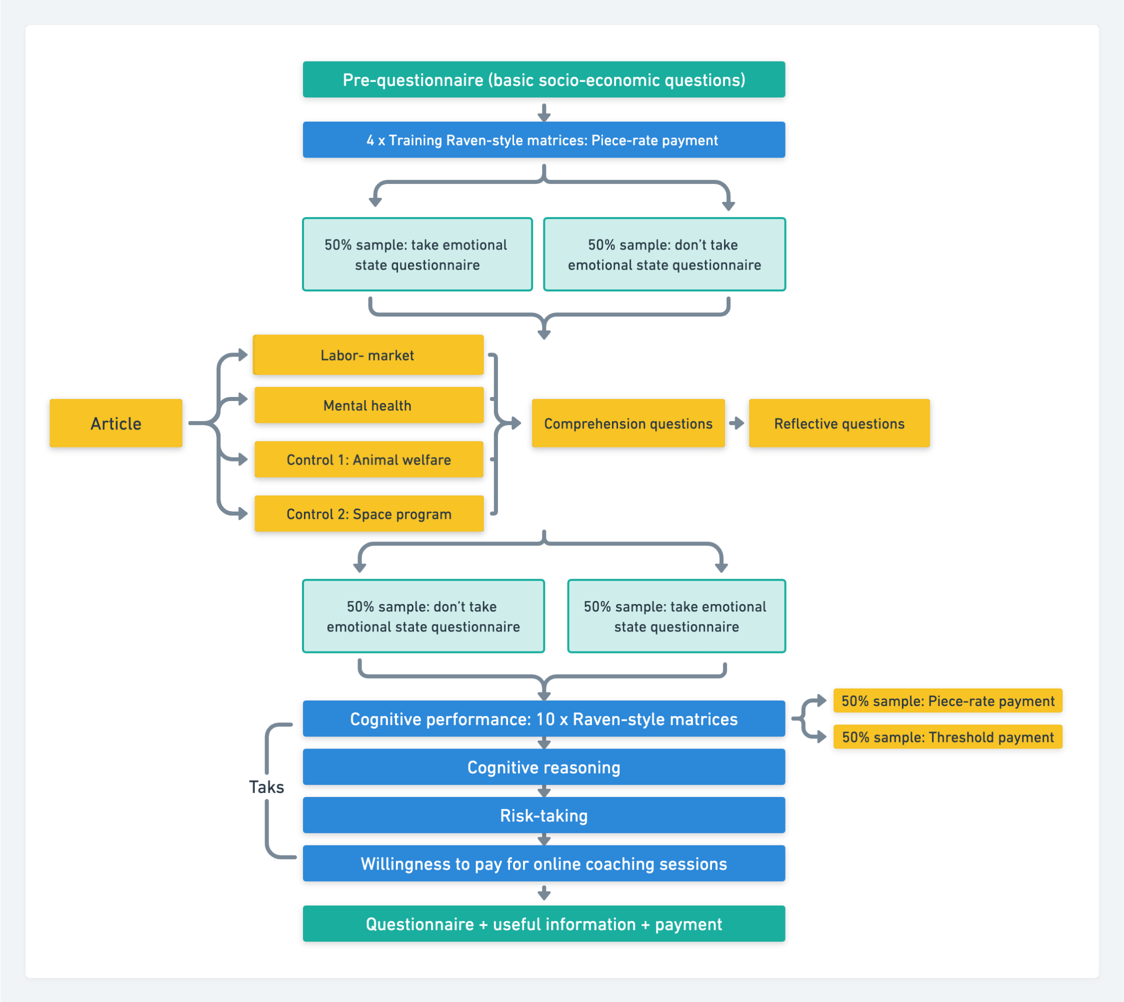

Figure 3: Treatment effect of the Labor Market Treatment on Cognitive Performance by baseline cognitive ability

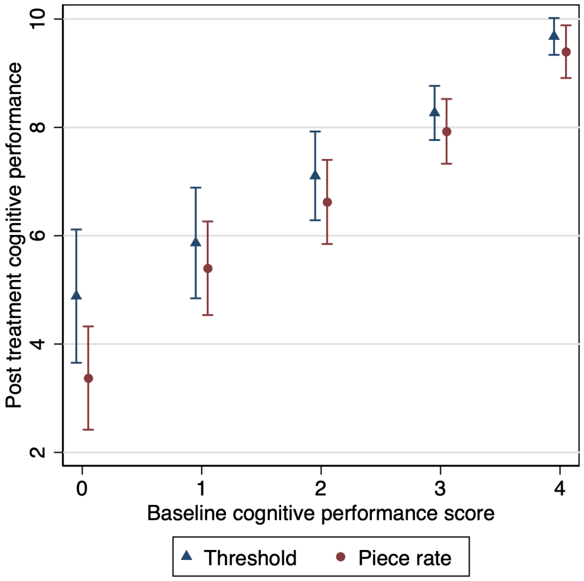

Note: Illustrates the predicted cognitive performance as a function of the baseline cognitive performance score, comparing the threshold payment and the piece-rate payment for control, with 95% confidence intervals. Baseline controls include dummy variables for gender, field of study, undergraduate, scholarship recipient, as well as continuous variables for age and number of correct matrices in the first round

2 Statistical methods and additional results

2.1 Specification for treatment effects

The main analysis was done by OLS in Stata 15. All results are based on regressions including the specified pre-registered controls with robust standard errors. Specifically, we run the following regression:

$$Y_i = \beta_1 treat\_lm_i + \beta_2 treat\_mh_i + X_i + \epsilon_i \quad (1)$$

where Y_i is the outcome of interest (cognitive performance) for individual i, $treat\_lm_i$ is the indicator variable for the labor market treatment, $treat\_mh_i$ is the indicator variable for the mental health treatment, and X_i are the pre-registered control. The pre-registered controls are gender, age, field of study, year of study, number of correct matrices in the first round, and scholarship recipient. Our coefficients of interest are β_1 and β_2. The specification is run separately for those under the threshold payment scheme and those under the piece rate payment scheme.

By controlling for the number of correct matrices in the first round rather than running a difference-in-differences specification, we follow recent recommendations that argue an increase in power with this ANCOVA specification (McKenzie, 2012).

2.2 Comparison of the control treatments

In our study, we pre-registered and used two different control (or placebo) topics. They are both of the same length as the treatment topics, including an article of the same text length, graphs or figures, and comprehension and reflection questions. The goal of the treatment topics was to

occupy respondents for the same time, and make them face a similar task, yet, without worrying them or putting them in a "scarcity mindset".

We chose one topic about animal welfare which is also a controversial topic that young adults discuss and could lead to an emotional response. Yet, it does not directly affect the economic or mental health situation of respondents. We thus hypothesized that it would not make respondent anxious or worried about their situation. Furthermore, we chose the topic of space exploration which is less controversial and was hypothesized to elicit less of an emotional response. Yet, it could make respondents think about how their future might look like, similar to the labour market topic. Again, we hypothesized that it would not make respondent anxious or worried about their situation. The two control topics thus differed in some dimensions (potential emotional response, forward-looking perspective) but not in the most crucial dimension (not making respondents anxious or worried about their own situation). However, the two different topics can allow us to discard some mechanisms.

First, we find that the two control topics do not have a significantly different effect on the different emotional dimensions (see Figure 4 and Tables 16 and 17). We cannot reject the null hypothesis that they have the same effect even on 15 per cent which is the threshold we fixed in the pre-registration plan for the decision to pool the two treatments (see section 4.2). The only significant difference appears in the "tired" dimension and only when we include extended controls.

While power is lower when we do not pool the two control articles, respondents still feel significantly less good and less calm after the treatment topics when compared to either of the control topics (see Table 16 with Animal welfare as control and Table 16 with Space exploration as control).

Furthermore, we find that the two controls do not have a differential effect on the cognitive performance score (see Figures 5 and 6 and Tables 18 and 19). Again, we pool the controls based on the pre-registered 15 per cent threshold.

We find that the positive effect of the labor market topic under the threshold payment holds for both comparisons (see Figures 5 and 6 and Tables 18 and 19). Under piece-rate payment, the coefficients are negative compared to either control group. We find that the Labor Market and Mental Health topic have a negative and significant effect on cognitive performance when compared to the space exploration control only (LM: -0.317 matrices, p-val: 0.039; MH: -0.306 matrices, p-val: 0.049).

Figures

Figure 4: Emotional states before and after the topic treatments with the two control topics separately

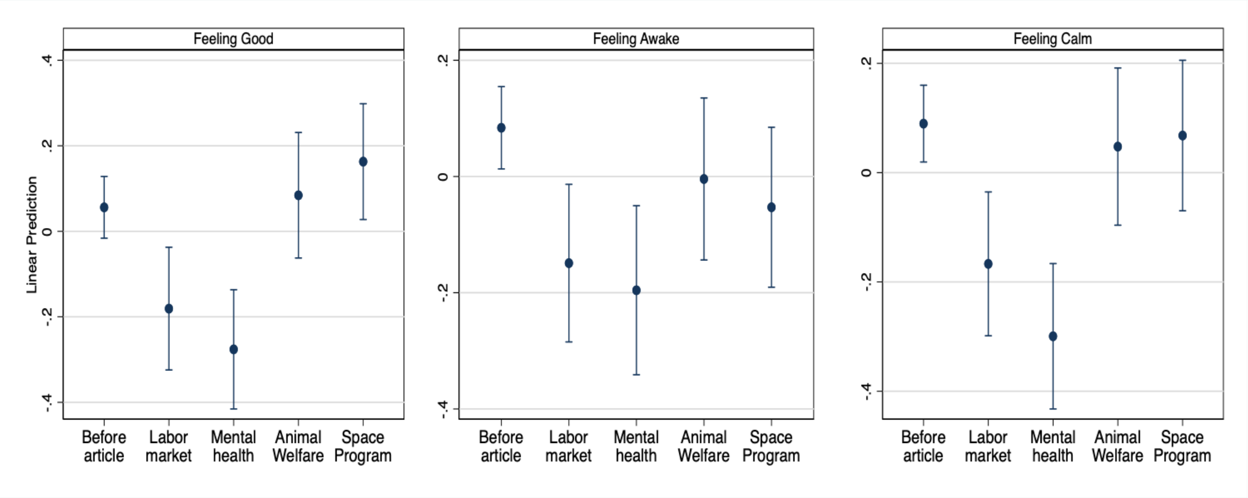

Note: Linear prediction of emotional state before and after the topic treatment, with 95% confidence intervals. The standardized scores are based on four questions for each mood (two positively phrased, two negatively). Includes pre-registered baseline controls: gender, field of study, undergraduate, scholarship recipient, as well as age and number of correct matrices in the first round. See SI Table S1.

Figure 5: Treatment effect on cognitive performance - Animal welfare control as comparison

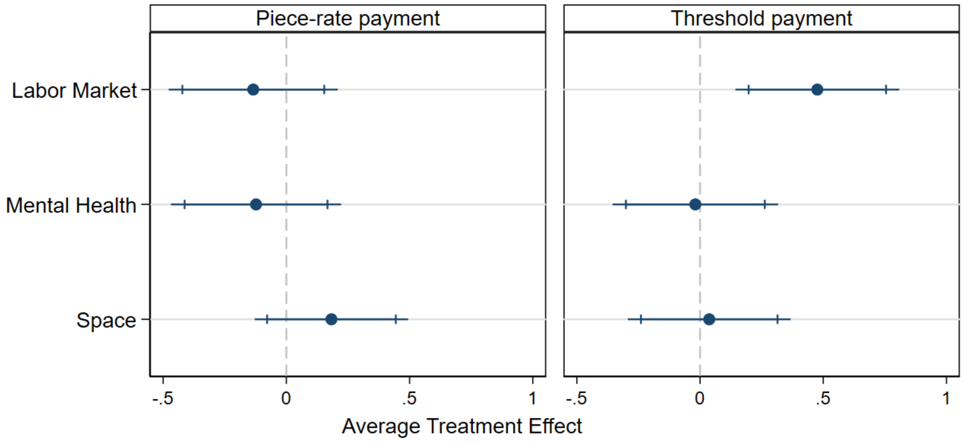

Note: Treatment effects on cognitive performance according to the payment scheme, with 90% and 95% confidence intervals. The dependent variable is the number of correct matrices. Minimum possible 0, maximum possible 10. Includes pre-registered baseline controls: gender, the field of study, undergraduate, scholarship recipient, as well as age and number of correct matrices in the first round. See SI Table S2.

Figure 6: Treatment effect on cognitive performance - Space exploration control as comparison

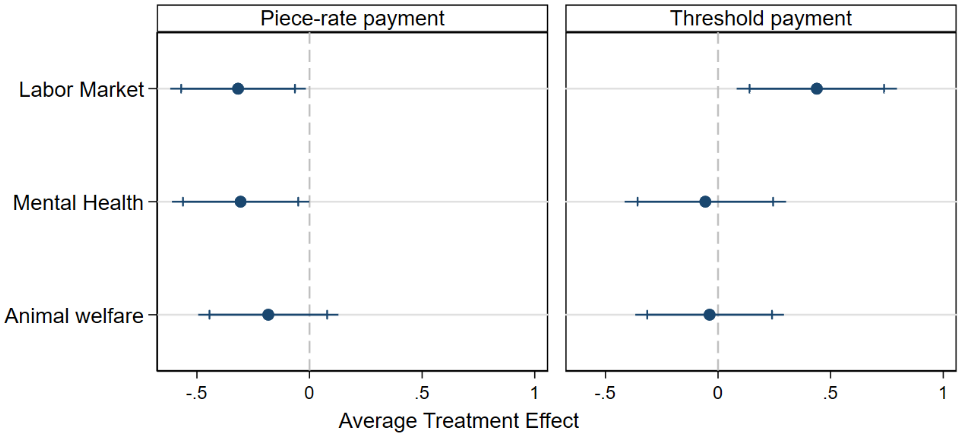

Note: Treatment effects on cognitive performance according to the payment scheme, with 90% and 95% confidence intervals. The dependent variable is the number of correct matrices. Minimum possible 0, maximum possible 10. Includes pre-registered baseline controls: gender, the field of study, undergraduate, scholarship recipient, as well as age and number of correct matrices in the first round. See SI Table S2.

Tables

Table 16: Treatment effect on emotional states: Animal welfare control as comparison

	Dependent variable:					
	Feeling good		Feeling awake		Feeling calm	
	(1)	(2)	(3)	(4)	(5)	(6)
Before Article	-0.0282	-0.0790	0.0881	0.0127	0.0421	-0.00394
	(0.631)	(0.158)	(0.116)	(0.790)	(0.464)	(0.941)
Labor Market	-0.265***	-0.302***	-0.145**	-0.196***	-0.215***	-0.246***
	(0.000)	(0.000)	(0.039)	(0.001)	(0.002)	(0.000)
Mental Health	-0.360***	-0.390***	-0.191***	-0.232***	-0.347***	-0.379***
	(0.000)	(0.000)	(0.008)	(0.000)	(0.000)	(0.000)
Space exploration	0.0787	0.0291	-0.0487	-0.120**	0.0203	-0.0311
	(0.274)	(0.662)	(0.492)	(0.041)	(0.777)	(0.629)
Observations	2964	2964	2994	2994	2984	2984
Control Mean	15.82	15.82	11.96	11.96	12.69	12.69
Baseline Controls	yes	yes	yes	yes	yes	yes
Extended controls	no	yes	no	yes	no	yes

Note: P-values in parentheses. Robust standard errors. * $p < 0.1$, ** $p < 0.05$, *** $p < 0.01$. The standardized scores are based on four questions for each mood (two positively phrased, two negatively). Baseline controls include dummy variables for gender, field of study, undergraduate, scholarship recipient, as well as continuous variables for age and number of correct matrices in the first round. Extended controls include dummy variables for the week, day, and time, fatigue, French mother tongue, French nationality, and having French parents.

Table 17: Treatment effect on emotional states: Space exploration control as comparison

	Dependent variable:					
	Feeling good		Feeling awake		Feeling calm	
	(1)	(2)	(3)	(4)	(5)	(6)
Before Article	-0.107*	-0.108**	0.137**	0.132***	0.0218	0.0271
	(0.053)	(0.027)	(0.014)	(0.003)	(0.696)	(0.578)
Labor Market	-0.344***	-0.331***	-0.0961	-0.0765	-0.235***	-0.215***
	(0.000)	(0.000)	(0.166)	(0.184)	(0.001)	(0.001)
Mental Health	-0.439***	-0.419***	-0.143**	-0.113**	-0.367***	-0.348***
	(0.000)	(0.000)	(0.047)	(0.049)	(0.000)	(0.000)
Animal welfare	-0.0787	-0.0291	0.0487	0.120**	-0.0203	0.0311
	(0.274)	(0.662)	(0.492)	(0.041)	(0.777)	(0.629)
Observations	2964	2964	2994	2994	2984	2984
Control Mean	15.82	15.82	11.96	11.96	12.69	12.69
Baseline Controls	yes	yes	yes	yes	yes	yes
Extended controls	no	yes	no	yes	no	yes

Note: P-values in parentheses. Robust standard errors. * $p < 0.1$, ** $p < 0.05$, *** $p < 0.01$. The standardized scores are based on four questions for each mood (two positively phrased, two negatively). Baseline controls include dummy variables for gender, field of study, undergraduate, scholarship recipient, as well as continuous variables for age and number of correct matrices in the first round. Extended controls include dummy variables for the week, day, and time, fatigue, French mother tongue, French nationality, and having French parents.

Table 18: Treatment effect on cognitive performance: Animal welfare control as comparison

Dependent variable: Cognitive performance				
	Piece-rate payment		Threshold payment	
	(1)	(2)	(3)	(4)
Labor Market	-0.134	-0.141	0.476***	0.457***
	(0.443)	(0.422)	(0.005)	(0.008)
Mental Health	-0.123	-0.117	-0.0191	-0.0745
	(0.485)	(0.502)	(0.911)	(0.670)
Space exploration	0.183	0.177	0.0373	0.0157
	(0.249)	(0.268)	(0.825)	(0.928)
Observations	1558	1558	1448	1448
Control Mean	6.89	6.89	6.77	6.77
Baseline Controls	yes	yes	yes	yes
Extended controls	no	yes	no	yes

Note: P-values in parentheses. Robust standard errors. * $p < 0.1$, ** $p < 0.05$, *** $p < 0.01$. The dependent variable is the number of correct matrices. Minimum possible 0, maximum possible 10. Baseline controls include dummy variables for gender, field of study, undergraduate, scholarship recipient, as well as continuous variables for age and number of correct matrices in the first round. Extended controls include dummy variables for the week, day, and time, fatigue, French mother tongue, French nationality, and having French parents.

Table 19: Treatment effect on cognitive performance: Space exploration control as comparison

Dependent variable: Cognitive performance				
	Piece-rate payment		Threshold payment	
	(1)	(2)	(3)	(4)
Labor Market	-0.317**	-0.318**	0.439**	0.441**
	(0.039)	(0.041)	(0.016)	(0.016)
Mental Health	-0.306**	-0.294*	-0.0563	-0.0902
	(0.049)	(0.056)	(0.758)	(0.630)
Animal welfare	-0.183	-0.177	-0.0373	-0.0157
	(0.249)	(0.268)	(0.825)	(0.928)
Observations	1558	1558	1448	1448
Control Mean	7.03	7.03	6.49	6.49
Baseline Controls	yes	yes	yes	yes
Extended controls	no	yes	no	yes

Note: P-values in parentheses. Robust standard errors. * $p < 0.1$, ** $p < 0.05$, *** $p < 0.01$. The dependent variable is the number of correct matrices. Minimum possible 0, maximum possible 10. Baseline controls include dummy variables for gender, field of study, undergraduate, scholarship recipient, as well as continuous variables for age and number of correct matrices in the first round. Extended controls include dummy variables for the week, day, and time, fatigue, French mother tongue, French nationality, and having French parents.

2.3 Equivalence testing and power calculations

In this subsection, we examine whether the non-significant effects of the treatments are indicative of a true underlying zero effect. A non-significant result does not necessarily validate the null hypothesis of a zero effect as true; instead, it may be attributed to insufficient statistical power to detect a minimal effect. To do so, we employ equivalence testing following Dinno (2017) and Lakens (2017), based on the two-one-sided test (TOST) by Schuirmann (1987).

In conventional practice, the positivist null hypothesis (Ho (+)) is employed to evaluate the rejection of the hypothesis that an effect equals 0. In contrast, when testing for equivalence, one uses a negativist null hypothesis (Ho (-)). This hypothesis assesses whether two quantities differ by at least as much as an equivalence interval, defined by a selected level of tolerance.

For the pre-registration, based on pilot data, we calculated that we would have 80% power to detect an effect of -0.73 matrices at 5 per cent significance. This corresponds to our minimal detectable error (MDE). Ex-post power calculations give us very similar results and an MDE of 0.74 for the mental health treatment (see table 20). We called this as a medium-size effect. We define a small-size effect at half of the MDE which is an effect of -0.37 for the piece-rate treatment and -0.39 for the threshold treatment.

The MDE and half of the MDE in absolute terms are then used as Δ to set the equivalence internals around zero ($0 \pm \Delta$). Therefore, the negativist null hypothesis can be formally stated as Ho (-): $\mid \beta \mid \geq \ \Delta$; where β is the estimated parameter of interest. This formulation leads to two one-sided null hypotheses:

$$H_{O1}\text{(-)}: \ \Delta - \beta \leq 0 \text{ and}$$
$$H_{O2}\text{(-)}: \ \beta + \Delta \leq 0.$$

Table 21 displays the outcomes of the regression test for equivalence for the piece-rate payment. We find that for both treatments, we can reject the presence of a medium-size effect of -0.73/-0.74 determined by the MDE. However, we cannot reject the presence of a small-size effect of -0.37 (determined by half of the MDE). We thus cannot conclude that there is a true zero effect of the treatments on cognitive performance.

Table 22 displays the outcomes of the regression test for equivalence for the threshold payment. For the threshold payment, the treatment effect of the LM treatment is significantly different from zero. For the MH treatment, we again can reject the presence of a medium-size effect, but not the presence of a small-size effect.

Tables

Table 20: Power calculation.

	Estimated effect		Input data				
	Δ	Treat. mean	Control mean	SD control	SD treat.	N control	N treat.
Pre-registration	-0.73	6.81	7.54	2.11	2.98	200	200
Post-registration	-0.74	6.23	6.97	2.97	2.96	415	184

Note: Pre-registration is the power calculation based on pilot data. Post-registration is the power calculation is based on actual data. Both use 80% power and a significance level of 5 per cent. Post-calculation is for the piece-rate payment scheme.

Table 21: Equivalence test - Piece rate payment.

	Dependent variable: Cognitive performance							
	Coef. (1)	Std. Err. (2)	Δ (3)	t1 (4)	t2 (5)	Ho (+) (6)	Ho (-) (7)	Conclude (8)
Panel A: Δ "medium-size effect" (MDE from pre-registration)								
Labor Market	-.238	.202	.73	4.77	2.42	Not Reject	Reject	Equivalence
Mental Health	-.227	.205	.73	4.66	2.45	Not Reject	Reject	Equivalence
Panel B: Δ "medium-size effect" (MDE from post-registration)								
Labor Market	-.238	.218	.74	4.82	2.47	Not Reject	Reject	Equivalence
Mental Health	-.227	.220	.74	4.71	2.50	Not Reject	Reject	Equivalence
Panel C: Δ "small effect" (half of the MDE)								
Labor Market	-.238	.202	.37	3.00	0.65	Not Reject	Not Reject	Not conclusive
Mental Health	-.227	.205	.37	2.91	0.69	Not Reject	Not Reject	Not conclusive

Note: P-values correspond to one-sided test statistics t1 = t of Ho1 ($\Delta - \beta \leq 0$) and t2 = t of Ho2 ($\beta + \Delta \leq 0$). All estimations include dummy variables for gender, field of study, undergraduate, scholarship recipient, as well as continuous variables for age and number of correct matrices in the first round. Extended controls include dummy variables for the week, day, and time, fatigue, French mother tongue, French nationality, and having French parents.

Table 22: Equivalence test - Threshold payment.

	Dependent variable: Cognitive performance							
	Coef. (1)	Std. Err. (2)	Δ (3)	t1 (4)	t2 (5)	Ho (+) (6)	Ho (-) (7)	Conclude (8)
Panel A: Δ "medium-size effect" (MDE from pre-registration)								
Labor Market	.4591	.218	.73	1.24	5.45	Reject	Not Reject	Relevant Difference
Mental Health	-.035	.220	.73	3.47	3.15	Not Reject	Reject	Equivalence
Panel B: Δ "medium-size effect" (MDE from post-registration)								
Labor Market	.4591	.218	.74	1.29	5.50	Reject	Not Reject	Relevant Difference
Mental Health	-.035	.220	.74	3.52	3.19	Not Reject	Reject	Equivalence
Panel C: Δ "small effect" (half of the MDE)								
Labor Market	.4591	.218	.37	-0.41	3.80	Reject	Not Reject	Relevant Difference
Mental Health	-.035	.220	.37	1.84	1.51	Not Reject	Not Reject	Not conclusive

Note: P-values correspond to one-sided test statistics t1 = t of Ho1 (Δ -$\beta \leq 0$) and t2 = t of Ho2 ($\beta + \Delta \leq 0$). All estimations include dummy variables for gender, field of study, undergraduate, scholarship recipient, as well as continuous variables for age and number of correct matrices in the first round. Extended controls include dummy variables for the week, day, and time, fatigue, French mother tongue, French nationality, and having French parents.

2.4 Causal forest

Causal Forest is a non-parametric method that allows uncovering heterogeneity in treatment effects (Wager & Athey, 2018). A causal forest is the average of many different casual trees i.e., a data-driven approach to split the data into subgroups that differ in the magnitude of treatment effects (Athey & Imbens, 2016). Importantly, the goal of this method is to obtain accurate estimates for the Conditional Average Treatment Effect (CATE):

$$\tau(x) = \mathbb{E}[Y_i(1) - Y_i(0)|X_i = x] \tag{2}$$

where Y is the outcome of interest, and X is a vector of observable characteristics. A central feature of this method relies on sample splitting, which allows for obtaining valid asymptotic confidence intervals of the parameters estimated. The idea is to use different sub-samples for split selection and estimation. This type of sample splitting is called honesty (Athey & Imbens, 2016).

Following (Athey & Wager, 2019), we implement the causal forest algorithm as follows:

1. We split the data into 5-folds.
2. We take one fold as a hold-out or test data, and we use the remaining folds as training data to fit a CATE model with 20,000 trees.
3. We use the held-out fold to rank the observations into quartiles according to their CATE

prediction.

4. We cycle through all the folds by applying the same procedure. This sample splitting will ensure that the honesty criterion is met so we can obtain valid tests for each quartile.
5. We fit 100 causal forests and we average out their prediction to guarantee stability in our results.
6. Next, we concatenate the independent rankings together to study the differences between each rank-group.

We apply the causal forest to predict heterogeneity in treatment effects on the cognitive performance outcome for all possible combinations of treatment cells. We assess the quality of the predicted heterogeneity for our primary treatment cells. The first rough test is a histogram of the estimated CATE. Figure 7 presents the results. Under the piece-rate treatment and regardless of the topic treatment, the distribution of the CATE is very concentrated at a point. This suggests that the variables capture little heterogeneity in the underlying results. Little variation in the CATE means that the causal forest could be underpowered and is unable to detect differences in treatment effects (Athey & Wager, 2019). Contrary, we observe that the histogram for any of the topic treatments under the threshold payment is spread out indicating plausible heterogeneity.

Second, Figure 8 displays the average treatment effect within each predicted rank-group as defined above. Except for the labor market and mental health treatment under the threshold payment scheme, the plots are not monotonic or decreasing along the predicted subgroups – meaning that the CATE rankings capture noise rather than heterogeneity.

Third, we use the best linear predictor method to assess whether the suggestive heterogeneity found is meaningful and not pure noise. This calibration test seeks to fit the CATE as a linear function of the held-out causal forest estimates. A coefficient of 1 for the "Mean forest prediction" suggests that the prediction produced by the forest is correct, and a coefficient of 1 for "Differential forest prediction" suggests that the forest is capturing heterogeneity (see (Athey & Wager, 2019) for details on the test). Table 23 summarizes the results. We find that the only treatment with significant underlying heterogeneity is the labor market treatment under the threshold payment. We do not find evidence of differences in treatment effects for the rest of the treatments.

Table 24 displays the different average treatment effects (ATE) for the four CATE rankings. We find that the positive average effect is driven by those in the fourth quartile. Finally, we estimate a differences-in-means model to test whether the prediction for quartiles 2, 3, and 4 is larger than the one in the first quartile. Table 25 summarizes the results. We find that the only quartiles that significantly differ from each other are quartiles 1 and 4 – while those in the fourth perceived a positive and significant boost from the treatment, those in the first quartile did not. Therefore, we compare the characteristics of these two contrasting groups.

Tables

Table 23: Best linear predictor

	Labor Market		Mental Health	
	Threshold (1)	Piece-rate (2)	Threshold (3)	Piece-rate (4)
Mean forest prediction	0.996***	1.146*	0.550	1.147**
	(0.197)	(0.722)	(0.651)	(0.685)
Differential forest prediction	1.201*	-2.600	-0.192	-1.617
	(0.841)	(1.021)	(0.630)	(1.028)

Note: Standard errors in parentheses. * $p < 0.1$, ** $p < 0.05$, *** $p < 0.01$. This table reports the best linear predictor for each of the primary treatment cell

Table 24: ATE on cognitive performance for Mental health- Threshold treatment

Ranking (1)	Estimate (2)	Std- Error (3)	P-value (4)
Quartile1	-0.347	0.524	0.508
Quartile2	0.574	0.512	0.263
Quartile3	0.778	0.518	0.133
Quartile4	1.187	0.507	0.020**

Note: * $p < 0.1$, ** $p < 0.05$, *** $p < 0.01$. This table reports the average treatment effects of the labor market treatment and threshold payment scheme on cognitive performance for each group obtained with the conditional average treatment estimated using the causal forest algorithm.

Table 25: Difference-in-means between quartiles

	Estimate (1)	Std. Error (2)	P-value (3)	Adj. P-value (4)
Quartile 2 - Quartile 1	0.920	0.747	0.219	0.218
Quartile 3 - Quartile 1	1.125	0.712	0.115	0.197
Quartile 4 - Quartile 1	1.534	0.733	0.037**	0.091*

Note: This table reports a difference-in-means estimator, where we test if the predictor for quartiles 2,3 and 4 is statistically different from quartile 1. Column (4) reports adjusted P-values for multiple hypotheses testing with 1,000 replications (see (Romano & Wolf, 2005)). * $p < 0.1$, ** $p < 0.05$, *** $p < 0.01$.

Figures

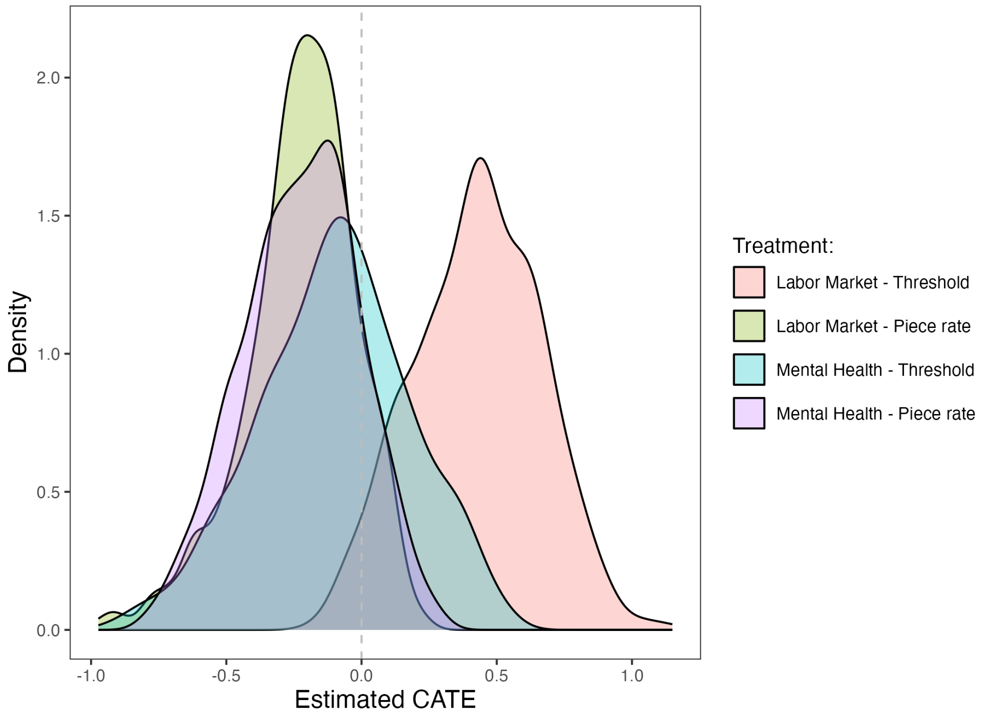

Figure 7: Histogram Estimated CATE

Distribution of the Conditional Treatment Effects estimated by the causal forest from Wager and Athey (2018). Comparison is the control treatments under the same payment scheme.

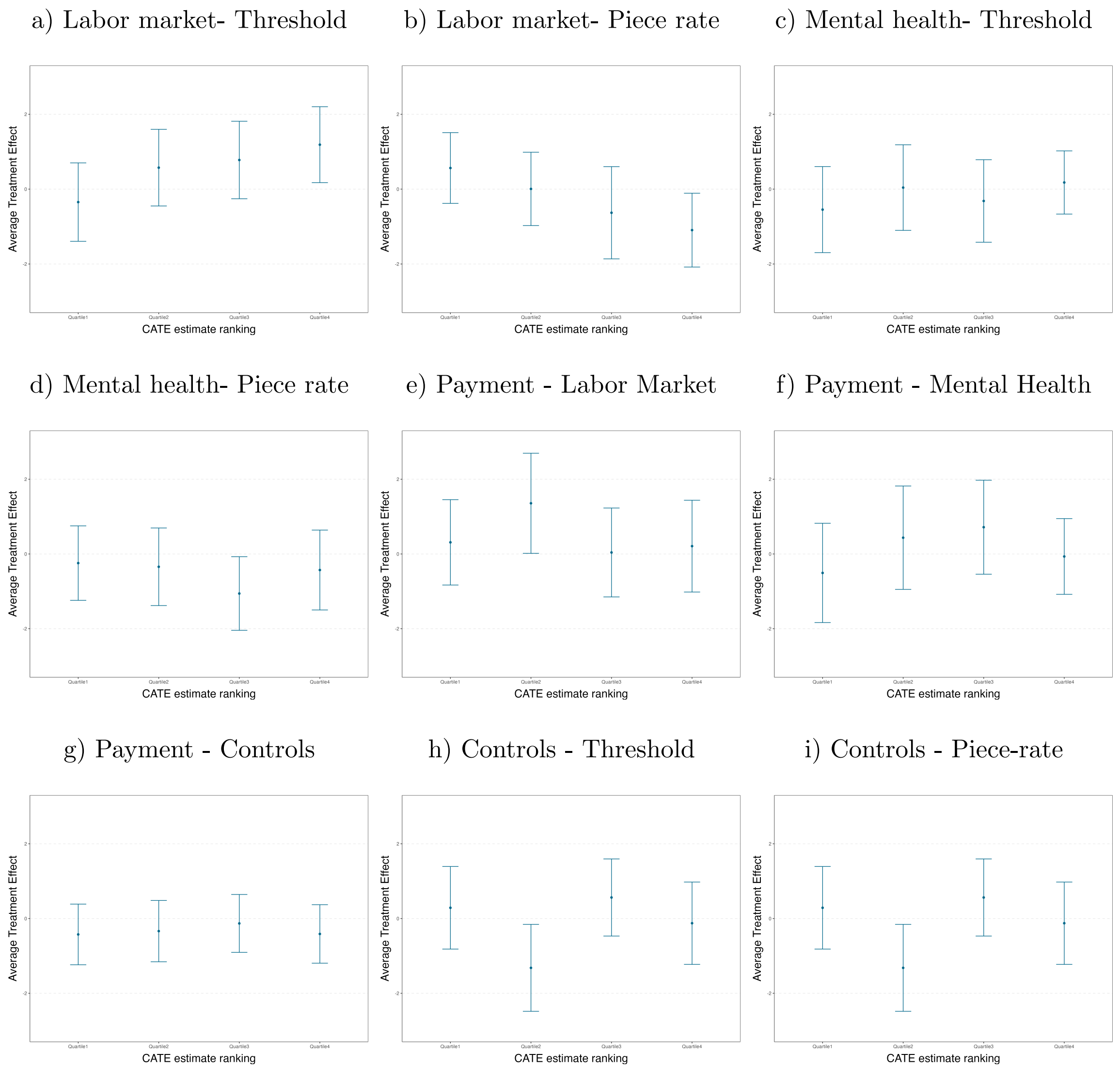

Figure 8: Average Treatment Effect on cognitive performance

Note: Average treatment effects of the different treatment cells, grouped by the quartiles based on the conditional average treatment estimated using the causal forest algorithm with 95% confidence intervals.

3 Treatment topics (translated)

3.1 Labor market

Here is a news article that we ask you to read carefully. It is an excerpt from the article “Covid-19: when diplomas no longer help you enter the job market” published on 10/12/2020 on the website of France Culture. We will then ask you some questions about the text. You can come back to the text to answer the questions.

Covid-19: when diplomas no longer help to enter the job market.

Four out of ten young people have changed their career plans because of the Covid-19 crisis: for young graduates, getting into the job market is more complex than expected today. The number of job offers available to young graduates has dropped by almost 40% in 2020, compared to the previous year: this is one of the consequences of Covid-19 on 18-24 year olds. According to a recent survey by the Elabe Institute, 84% of this age group believe that their generation will experience a period of higher unemployment than previous generations (see infographic below).

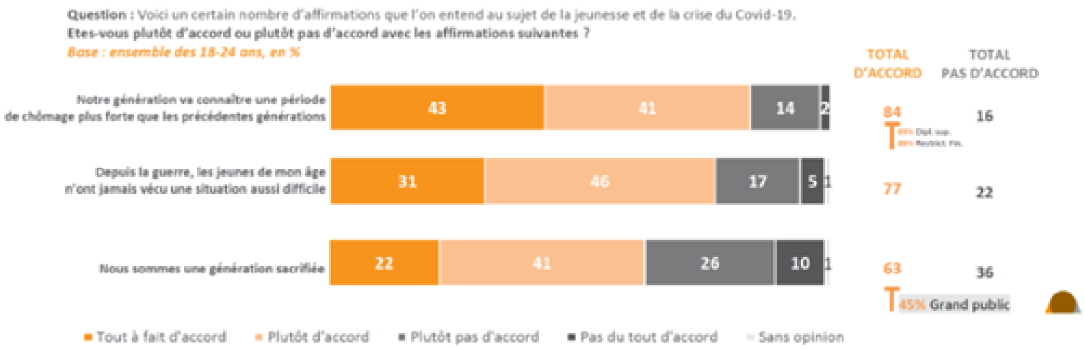

Source of infographic: ELABE, Les Français et la jeunesse Post-Covid – Sondage ELABE pour le Cercle des Economistes, 10 December 2020.

When she started her third year of studies in tourism, alternating in a hotel in Montpellier, Elisa did not think that her professional integration would be so difficult: the sector was doing well, she had a first professional experience, she was passionate... Then the Covid-19 crisis arrived: France was confined for the first time, the hotel closed down, her courses went digital. The young woman hangs on, and in the summer, she can finish her apprenticeship. But at the end of August, her contract ended: ”I looked for a job, but it was already difficult because it was the end of the season, and there were still effects of the health crisis,” she explains. ”There, to be honest, I gave up looking in my field altogether: with the new containment, with the crisis at the moment, all the businesses are closed, people don’t go on trips anymore, hotels don’t have customers, they are closed.” Today, she survives by gnawing on her savings. For lack of a first job in her dream sector, she is looking for a food job in the sales sector. And her case is far from isolated: many young people are struggling to find a job after graduation this year, and not just in the sectors most affected by the coronavirus crisis. [...]

”It’s a complete reversal of the situation,” explains Gilles Gateau, director general of Apec, the Association for the Employment of Executives. ”Until now, 85% of young graduates were able to find a job. We measure it at Apec through the job offers that are submitted: these job offers for young graduates, accessible to beginners, who have no experience, have decreased by 39% over the period from January to November this year compared to the same period last year, he calculates.

While it's also down for the more experienced executives... But it's down by 28%, so you can see that young graduates are more affected: even with the right qualifications, it's becoming much more difficult than last year to get your first permanent job.

According to the Observatoire français des conjonctures économiques (OFCE), unemployment will continue to rise in 2021, exceeding 10% of the active population (see graph below).

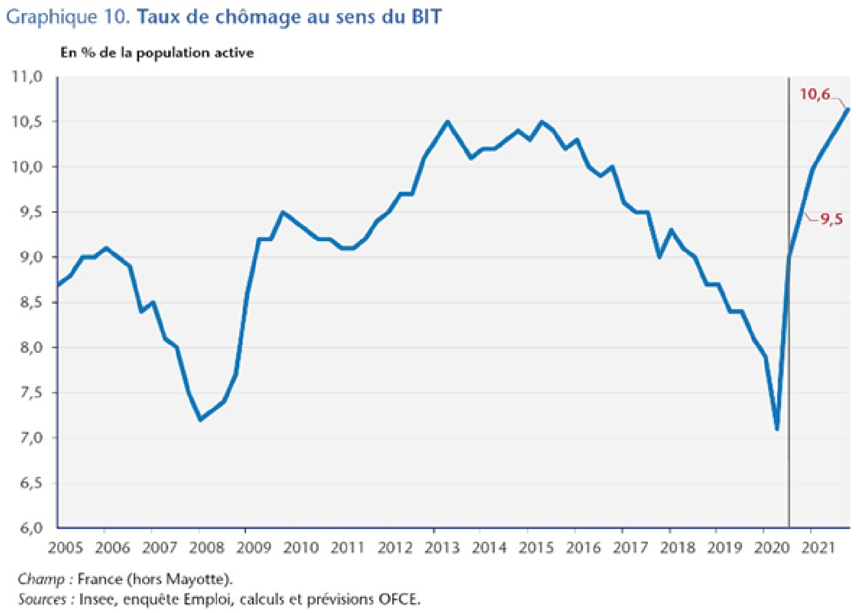

Source of graph: OFCE, Policy Brief 81, 11 December 2020.

Choose the sentence that best summarizes the text from the suggestions below. Please select only one of the following:

1. Despite the current difficult situation, a strong economic rebound is expected in 2021.
2. The current economic crisis is affecting young people less than older people.
3. Young people aged 18-24 rightly expect significant difficulties in finding a job in the next few years.
4. 18-24 year olds are too pessimistic about their short-term economic opportunities.

According to the article, how have job openings for young graduates changed in 2020 compared to the previous year? Please select only one of the following:

1. They decreased by 85%.
2. They decreased by 39%.
3. They increased by 10%.
4. They decreased by 28%.

According to the article and the infographic, what is the opinion of 18-24 year olds in France? Please select only one of the following:

1. More than 80% believe that they will have a harder time finding a job than the previous generation.
2. Slightly less than 20% feel they are a sacrificed generation.
3. More than 80% feel that they are a sacrificed generation.
4. Slightly less than 40% believe they will experience a higher period of unemployment than the previous generation.

According to the article and the OFCE graph, how will the unemployment rate evolve in 2021 in France? Please select only one of the following:

1. It will gradually decrease towards its pre-crisis level.
2. It will increase exponentially until at least 2022.
3. It will gradually decrease to about 10%.
4. It will continue to increase until it exceeds the records observed in the first half of the 2010s.

When do you think the economic recession will end and the French economy will start growing again? Please select only one of the following:

1. 1st quarter 2021
2. 2nd quarter 2021
3. 3rd quarter 2021
4. 4th quarter 2021
5. 1st quarter 2022
6. 2nd quarter 2022
7. 3rd quarter 2022
8. 4th quarter 2022
9. not before 2023
10. never
11. I don't know

Did you find the article interesting? Please select only one of the following:

1. very interesting
2. interesting
3. not very interesting
4. very uninteresting

Did you learn any new information? Please select only one of the following:

1. I learned a lot (I knew little/nothing about the topic of the text).
2. I learned some new information (I knew a little about the topic of the text).
3. I did not learn anything (I knew well the topic of the text).

On a scale of 1 to 4, where 1 means "I completely disagree" and 4 means "I completely agree", please indicate your opinion about the sentences below.

- The economic consequences of the health crisis decrease my chances of finding a stable job (permanent) that corresponds to my interests.
- My family's overall economic situation has worsened since the Covid-19 crisis began.
- The health crisis has increased my financial concerns.
- It is likely that I will not be financially independent right after college and will have to rely on family assistance or state benefits.
- The economic consequences of the health crisis decrease my chances of finding a job quickly after graduation.

3.2 Mental health.

Here is a news article that we ask you to read carefully. It is an excerpt from the article "'This new lockdown is the double punishment. We are locked up and on top of that, it's our fault': A youth in psychological distress" published on 10/11/2020 by Le Monde. We will then ask you some questions about the text. You can come back to the text to answer the questions.

"This new lockdown is the double punishment. We are locked up and on top of that, it's our fault": A youth in psychological distress.

Since the end of high school, Sofia has been consulting a psychiatrist to calm her anxiety attacks. Because she suffered so much from the first lockdown, she ended up giving up her mathematics degree. At the beginning of the new school year, the 19-year-old woman reoriented herself towards a degree in applied foreign languages: "Even in normal times, the idea of not succeeding makes me anxious. At the moment, I'm much more afraid of dropping out", she explains, disturbed by the fact that all her classes were held remotely and that she didn't have the time to bond with her new class. Particularly affected by the consequences of the current health crisis, young people accumulate risk factors in terms of the deterioration of their psychological balance: the switch to distance learning in higher education places some young people in a great solitude; with the disappearance of a large part of their internships and other small jobs, the precariousness of students is growing, as well as a strong feeling of anxiety about the future. According to a large national survey conducted by the Observatoire de la vie étudiante (OVE), half of the students have already suffered from loneliness or isolation during their first lockdown (see graph below). And nearly one in three students shows signs of psychological distress.

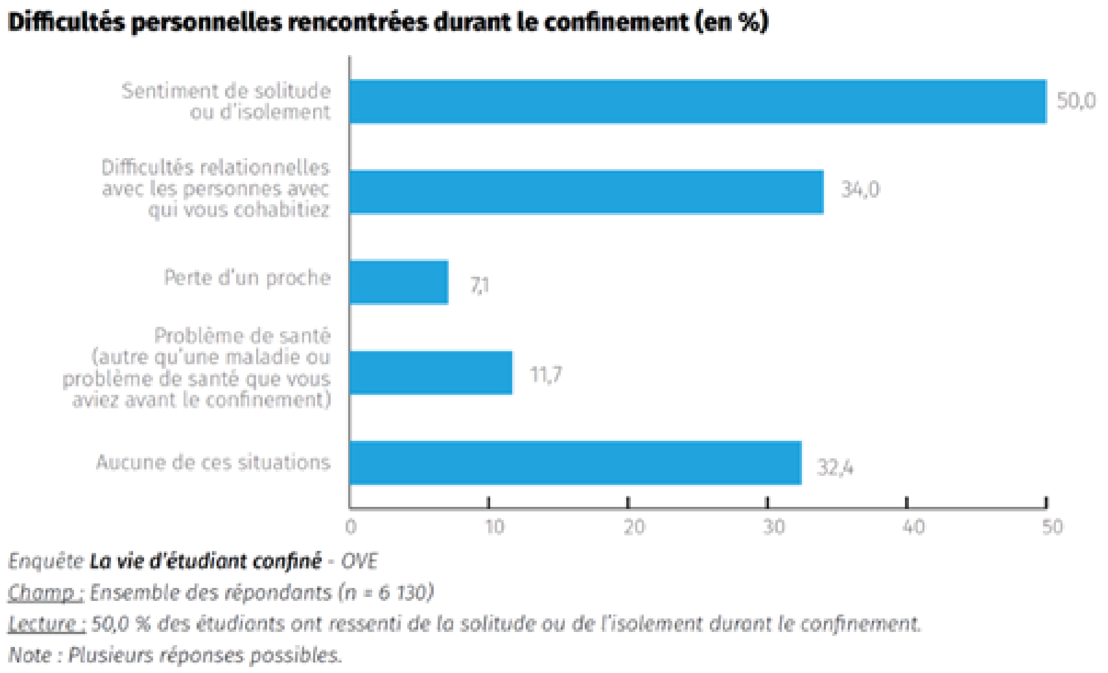

Source of graph: OVE, Life as a Confined Student - Results of the Survey on Living Conditions during the Health Crisis, September 2020.

"We've seen a very clear increase in requests for consultation since the start of the school year", says Christophe Ferveur, a psychologist with the French Student Health Foundation and president of the Psychiatric and Psychological Care Network for Students (Resppet). "The cases are more numerous, but also more worrying than usual. Dissocialized, some young people get depressed and gradually become isolated. We have to intervene very quickly so that they can get out of the impasse." But in order to intervene quickly, there is the question of seeking care. "When you are laid up in bed, you don't have the strength to ask for help. The more one feels bad, the less one speaks about it: it is the specificity of the psychological disorders", Christophe Ferveur hammers. The conclusions of the first lockdown are alarming on the subject. A study conducted by the National Center for Resources and Resilience (CN2R), for which nearly 70,000 students were surveyed, reveals severe scores of distress (22.4%), anxiety (27.5%), intense stress (24.7%), depression (16.1%) and suicidal thoughts (11.4%). But only 12.4% of those with at least one of these disorders reported seeking professional help (2.7% contacted the university health service). While young people are rather spared from severe forms of Covid-19, the effects of successive lockdowns and ambient uncertainty on their mental health are worrying, as shown by the repeated CoviPrev survey of Santé publique France (see graph below).

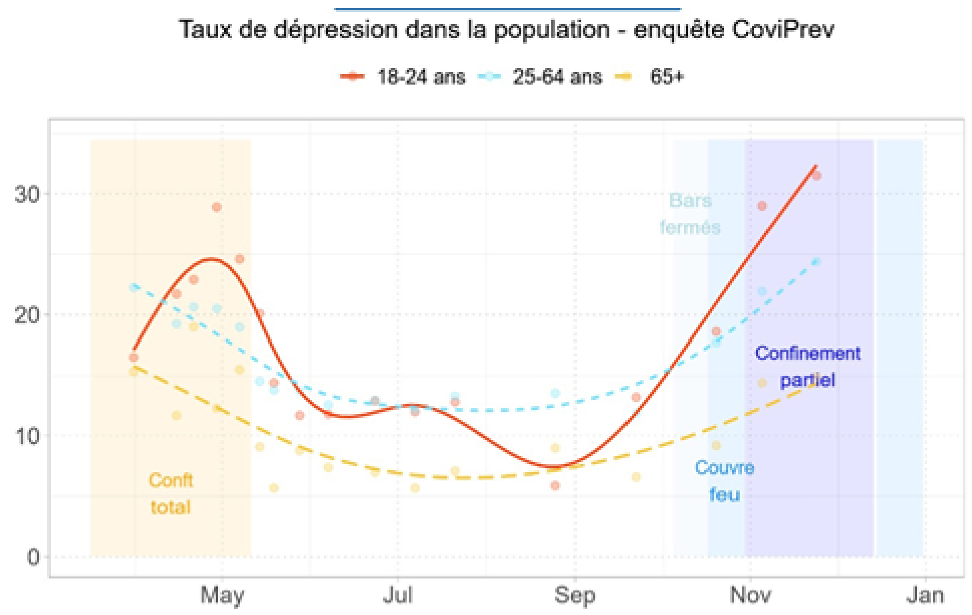

Proportions de dépressions dans la population, enquête CoViPrev (dépression : niveau supérieur à 10 sur l'échelle HAD – Hospital Anxiety and Depression scale). La théorie prédit que les plus jeunes seront les plus affectés par le confinement et les restrictions à l'activité économique. Les enquêtes le confirment.

Source of graph: Samuel J. Sender, CoViD-age, https://samjs.shinyapps.io/CoViD – accessed on 06/01/2021.

Choose the sentence that best summarizes the text from the suggestions below. Please select only one of the following:

1. Distance learning and isolation obligations are generating a sharp increase in psychological distress for many higher education students.
2. Students have become comfortable with digital learning tools and a significant portion of them value distance learning courses for the flexibility they provide.
3. Over the past year, there has been a proportional increase in psychological counseling and depressive symptoms in the student population.
4. Many students do not understand why they are deprived of a social life when they are not at significant risk of developing a severe form of Covid-19.

From the list below, which of the following is a risk factor for the mental health of young people cited in the article? Please select only one of the following:

1. Dependence on parents
2. Lack of physical activity
3. Shaming from society
4. Worry about the future

According to the CVO, what proportion of students experienced loneliness or isolation during the first lockdown? Please select only one of the following:

1. 40

2. 50
3. 31
4. 60

According to the CN2R, what proportion of students reported having suicidal thoughts during the first lockdown? Please select only one of the following:

1. 24,7
2. 11,4
3. 16,1
4. 27,5

When do you think our social activities will resume normally and we will be able to abandon the barrier gestures? Please select only one of the following:

1. 1st quarter 2021
2. 2nd quarter 2021
3. 3rd quarter 2021
4. 4th quarter 2021
5. 1st quarter 2022
6. 2nd quarter 2022
7. 3rd quarter 2022
8. 4th quarter 2022
9. not before 2023
10. never
11. I don't know

Did you find the article interesting? Please select only one of the following:

1. very interesting
2. interesting
3. not very interesting
4. very uninteresting

Did you learn any new information? Please select only one of the following:

1. I learned a lot (I knew little/nothing about the topic of the text).
2. I learned some new information (I knew a little about the topic of the text).

3. I did not learn anything (I knew well the topic of the text).

On a scale of 1 to 4, where 1 means "I completely disagree" and 4 means "I completely agree", please indicate your opinion about the sentences below.

- The health crisis has prevented or spoiled important celebrations for me (birthdays, holiday parties, family celebrations...).
- I am likely to see a mental health professional in the next 6 months.
- Having to attend classes remotely and not face-to-face has created a great sense of loneliness or isolation for me.
- The periods of lockdown generated a significant level of stress for me.
- The health crisis prevented me from having a good time with other students.

3.3 Animal welfare.

Here is a news article that we ask you to read carefully. It is an excerpt from the article "Une proposition de loi pour le bien-être animal débattue ce jeudi à l'Assemblée Nationale" published by La Dépêche on 01/01/2020. We will then ask you some questions about the text. You can come back to the text to answer the questions.

A law proposal for animal welfare debated this Thursday at the National Assembly.
A bill in favor of animal welfare, notably carried by the mathematician Cédric Villani is debated today in committee at the National Assembly. The animal cause is moving forward. In the aftermath of the announcements of the Minister of Ecological Transition, Barbara Pompili, in favor of captive wildlife - on dolphinariums, circuses and traveling shows, mink farms and zoos - the National Assembly will examine from this Thursday in committee (and October 8 in the hemicycle) the proposed law on animal welfare, carried by the LREM deputy Cédric Villani and several of his colleagues, including Matthieu Orphelin, close to Nicolas Hulot, or Paula Forteza, deputies of the new group Ecology, Democracy, Solidarity (EDS). Indeed, this law, which "takes up and adapts a large part of the project of Referendum for animals initiated by a collective of forty associations as well as dozens of personalities from the economic, cultural or political world", intends in six articles to attack animal suffering. In addition to the prohibition of "the breeding of animals for the purpose of obtaining fur from January 1, 2025" and the prohibition of "live animal shows of non-domestic species within five years from the promulgation of the law" - subjects taken up by Ms. Pompili - the deputies propose to draw a line on "hunting at hounds and equivalent hunting practices, as well as the so-called traditional hunts". In a context where the consumption of chicken meat, for example, has doubled in 20 years (see graph below), they also propose to progressively eliminate - until 2040 - cage rearing in order to "only retain practices that are compatible with the respect of the biological imperatives of the animals".

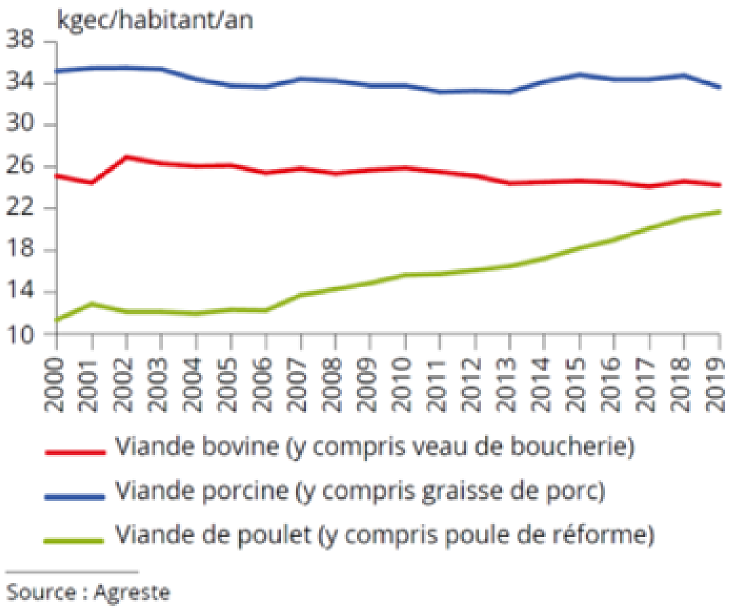

(Source of graph: Agreste, Synthèses conjoncturelles, no. 359, June 2020.)

Initially, "the construction of any new livestock building that does not provide animals with access to the open air adapted to their needs will be prohibited. The end of cage rearing of laying hens could occur as early as 2025", according to the MEPs, who point out that "400 farms use cages, which represents two out of three hens, or 33 million animals". In a second step, the deputies propose "a concerted approach involving all stakeholders in the sectors concerned, with a view to arriving at a governmental strategy organizing the gradual abandonment of cage farming by 2030". This concerns, in addition to chickens, rabbits, pregnant sows, laying quails and calves. The fact remains that the animal cause has undeniably gained ground in recent years. "82% of French people say they are in favor of banning cage farming within five years, 91% are in favor of mandatory outdoor access for all farm animals and 82% say they are against hunting with hounds", point out the deputies who co-signed the bill (see infographic below), while the Referendum for Animals has been signed by more than 784,000 people.

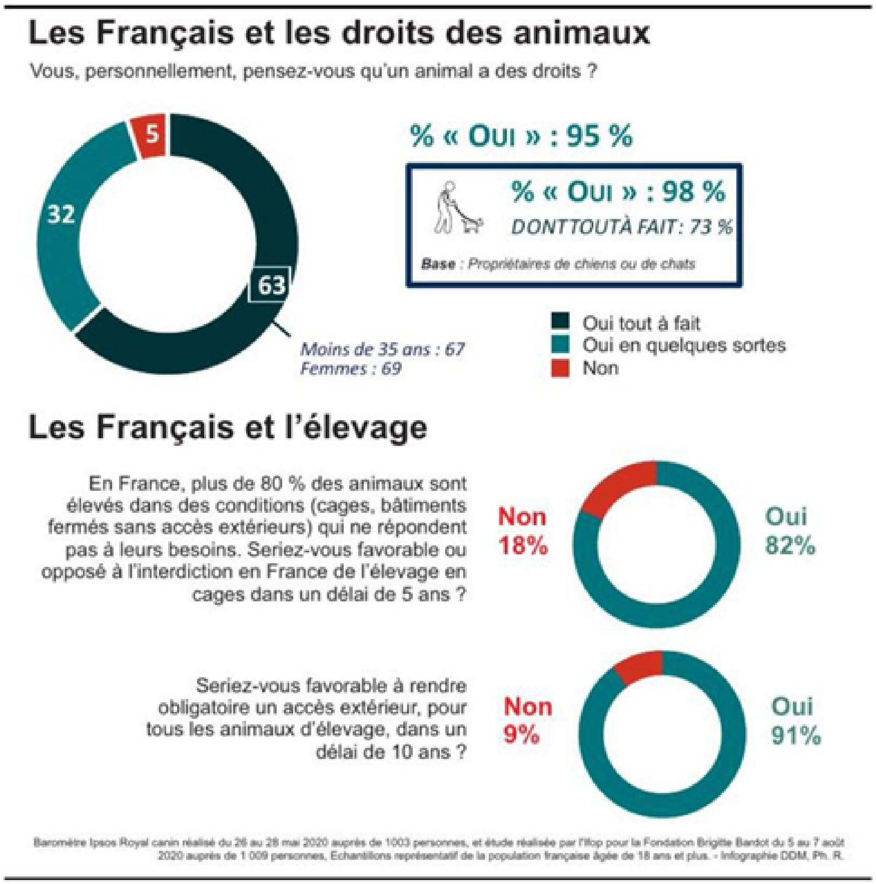

Choose the sentence that best summarizes the text from the suggestions below. Please select only one of the following:

1. A new law to end hunting and cage farming has just been passed by the National Assembly.
2. Several deputies have filed a bill to end hunting and cage farming.
3. The Minister of Ecological Transition, Barbara Pompili, has decided to put an end to hound hunting and cage farming as of January 1, 2025.
4. Members of the National Assembly conducted a survey of the French and found that more than 80% were in favor of ending cage farming.

According to the article, how many chickens are currently raised in cages in France? Please select only one of the following:

1. 10 million
2. 22 million
3. 27 million
4. 33 million

According to the article and the graph, the consumption of chicken meat... Please select only one of the following:

1. has been slowly decreasing over the past 10 years
2. doubled in 10 years
3. doubled in 20 years
4. has remained stable over the past 10 years

According to the article, what proportion of laying hens live in cages? Please select only one of the following:

1. 1/3
2. 2/3
3. 1/4
4. 1/2

According to the article and the infographic, what proportion of French people say they are in favor of banning cage farming within five years? Please select only one of the following:

1. 91%
2. 82%
3. 80%
4. 95%

Did you find the article interesting? Please select only one of the following:

1. very interesting
2. interesting
3. not very interesting
4. very uninteresting

Did you learn any new information? Please select only one of the following:

1. I learned a lot (I knew little/nothing about the topic of the text).
2. I learned some new information (I knew a little about the topic of the text).
3. I did not learn anything (I knew well the topic of the text).

On a scale of 1 to 4, where 1 means “I completely disagree” and 4 means “I completely agree”, please indicate your opinion about the sentences below.

- It is important that all livestock have access to the outdoors.
- I am likely to attend a “non-domestic live animal show” in the coming year.
- Caged animals produce poor quality food.
- I take into account animal welfare criteria in my consumption choices.
- I limit my meat consumption because factory farming does not respect animal welfare.

3.4 Space exploration.

Here is a news article that we ask you to read carefully. It is an excerpt from the article “Back to the Moon: Joe Biden shows support for Artemis program”, published on 05/02/2021 by Sciencepost. We will then ask you some questions about the text. You can come back to the text to answer the questions.

Back to the Moon: Joe Biden shows support for Artemis program.

Jen Psaki, the White House press secretary, said on Thursday, February 4, that President Joe Biden will continue the Artemis program to land humans on the Moon again. However, the 2024 deadline will likely be pushed back. In 2017, the Trump administration directed NASA to return astronauts to the Moon in 2024 under a program later named Artemis. Unlike the Apollo program, however, it would no longer be a matter of simply “setting foot” on the lunar surface. The objective will be to establish permanent facilities in the South Pole region allowing crews to spend long periods in space. We knew from the start that this 2024 goal was perhaps a bit ambitious. Since his election, Joe Biden had not spoken out about NASA and its plans for lunar exploration. Some even thought his administration might cancel the Artemis program. Finally, White House Press Secretary Jen Psaki reassured everyone on Thursday during her briefing with reporters. “The U.S. government will continue to work with industry and international partners to send astronauts, another man and a first woman, to the moon”, she confirmed. “We support this effort and this endeavor.” On the other hand, Jen Psaki, who was responding to a reporter’s question, did not mention the 2024 deadline set by the Trump administration. Let’s remember that, before sending two astronauts to the Moon, two Artemis missions will be necessary. In 2021, an Orion capsule launched by the Space Launch System (SLS) will make the empty trip around the Moon while Artemis 2, planned for 2023, will make a manned flight around the Moon. An unplanned maneuver has been added to the program for the two astronauts on board to ensure the maneuverability and agility of the capsule during rendezvous operations in orbit. Lunar approaches are never easy, as shown by the number of failed missions in the graph below.

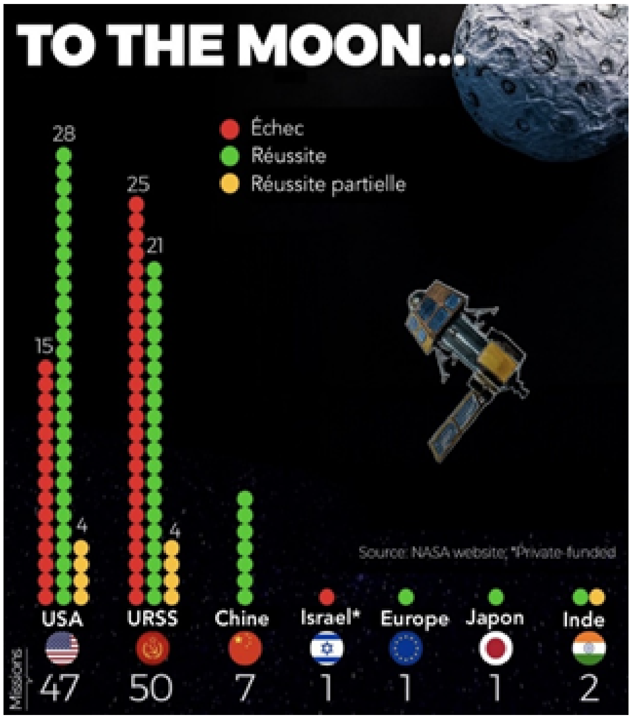

It is the third Artemis mission that will sign the great return of the Americans on the Moon, including the first woman to walk on the lunar soil. The stay on the Moon is expected to last about a week. After Artemis 3, the United States and its international partners will develop a prolonged and strategic presence at the lunar South Pole, with the Artemis Base Camp. Activities at this base camp will set the stage for scientific and economic activities, as well as preparation for the first human mission to Mars in the 2030s. This ambitious program will require a gradual increase in NASA's annual budget, already the highest of any space agency in the world, to over $28 billion by 2023 (see chart below).

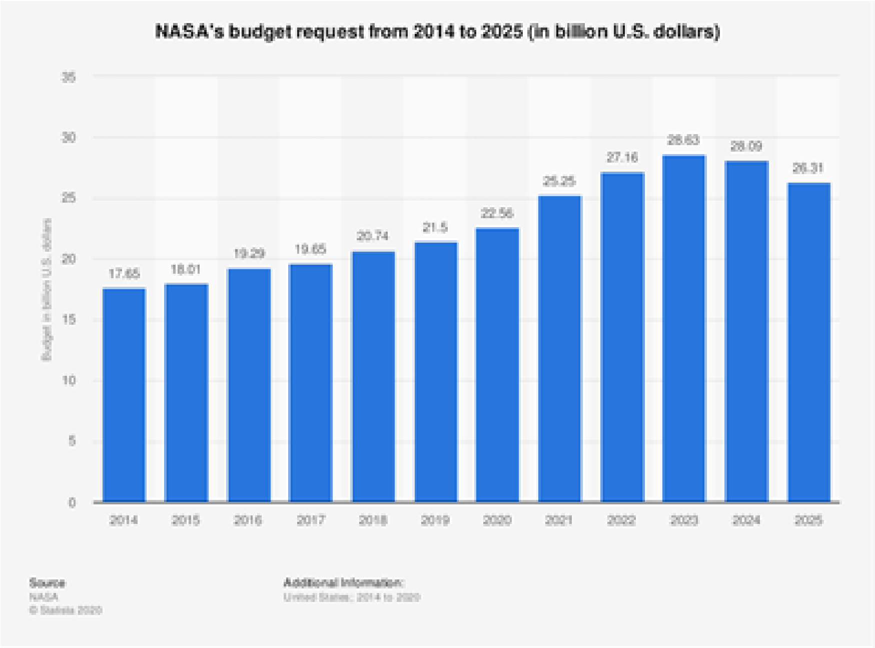

Choose the sentence that best summarizes the text from the suggestions below. Please select only one of the following:

1. The Biden administration has confirmed that American astronauts will return to the moon before 2024, as part of the Artemis program initiated by Donald Trump.

2. The Artemis program, initiated by Donald Trump, has the goal of establishing a permanent base on the moon capable of serving as a foothold for longer and more distant space missions.

3. The Artemis program, initiated by Donald Trump, aims to establish a permanent base on Mars to exploit resources that have become scarce on Earth.

4. The Biden administration has confirmed that American astronauts will return to the moon this year aboard NASA's Orion capsule.

According to the text, in what year was the political decision made to send American astronauts back to the moon? Please select only one of the following:

1. 2017

2. 2024

3. 2021

4. 2023

According to the text, in what year will the first Artemis mission take place? Please select only one of the following:

1. 2021
2. 2022
3. 2023
4. 2024

According to the text and the graph, which country has launched the most missions to the moon to date? Please select only one of the following:

1. China
2. Russia
3. USA
4. Europe

In your opinion, when will a human walk for the first time on the soil of Mars? Please select only one of the following proposals:

1. In 2022
2. In 2023
3. In 2024
4. In 2025
5. Between 2026 and 2030
6. Between 2031 and 2035
7. Between 2036 and 2040
8. Between 2041 and 2049
9. Not before 2050
10. Not at all
11. I don't know

Did you find the article interesting? Please select only one of the following:

1. very interesting
2. interesting
3. not very interesting
4. very uninteresting

Did you learn any new information? Please select only one of the following:

1. I learned a lot (I knew little/nothing about the topic of the text).
2. I learned some new information (I knew a little about the topic of the text).
3. I did not learn anything (I knew well the topic of the text).

On a scale of 1 to 4, where 1 means "I completely disagree" and 4 means "I completely agree", please indicate your opinion about the sentences below.

- Space exploration must be a priority for governments.
- The Moon is a good place to develop new scientific knowledge and test new technologies.
- Given the scarcity of many metals on earth, it is urgent to exploit new deposits on the moon or on Mars.
- It is important to prepare the installation of permanent human colonies in space to ensure the development of human civilization.
- Sooner or later, we will discover elsewhere in space an "other earth" where life is possible.

4 Pre-registration

4.1 Discussion

In this section, we discuss clarification and changes to the pre-registration plan (PAP). In general, we expected both topic treatments to have a negative effect on vulnerable groups. We also hypothesized that the payment threshold would increase stress levels and strengthen the negative effect. However, the positive effect we find is well in line with the literature on goal-based payment schemes.

- **Omission of one main outcome**: The current draft includes the result on cognitive performance (called cognitive ability in the PAP), risk-taking, cognitive reasoning, and the willingness to pay for a training session. We omit the results on over- and underconfidence. Students did well in stating how many matrices they got right, and importantly, there was no treatment effect. Furthermore, overconfidence is the only measure where our two control groups give different results, making the interpretation of the results difficult. Given that this is the only measure out of several where the control treatments differ, we suggest that this is due to noise.
- **Main and secondary outcomes:** We pre-registered "emotions" as a secondary outcome but present it in the paper as a main outcome. It is a verification that the treatment had the intended effect and helps to understand the mechanism. Some other secondary outcomes are mentioned in the text, but most are omitted for brevity.
- **Baseline controls:** We preregistered controlling for "year of study (3 indicators: undergraduate/first 3 years of study, master students, PhD students)". As there were very few PhD students (2.7%), we only include an indicator for undergraduate and group master students with PhD students.

- **Extended controls:** We preregistered to control for "time". We decided to add 4 dummies for morning, afternoon, evening, and night. For the level of fatigue, we added an indicator for "high level of fatigue". For the place of origin and parental place of origin, we just include a dummy for being born in France and their parents being born in France. We omit daltonism as they were very few observations (results are robust to including them). We did not add any further controls.

- **Conditioning on previous performance:** We do not show these results that are only relevant for risk-taking, cognitive reasoning, and willingness to pay for the training session. The results do not change.

- **Pre-registered heterogeneity:** We show all pre-registered heterogeneity for cognitive performance. We only show heterogeneity for the "health students" separately for brevity (there is no significant result for the others). We did not do heterogeneity according to the week of the survey which we said we would only do "in case there are any major events happening around Covid, such as a local lockdown" – which was not the case.

- **Exclusion of observations** The results shown exclude observation as pre-registered. The other two robustness specifications are not shown for brevity but give the same results.

- **Recruitment:** We followed the PAP and "sent an individual invitation to 500 students that registered themselves to participate in the experiment". However, the response rate was slightly lower than expected and we reached the limit of those that signed up before reaching the target sample size. Thus, for the last week of the survey, we invited all those that had previously not received answer (those that were invited but not answered and those that had not been invited yet). Randomization was not affected as it was done on the individual level and neither block-randomized nor stratified.

4.2 Original pre-registration plan

ASPREDICTED

Wharton CREDIBILITY LAB UNIVERSITY of PENNSYLVANIA

CONFIDENTIAL - FOR PEER-REVIEW ONLY

Anxiety, cognitive capacity and economic preferences: An online experiment (#60281)

Created: 03/08/2021 06:36 AM (PT)

This is an anonymized copy (without author names) of the pre-registration. It was created by the author(s) to use during peer-review.
A non-anonymized version (containing author names) should be made available by the authors when the work it supports is made public.

1) Have any data been collected for this study already?

It's complicated. We have already collected some data but explain in Question 8 why readers may consider this a valid pre-registration nevertheless.

2) What's the main question being asked or hypothesis being tested in this study?

We test what is the effect of Covid-related economic and mental-health risk on cognitive ability, confidence in this ability, cognitive reasoning, risk-taking, and the willingness to pay for a real training program. We hypothesize that being confronted with information about economic uncertainty (due to Covid) and information about the social (mental-health) consequences of Covid can lead to a decrease in cognitive ability and cognitive reasoning among a vulnerable part of the student population. This effect is strengthened when these tests are under a more stressful incentive structure. We further test if this information changes risk-taking, confidence (over and under-confidence), and the willingness to pay for a training program.

3) Describe the key dependent variable(s) specifying how they will be measured.

Cognitive ability: Students are asked to answer 10 Raven-matrix-style puzzles that are incentivized and have a time limit. The outcome is the number of correct matrices.

Confidence: Students are asked in an incentivized way to state how many matrices they think they got right (C = belief - truth). We will use i) indicators for strong overconfidence (SOC=1 if C> 1) and strong under-confidence (SUC = 1 if C <-1), and ii) indicators for weak overconfidence (OC=1 if C>= 1) and weak under-confidence (UC = 1 if C <=-1).

Cognitive reasoning: Students are asked 3 incentivized questions for cognitive reasoning (similar to Frederick 2005). The outcome is the number of correct answers.

Risk-taking: Students can invest up to 3€ in this task. 50% chance they get triple the money, 50% they lose their investment. The outcome is the number of Euros they invest (from 0 to 3).

Willingness to pay (WTP): Students are offered to purchase a lottery ticket for a real training program. The price for the lottery ticket is randomly chosen between the options 0,5€, 1€, 2€, 3€. For each choice, students indicate if they would pay that. Their answer represents a binding commitment to buy at that price. WTP will be 1) an indicator of a positive WTP (saying yes to at least one option) 2) their maximum WTP.

4) How many and which conditions will participants be assigned to?

4 treatments:

Economic consequences of Covid: Article and questions about the (negative) consequences of Covid on the economy and youth employment

Social consequences of Covid: Article and questions about the (negative) consequences of Covid on student’s lifestyle and mental health

Control 1: Article and questions about animal welfare

Control 2: Article and questions about space exploration All articles are of approximately the same lengths (similar number of words) and contain 2 graphs. Students are asked to read the articles and answer the same number of comprehension questions, how interesting and novel they found the article, and then the same number of reflective questions priming them to think about their own situation and preferences. Control 1 and control 2 will be pooled if they are not statistically significantly different at 15% level. Otherwise, they will be separately compared to the treatment effects.

For cognitive ability (matrices), we have two payment schedules.

i) Payment per correct matrice

ii) Payment per correct matrice only if they got at least 5 matrices correct (similar to an academic exam).

In the main results for the outcomes that are not the cognitive ability score and there are no differences in the payment schedule, we will pool participants from the two payment schemes. As a secondary analysis, we will check the existence of a persistent effect of the payment scheme.

Participants are randomly assigned to the treatments and payment scheme at the beginning of the survey. All cells are of equal probability.

5) Specify exactly which analyses you will conduct to examine the main question/hypothesis.

We will use a linear model for our analysis.

Baseline controls: gender, year of study (3 indicators: undergraduate/first 3 years of study, master students, PhD students), domain of study (fixed effect for each of 5 domains), baseline cognitive ability (measured through 4 matrices before the treatment), recipient of state scholarship, age

Extended controls: Day of survey, week of survey, time of survey, (other variables where the sample might not be balanced), level of fatigue, whether they are a French native speaker, origin (if they were born abroad + if both their parents were born abroad), color blindness

Conditioning on previous performance: 1) we will add controls for performance in the previous tasks (cognitive reasoning: control for the number of correct matrices, risk-taking: cognitive reasoning and cognitive ability); 2) Condition on the belief about the performance in the cognitive ability tasks. So far, we do not see the necessity to cluster standard errors as treatment is individual.

Since each outcome tests a different hypothesis, we do not think that we need to adjust for multiple hypothesis testing.

We hypothesis important heterogeneity in the effects according to:
Gender, State Scholarship Recipient, Year of study/ being close to graduation (indicator if in the first year and indicator if in the year before graduation), Domain of study, Mental health status (measured through standard mental health questionnaire), Emotional questionnaire administered before or after the treatment (randomized order), Week (in case there are any major events happening around Covid, such as a local lockdown)

6) Describe exactly how outliers will be defined and handled, and your precise rule(s) for excluding observations.
We will exclude respondents that: Take more than 100 minutes to respond to the questionnaire (participants were told that they should finish the questionnaire within 90 minutes in order to be eligible for payment); Take less than 20% of the median time to respond to the questionnaire; Where it is clear from their answers that they are not AMU students: e.g. faculty members, etc; Younger than 18 years and older than 30 years; Individuals who did not complete the survey (except when looking at attrition); PhD Students that have helped us test the survey
Outliers (will be excluded in one specification): Exchange students; PhD Students; Over 27 years old
One specification will also include individuals who finished all the tasks but not the whole questionnaire

7) How many observations will be collected or what will determine sample size? No need to justify decision, but be precise about exactly how the number will be determined.
We plan with a minimum of 1600 students that complete the survey in 5-6 weeks' time. Every week, we sent an individual invitation to 500 students that registered themselves to participate in the experiment. Based on the pilot phase, we expect around 300 complete surveys per week. The minimum number of students might not be attained if response rates go down over time and if not enough students register. The first wave of invitations was sent on Feb 26, 2021. At this point, around 1800 students had registered for the experiment. The sample for each week is randomly selected from those that are registered at that point. Students had to register with their unique university email to avoid duplicates. The minimum number of students is determined by 1) the budget, 2) our impression about how many students would participate in the given time frame, and 3) power calculations based on the pilot study. Power calculations for the cognitive ability test (number of correct matrices) where we have the most treatment cells (8): We compute the smallest change in the mean of the treatment group that can be detected given a total sample of 200 subjects per treatment, assuming equal group allocation, and 80% power using a two-sided 5%-level test. Based on the pilot, we specify a control mean of 7.54 and a standard deviation of 2.11 and 2.98 for the control and treatment, respectively. We find that the minimum detectable size of the effect is -0.72, meaning that our sample size is able to detect medium effects. For 90% power, we find that the minimum detectable size of the effect is -0.83.

8) Anything else you would like to pre-register? (e.g., secondary analyses, variables collected for exploratory purposes, unusual analyses planned?)
Secondary outcomes:
- Emotions: Half of the participants filled a shortened multidimensional mood questionnaire (mdmq) before the treatment and the other half directly afterward. The shortened version of the mdmq contained 12 questions, 4 (2 positive and 2 negative) for each bi-polar scale: good-bad, awake-tired, calm-nervous. For each emotion pair, we will construct a measure (sum of the points scores, negative ones inverted) as outcome variable giving us i) level of feeling good, ii) level of feeling awake, iii) level of feeling calm.
- Attrition (not completing the survey after seeing the treatment)
- Cognitive reasoning awareness: After they answer the cognitive reasoning questions we elicit their perception of the difficulty of the questions by asking what fraction of the sample they expect will get all three questions right. This fraction will be used as a continuous outcome. We will also explore its relation with the number of correct answers and the baseline measure of cognitive ability.
- Time to complete the three cognitive reasoning questions (alternative cognitive reasoning outcome).
- Which training program students prefer: Participants indicate their two preferred topics for the training among 4 modules. We create an indicator for each module which is 1 if the module is selected as their first or second preference.
- Attention (Two attention checks: an Instructional Manipulation Check (IMC); and a Bogus Item question which have an obvious correct answer.)
- Attitude towards vaccination: Indicator if they answer “certainly” or “probably” to being vaccinated
- Which information at the end they find interesting (students can click on topics and then further links are shown, indicator for each topic)
Further exploratory heterogeneity (if testing several heterogeneity measures in the same “family”, then adjusted for multiple hypothesis testing): Locus of control (shorted locus of control measure); Concentration ability (two items, self-judged); academic performance and aspirations; Personal Covid experience; Family background; Self-judgment about their intelligence.
Data collection: Data collection had already started at the point of pre-registration. However, no research team member had access to any of the data (the questionnaires are sent and received securely by our research center’s independent research office, which will hold all data until the end of the last survey wave).

5 Questionnaire (original, in French, version with control treatment (space), emotional questionnaire before the treatment, and threshold payment)

Charger un questionnaire non terminé

Finir plus tard

Etudiants 2021

Vous êtes invité.e à participer à une enquête en ligne, qui vous demandera également de réaliser certaines tâches ou tests (vous pouvez vous munir d'un papier et d'un crayon si vous le souhaitez).

La durée totale de l'enquête est de 45 minutes environ. Bien que l'enquête puisse être complétée sur un smartphone, nous recommandons l'utilisation d'un ordinateur ou d'une tablette. Nous vous demandons de réaliser l'enquête dans un environnement calme, où vous ne serez pas dérangé.e et vous pouvez vous concentrer.

A partir du moment où vous commencez le questionnaire, vous avez maximum 90 minutes (1h30) pour le terminer. Nous vous conseillons donc de commencer le questionnaire uniquement si vous disposez d'au moins 50 minutes devant vous, afin de pouvoir aller jusqu'à la fin de celui-ci. L'enquête doit se dérouler dans un délai de maximum 3 jours après réception du lien de connexion.

Pour vous remercier de votre participation, vous recevrez automatiquement 6€ si vous terminez l'enquête. Au cours du questionnaire, vous pourrez gagner de l'argent supplémentaire mais aussi en perdre ou en dépenser selon vos choix et vos réponses (mais vous repartirez toujours avec minimum 3€). A l'issue de ce questionnaire, votre gain peut aller de 3 à 28€. La somme gagnée vous sera envoyée de manière électronique sous la forme d'une carte cadeau Cultura ou Amazon dématérialisée, d'un ticket de loterie pour gagner un coaching d'insertion professionnelle, ou d'une combinaison de ces formes (au choix).

Si vous dépassez le temps maximum qui vous est imparti pour répondre au questionnaire (90 min), si vous ne terminez pas le questionnaire ou si vous répondez après la fin de la période d'enquête, vous ne serez pas éligible pour recevoir de rémunération.

Merci d'avoir accepté de participer à cette étude. Nous vous demandons de lire attentivement les informations suivantes et de confirmer votre accord en cochant la case ci-dessous.

Objectif de l'enquête

Cette année académique 2020/21 se déroule dans des circonstances sanitaires exceptionnelles. Cette enquête cherche à comprendre précisément l'expérience vécue par les étudiant.e.s universitaires cette année, ainsi que ses répercussions sur leurs capacités d'apprentissage et préférences économiques.

Quelle est la méthodologie et comment se déroule l'expérimentation ?

Vous êtes invité.e à participer à une enquête en ligne, durant laquelle vous serez également amené.e à réaliser certaines tâches ou tests. Pour commencer, il vous sera demandé de répondre à quelques questions courtes sur votre profil. Ensuite, nous vous présenterons une série de tâches testant les capacités de raisonnement et de compréhension de texte, ainsi que l'attitude face au risque et à l'incertitude. Enfin, une série de questions vous seront posées concernant votre expérience durant la crise sanitaire, votre état émotionnel actuel, ainsi que le profil socio-économique de votre famille. Vous recevrez aussi un certain nombre d'informations pouvant vous être utiles. L'ensemble de l'enquête vous prendra environ 45 minutes.

Vos réponses seront collectées de façon anonyme. Les chercheurs ne pourront pas relier vos réponses aux différentes questions à votre identité. En effet, 2 fichiers distincts seront produits, un fichier mentionnant uniquement votre email et le montant de votre rémunération, et un fichier "recherche" anonyme comportant seulement les réponses au questionnaire et aucune information permettant de vous identifier.

Les données à caractère personnel collectées sont destinées exclusivement à la personne chargée de vous rémunérer pour votre participation à cette enquête, elles seront conservées pendant 1 an.

Vos réponses seront utilisées exclusivement à des fins de recherche académique (publications scientifiques et recommandations politiques).

Rémunération

En remerciement pour votre participation, vous obtiendrez une indemnisation financière de minimum 3 euros (pouvant aller jusqu'à 28 euros en fonction de vos réponses), payée par un bon d'achat Amazon ou Cultura, un ticket de loterie pour gagner un coaching d'insertion professionnelle, ou une combinaison de ces formes (au choix).

Pour consulter les conditions d'utilisation des bons d'achat, veuillez cliquer sur l'enseigne qui vous intéresse : Amazon, Cultura

Quels sont vos droits en tant que participant(e) à cette recherche ?

Votre participation est complètement volontaire. Vous pouvez refuser de participer à cette recherche sans avoir à vous justifier. De même, vous pourrez abandonner l'enquête à tout moment sans justification ni conséquence autre que le fait de ne pas recevoir le paiement.

Les investigateurs principaux de cette étude sont Eva Raiber (eva.raiber@univ-amu.fr**) et Timothée Demont (**timothee.demont@univ-amu.fr**), Maîtres de conférences en économie à Aix-Marseille Université et membres du laboratoire Aix-Marseille School of Economics (AMSE).**

Le Comité d'Éthique d'Aix-Marseille Université a étudié ce projet de recherche et a émis un avis favorable à sa réalisation le 03/12/2020. Cette étude est enregistrée au registre tenu par la déléguée à la protection des données du CNRS. Elle est soutenue financièrement par l'Aix-Marseille School of Economics et l'Agence nationale de la recherche scientifique.

* J'ai lu et j'ai compris les informations précédentes et je consens à participer à l'enquête.

Oui | Non

Avant de commencer, merci de répondre à quelques questions de base vous concernant. Les réponses sont volontaires et n'ont pas d'effet sur les étapes et questions suivantes.

Sur quel appareil complétez-vous ce questionnaire?

Veuillez sélectionner une réponse ci-dessous

S'il vous plaît notez que vous n'avez pas encore répondu à cette question. Vous pouvez cependant continuer sans répondre.

- Ordinateur
- Tablette
- Téléphone portable
- Autre

Quelle est votre année de naissance ?

La réponse doit être inférieure ou égale à 2010

Format : yyyy

Quel est votre sexe ?

Veuillez sélectionner une réponse ci-dessous

S'il vous plaît notez que vous n'avez pas encore répondu à cette question. Vous pouvez cependant continuer sans répondre.

Homme

Femme

Non binaire

Ne souhaite pas répondre

Bénéficiez-vous d'une bourse de l'enseignement supérieur sur critères sociaux ?

Veuillez sélectionner une réponse ci-dessous

- [x] Oui
- [ ] Non
- [ ] Ne souhaite pas répondre

Quel est l'échelon de votre bourse ?

Pour vous aider, vous trouverez ci-dessous la grille officielle des échelons.

Types de bourse	Taux annuels sur 10 mois (en euros)	Taux pour les étudiants bénéficiant du maintien de la bourse pendant les grandes vacances universitaires (en euros)
Échelon 0 bis	1 032	1 238
Échelon 1	1 707	2 048
Échelon 2	2 571	3 085
Échelon 3	3 292	3 950
Échelon 4	4 015	4 818
Échelon 5	4 610	5 532
Échelon 6	4 889	5 867
Échelon 7	5 679	6 815

Veuillez sélectionner une réponse ci-dessous

S'il vous plaît notez que vous n'avez pas encore répondu à cette question. Vous pouvez cependant continuer sans répondre.

0bis

1

2

3

4

5

6

○ 7

○ Je ne sais pas

En quelle année d'étude êtes-vous ?

Veuillez sélectionner une réponse ci-dessous

Veuillez choisir ...

Quel est votre domaine d'étude ?

Veuillez sélectionner une réponse ci-dessous

Veuillez choisir ...

Quelle est votre faculté (composante) ?

Veuillez sélectionner une réponse ci-dessous

Veuillez choisir ...

Quel est votre niveau de fatigue actuel ?

Veuillez sélectionner une réponse ci-dessous

S'il vous plaît notez que vous n'avez pas encore répondu à cette question. Vous pouvez cependant continuer sans répondre.

○ Extrêmement fatigué.e

○ Très fatigué.e

○ Un peu fatigué.e

○ Normal

○ En forme / reposé.e

○ Très en forme / très reposé.e

Dans les sections suivantes, vous pouvez gagner de l'argent en fonction du nombre de bonnes réponses que vous obtenez. Réfléchissez donc bien !

Nous allons commencer avec un test d'habilité non-verbale. Vous allez voir plusieurs suites d'images composées de 3 lignes et 3 colonnes représentant 8 objets, le dernier objet étant manquant. Voici un exemple d'image:

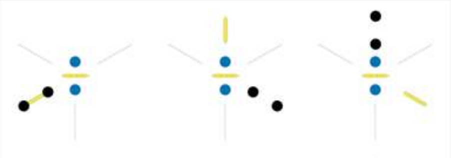

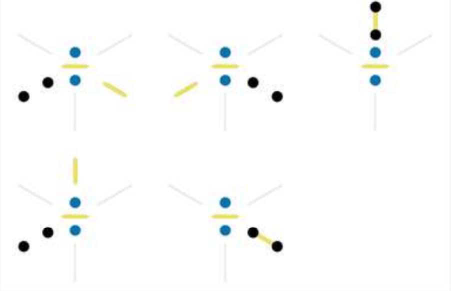

Vous devrez sélectionner l'objet manquant parmi 6 options différentes en cliquant dessus.

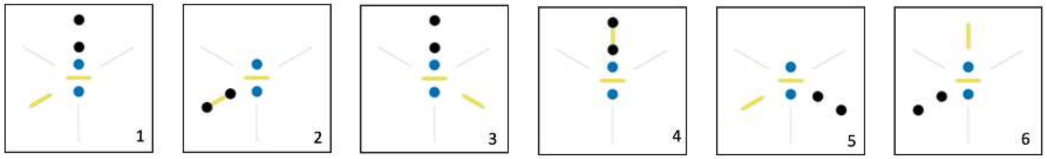

Ici, la bonne réponse était l'option 1. Prenez un instant pour vérifier si vous comprenez pourquoi.

Avant de commencer, vous pourrez vous entrainer sur une nouvelle image. Ensuite, vous verrez 4 autres images pour lesquelles il vous faudra, à chaque fois, trouver la bonne solution.

Vous recevrez 50 centimes par bonne réponse. En fonction de vos réponses, vous pouvez donc gagner jusqu'à 2€ dans cette section (vous aurez d'autres possibilités de gagner de l'argent dans la suite du questionnaire). Attention, votre temps de réflexion est limité à 45 secondes par image. Vous avez 3 minutes au total pour résoudre les 4 images.

A la page suivante, vous verrez l'exemple pour vous entrainer (pour lequel il n'y a aucun paiement). Vous avez 45 secondes pour le résoudre.

Maintenant, vous pouvez vous entrainer avec cet exemple. Attention vous n'avez que 45 secondes.

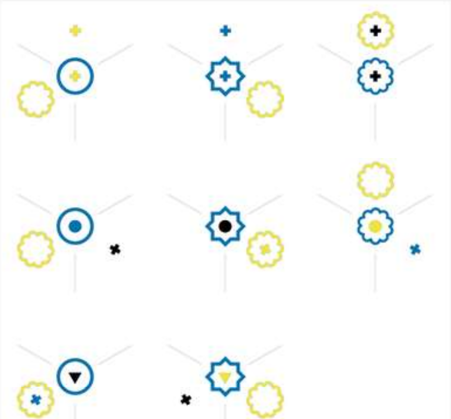

Veuillez sélectionner une réponse ci-dessous

Temps restant 00:00:35

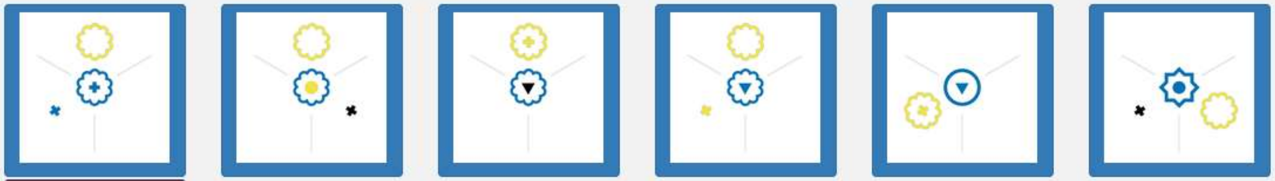

Sans réponse

A la page suivante, vous verrez 4 questions similaires à celle que vous venez de résoudre. Vous avez 3 minutes au total pour répondre à un maximum de questions. Vous recevrez 50 centimes pour chaque bonne réponse. Vous trouverez ci-dessous un tableau de vos gains potentiels en fonction du nombre de bonnes réponses.

Bonnes réponses	Paiement (€)
0	0
1	0,5
2	1
3	1,5
4	2

A vous de jouer !

Sur cette page, vous trouvez 4 suites d'images à compléter en un maximum de 3 minutes.

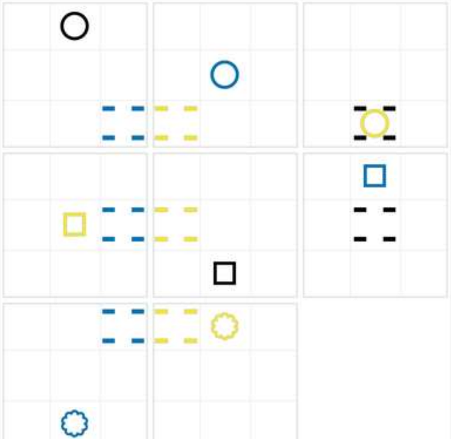

Veuillez sélectionner une réponse ci-dessous

Temps restant 00:01:06

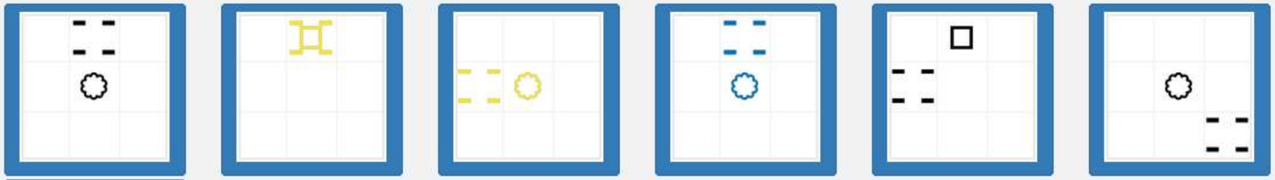

Sans réponse

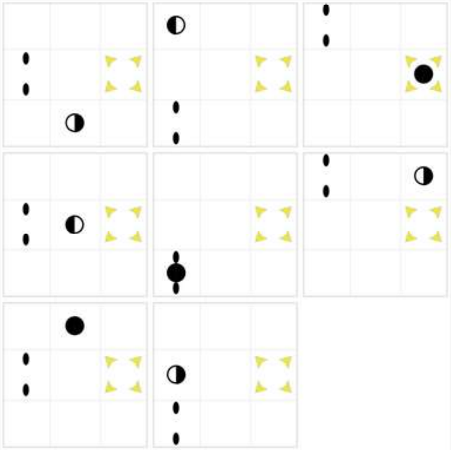

Veuillez sélectionner une réponse ci-dessous

Temps restant 00:01:06

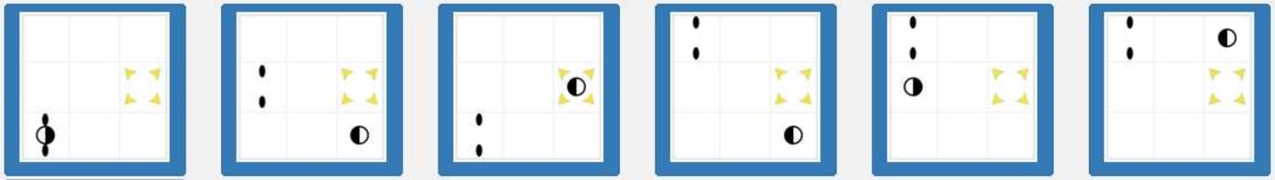

Sans réponse

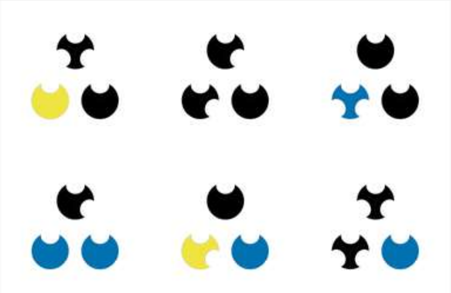

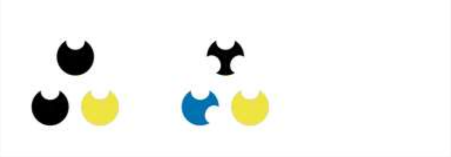

Veuillez sélectionner une réponse ci-dessous

Temps restant 00:01:06

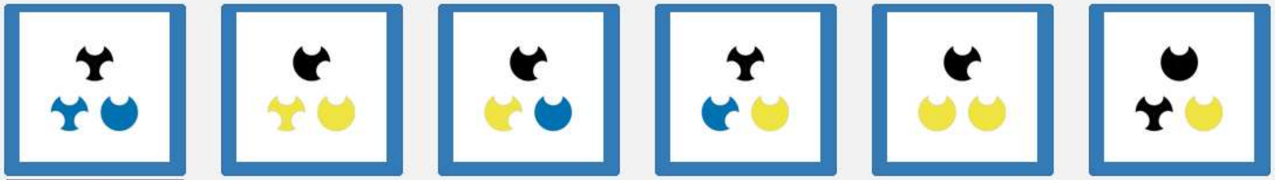

Sans réponse

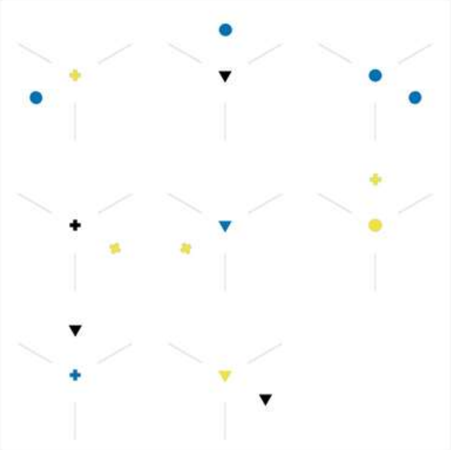

Veuillez sélectionner une réponse ci-dessous

Temps restant 00:01:06

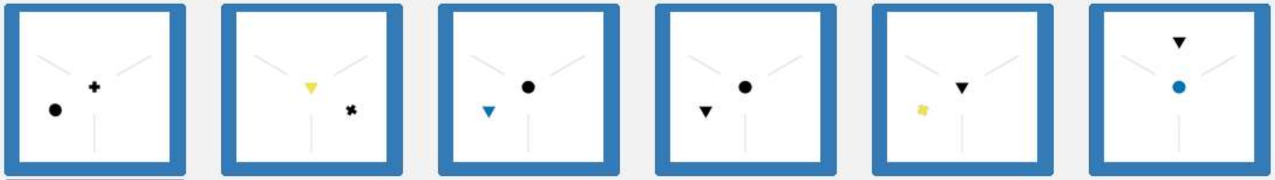

Sans réponse

Félicitations. Nous vous donnerons vos résultats à la fin de l'enquête.

* Vous trouverez ci-dessous une liste d'expressions qui caractérisent différentes humeurs. S'il vous plaît, lisez attentivement la liste, mot par mot, et marquez pour chaque mot la réponse qui représente le mieux l'intensité réelle de votre état d'humeur actuel.

Dans la liste, certains mots décrivent peut-être des humeurs identiques ou similaires. Veuillez ne pas vous en irriter et juger chaque mot indépendamment de votre réponse à un autre mot.

Répondez uniquement en fonction de ce que vous ressentez en ce moment (et non pas ce que vous ressentez normalement ou parfois).

Veuillez indiquer une réponse pour tous les mots. Si vous avez des difficultés à trouver une réponse, indiquez celle qui se rapproche le plus de ce que vous ressentez.

En ce moment, je me sens...

Cette question est obligatoire
Veuillez compléter toutes les parties.

	Certainement pas	Pas	Pas vraiment	Un peu	Très	Extrêmement	Ne souhaite pas répondre
bien	○	○	○	○	○	○	○
en forme	○	○	○	○	○	○	○
stressé.e	○	○	○	○	○	○	○
en colère	○	○	○	○	○	○	○
épuisé.e	○	○	○	○	○	○	○
calme	○	○	○	○	○	○	○
fatigué.e	○	○	○	○	○	○	○
heureux.se	○	○	○	○	○	○	○
inquiet.e	○	○	○	○	○	○	○
plein.e d'énergie	○	○	○	○	○	○	○
triste	○	○	○	○	○	○	○
détendu.e	○	○	○	○	○	○	○

Voici à présent un article d'actualité qui peut vous intéresser. Il s'agit d'un extrait de l'article « Retour sur la Lune : Joe Biden affiche son soutien au programme Artemis », publié le 05/02/2021 par Sciencepost.

Nous vous demandons de le lire attentivement. Nous vous poserons ensuite quelques questions à propos du texte. Vous pourrez revenir au texte pour répondre aux questions.

> # Retour sur la Lune : Joe Biden affiche son soutien au programme Artemis
>
> **Jen Psaki, la secrétaire de presse de la Maison Blanche, a déclaré ce jeudi 4 février que le président Joe Biden poursuivrait le pro- [illegible]**

gramme Artemis visant à faire atterrir à nouveau des humains sur la Lune. En revanche, l'échéance 2024 sera probablement repoussée.

En 2017, l'administration Trump demandait à la NASA de renvoyer des astronautes sur la Lune en 2024 dans le cadre d'un programme nommé ensuite Artemis. À la différence du programme Apollo, en revanche, il ne s'agirait plus de simplement "poser les pieds" sur la surface lunaire. L'objectif sera en effet à terme d'établir des installations permanentes dans la région du pôle sud permettant à des équipages d'effectuer des séjours longue durée dans l'espace.

Nous savions dès le départ que cet objectif de 2024 était peut-être un peu ambitieux. Depuis son élection, Joe Biden ne s'était pas exprimé à propos de la NASA et de ses projets d'exploration lunaire. Certains pensaient même que son administration pouvait annuler le programme Artemis.

Finalement, l'attachée de presse de la Maison Blanche, Jen Psaki, a tenu à rassurer tout le monde ce jeudi lors de son briefing avec les journalistes. "*Le gouvernement des États-Unis continuera de travailler avec l'industrie et ses partenaires internationaux pour envoyer des astronautes, un autre homme et une première femme, sur la Lune*", a-t-elle confirmé. "*Nous soutenons cet effort et cette entreprise*". En revanche, Jen Psaki, qui répondait à la question d'un journaliste, n'a pas mentionné l'échéance 2024 fixée par l'administration Trump.

Rappelons que, avant d'envoyer deux astronautes sur la Lune, deux missions Artemis seront nécessaires. En 2021, une capsule Orion lancée par le Space Launch System (SLS) fera le voyage à vide autour de la Lune tandis qu'Artemis 2, prévue en 2023, réalisera un vol habité autour de la Lune. Une manœuvre non prévue initialement a été ajoutée au programme des deux astronautes à bord pour s'assurer de la maniabilité et de l'agilité de la capsule lors des opérations de rendez-vous en orbite. Les approches lunaires ne sont en effet jamais faciles, comme le montre le nombre de missions ratées sur le graphique ci-dessous.

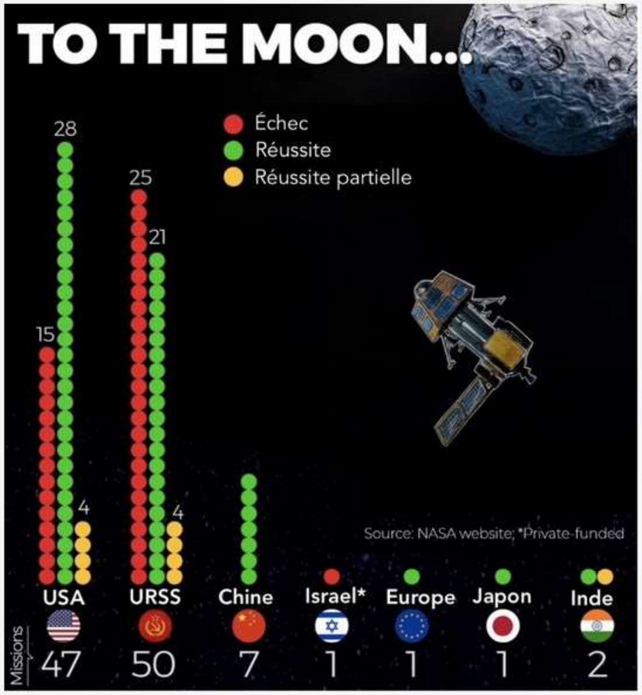

C'est la troisième mission Artemis qui signera le grand retour des Américains sur la Lune, dont la première femme à fouler le sol lunaire. Le séjour sur le sol lunaire est prévu pour durer environ une semaine. Après Artemis 3, les États-Unis et leurs partenaires internationaux développeront une présence prolongée et stratégique au pôle Sud lunaire, avec le Camp de base Artemis. Les activités qui se dérouleront sur ce camp de base ouvriront la voie à des activités scientifiques et économiques, ainsi qu'à la préparation de la première mission humaine sur Mars dans les années 2030.

Cet ambitieux programme nécessitera une augmentation graduelle du budget annuel de la NASA, déjà le plus élevé de toutes les agences spatiales mondiales, qui devrait dépasser les 28 milliards de dollars en 2023 (voir graphique ci-dessous).

NASA's budget request from 2014 to 2025 (in billion U.S. dollars)

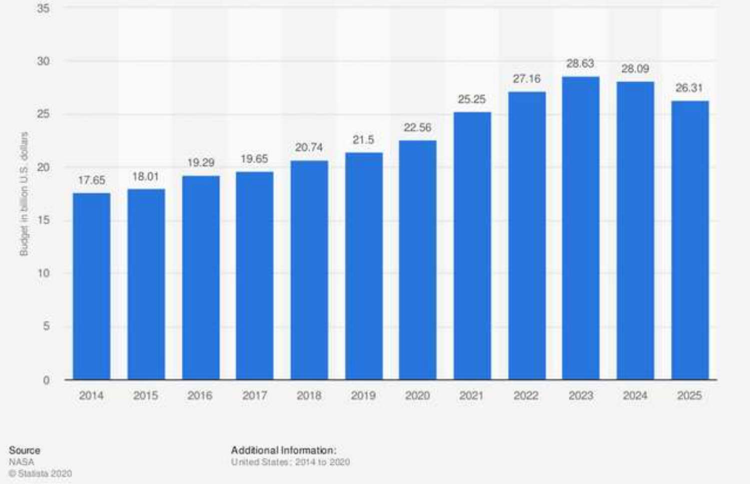

* Choisissez parmi les propositions ci-dessous la phrase qui résume le mieux le texte :

Veuillez sélectionner une réponse ci-dessous

Cette question est obligatoire

- L'administration Biden a confirmé que des astronautes américains retourneront sur la lune avant 2024, dans le cadre du programme Artemis initié par Donald Trump.
- Le programme Artemis, initié par Donald Trump, a comme objectif d'installer une base permanente sur la lune capable de servir de point d'appui à des missions spatiales plus longues et plus lointaines.
- Le programme Artemis, initié par Donald Trump, a comme objectif d'installer une base permanente sur mars pour y exploiter des ressources devenues rares sur la terre.
- L'administration Biden a confirmé que des astronautes américains retourneront sur la lune cette année à bord de la capsule Orion de la NASA.

* Selon le texte, en quelle année la décision politique de renvoyer des astronautes américains sur la lune a-t-elle été prise ?

Veuillez sélectionner une réponse ci-dessous

Cette question est obligatoire

- 2017
- 2024
- 2021
- 2023

* Selon le texte, en quelle année la première mission Artemis se déroulera-t-elle ?

Veuillez sélectionner une réponse ci-dessous

Cette question est obligatoire

- 2021
- 2022

- 2023
- 2024

* Selon le texte et le graphique, quel est le pays qui a lancé le plus grand nombre de missions vers la lune à ce jour ?

Veuillez sélectionner une réponse ci-dessous

Cette question est obligatoire

- Chine
- URSS
- USA
- Europe

* Selon vous, quand un humain marchera-t-il pour la première fois sur le sol de mars ?

Veuillez sélectionner une réponse ci-dessous

Cette question est obligatoire

- En 2022
- En 2023
- En 2024
- En 2025
- Entre 2026 et 2030
- Entre 2031 et 2035
- Entre 2036 et 2040
- Entre 2041 et 2049
- Pas avant 2050
- Jamais
- Je ne sais pas

Avez-vous trouvé l'article intéressant ?

Veuillez sélectionner une réponse ci-dessous

S'il vous plaît notez que vous n'avez pas encore répondu à cette question. Vous pouvez cependant continuer sans répondre.

- très intéressant
- intéressant
- peu intéressant
- très peu intéressant

Avez-vous appris de nouvelles informations ?

Veuillez sélectionner une réponse ci-dessous

S'il vous plaît notez que vous n'avez pas encore répondu à cette question. Vous pouvez cependant continuer sans répondre.

- j'ai beaucoup appris
- j'ai appris quelques nouvelles informations
- je n'ai rien appris

* *Sur une échelle de 1 à 4, où 1 signifie "Je suis en désaccord complet" et 4 signifie "Je suis entièrement d'accord", veuillez indiquer votre opinion pour la phrase ci-dessous.*

Cette question est obligatoire

Veuillez compléter toutes les parties.

	1 - Je suis en désaccord complet	2 - Je suis plutôt en désaccord	3 - Je suis plutôt d'accord	4 - Je suis entièrement d'accord	Ne souhaite pas répondre
L'exploration spatiale doit être une priorité pour les gouvernements.	○	○	○	○	○
La Lune est un bon endroit pour développer de nouvelles connaissances scientifiques et tester de nouvelles technologies.	○	○	○	○	○
Etant donné la raréfaction de nombreux métaux sur la terre, il est urgent d'exploiter de nouveaux gisements sur la lune ou sur mars.	○	○	○	○	○
Il est important de préparer l'installation de colonies humaines pérennes dans l'espace pour assurer le développement de la civilisation humaine.	○	○	○	○	○
Tôt ou tard, nous découvrirons ailleurs dans l'espace une « autre terre » où la vie est possible.	○	○	○	○	○

A la page suivante, vous allez voir des suites d'images similaires à celles que vous avez résolues précédemment.

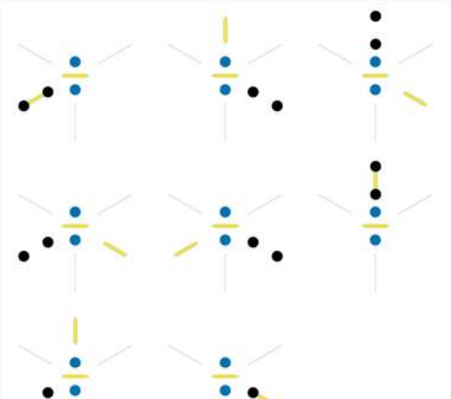

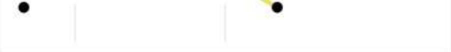

Cette fois, il vous est demandé de trouver l'image manquante pour **10 suites**.

Vous avez **40 secondes par image**, soit 6 minutes et 40 secondes au total, et recevrez **1€ par bonne réponse**. Vous trouverez ci-dessous un tableau de vos gains potentiels en fonction du nombre de bonnes réponses. Vos réponses vous permettrons de gagner jusqu'à 10€ dans cette section.

Bonnes réponses	Paiement (€)
0	0
1	1
2	2
3	3
4	4
5	5
6	6
7	7
8	8
9	9
10	10

A vous de jouer !

Sur cette page, vous trouvez 10 suites d'images à compléter en maximum 6 minutes et 40 secondes.

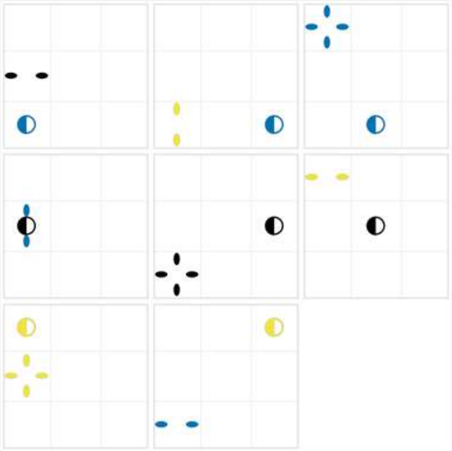

Veuillez sélectionner une réponse ci-dessous

Temps restant 00:04:46

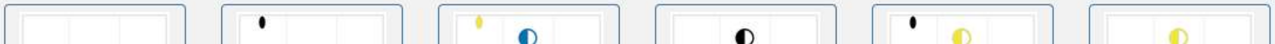

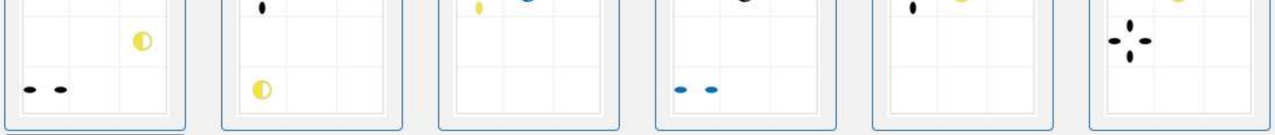

Sans réponse

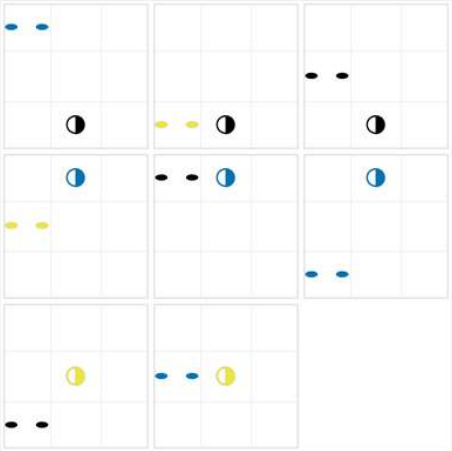

Veuillez sélectionner une réponse ci-dessous

Temps restant 00:04:46

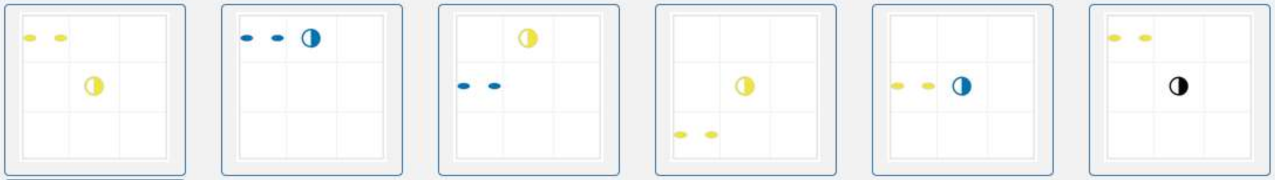

Sans réponse

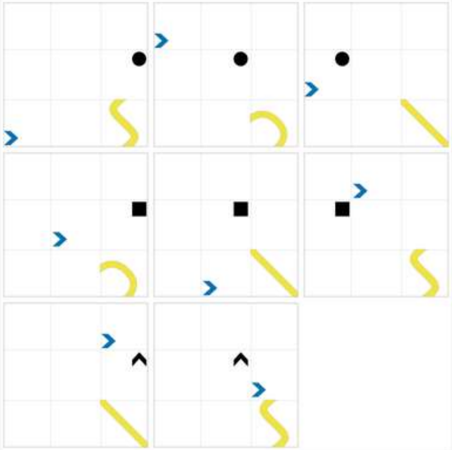

Veuillez sélectionner une réponse ci-dessous

Temps restant 00:04:46

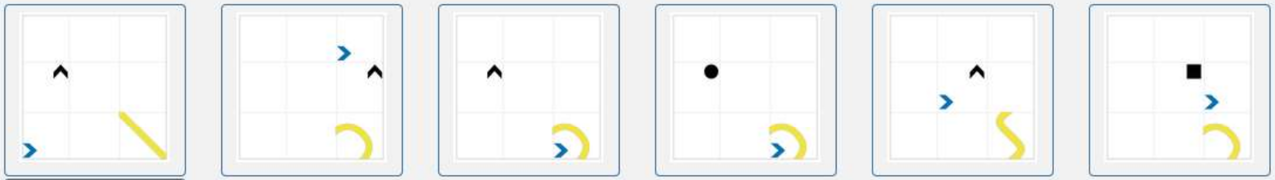

Sans réponse

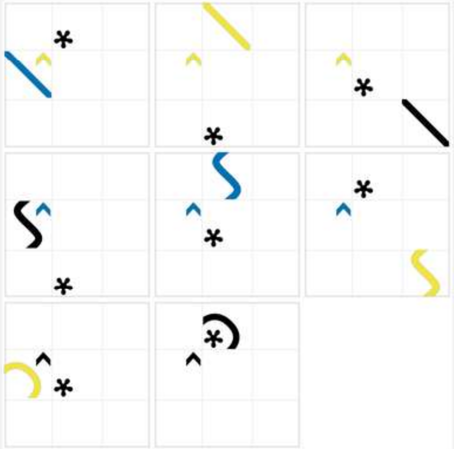

Veuillez sélectionner une réponse ci-dessous

Temps restant 00:04:46

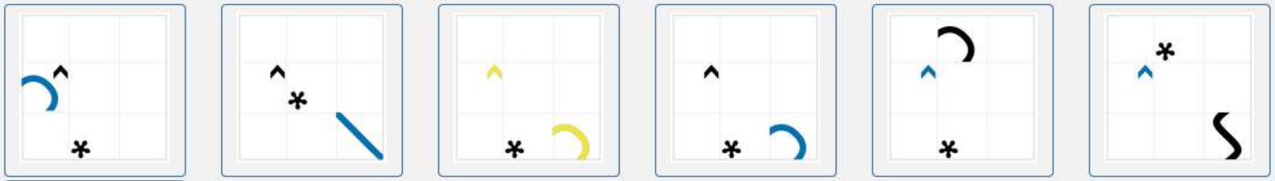

Sans réponse

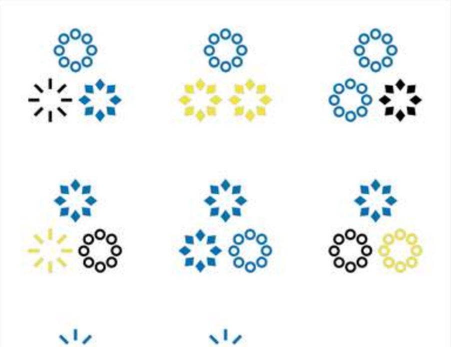

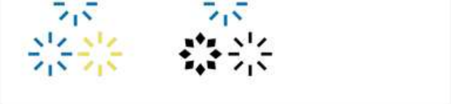

Veuillez sélectionner une réponse ci-dessous

Temps restant 00:04:46

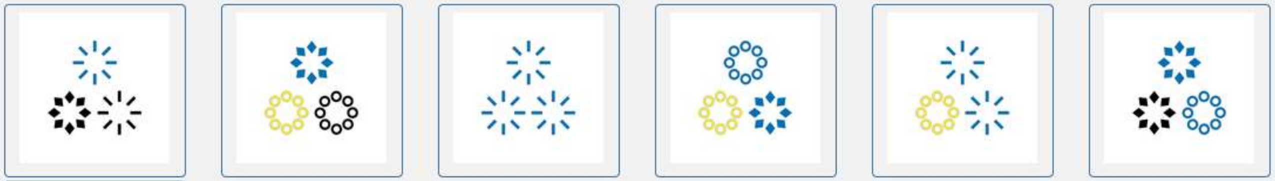

Sans réponse

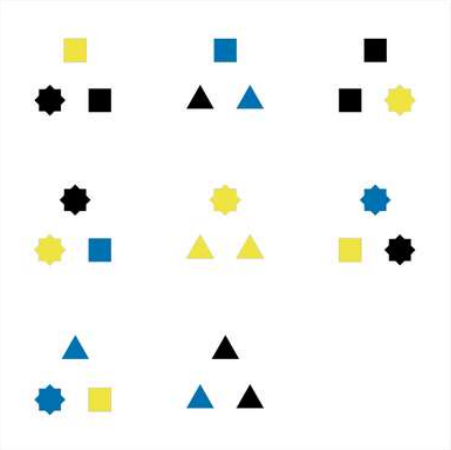

Veuillez sélectionner une réponse ci-dessous

Temps restant 00:04:46

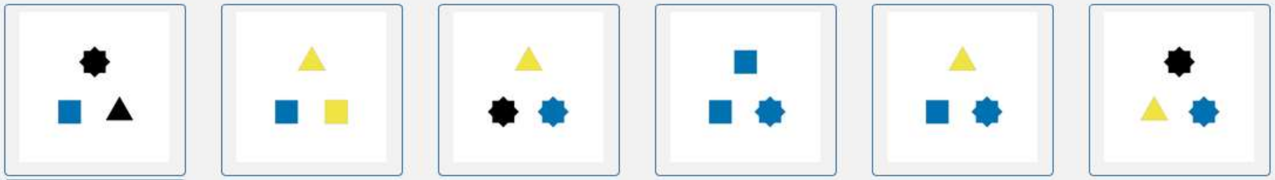

Sans réponse

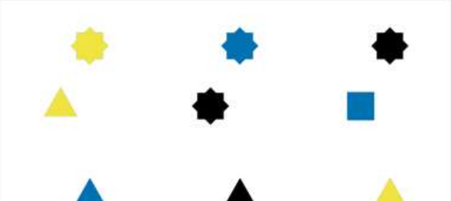

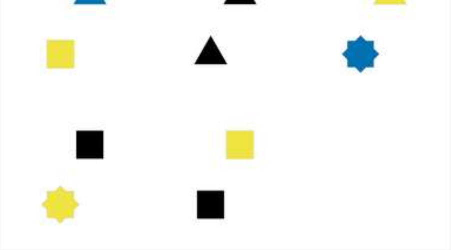

Veuillez sélectionner une réponse ci-dessous

Temps restant 00:04:46

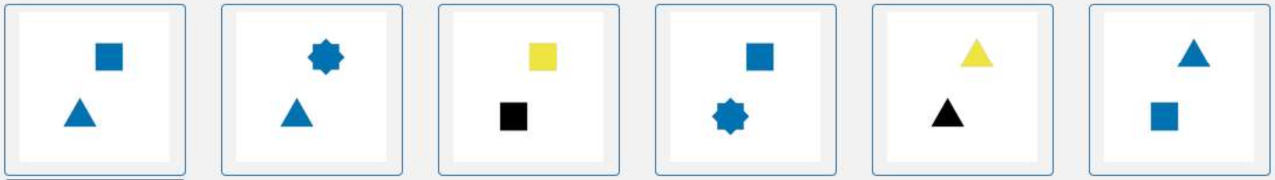

Sans réponse

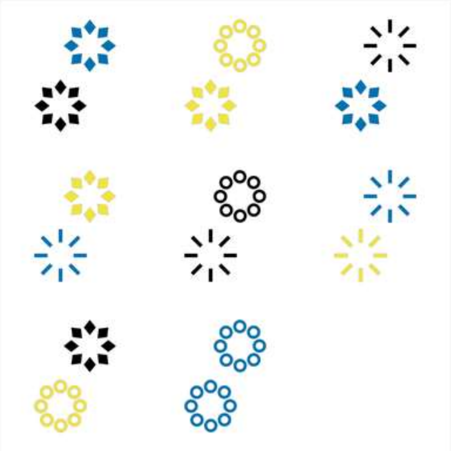

Veuillez sélectionner une réponse ci-dessous

Temps restant 00:04:47

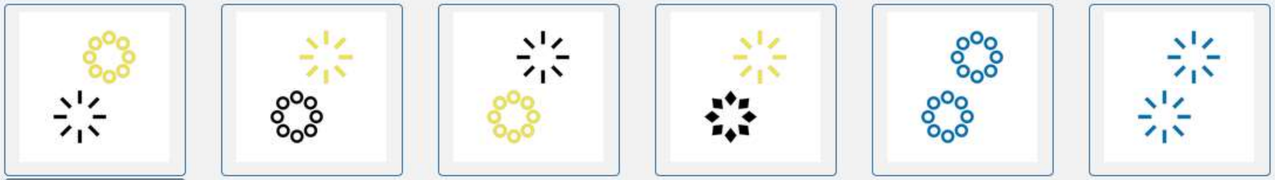

Sans réponse

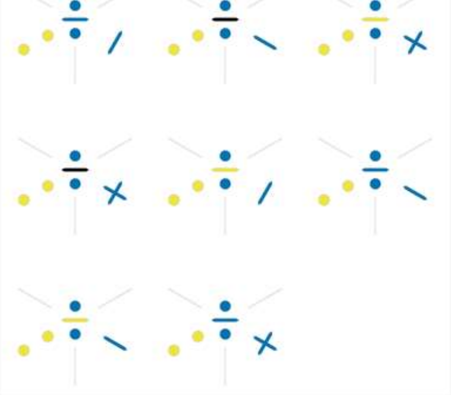

Veuillez sélectionner une réponse ci-dessous

Temps restant 00:04:47

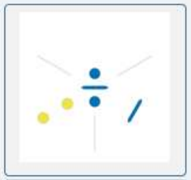 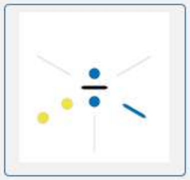 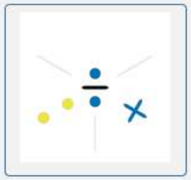 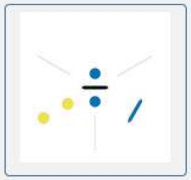 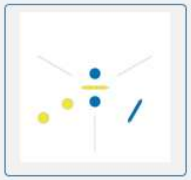 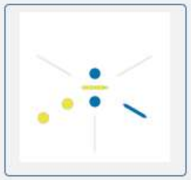

Sans réponse

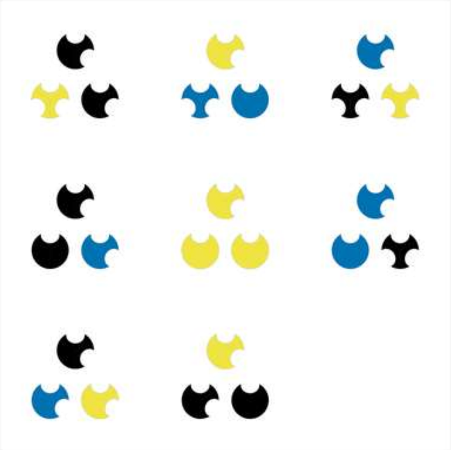

Veuillez sélectionner une réponse ci-dessous

Temps restant 00:04:47

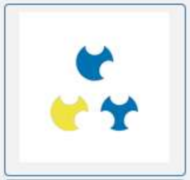 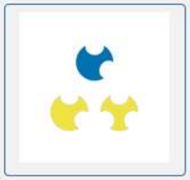 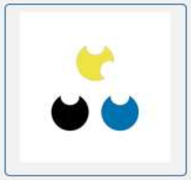 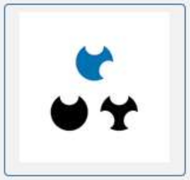 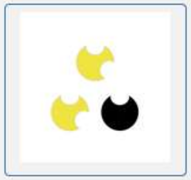 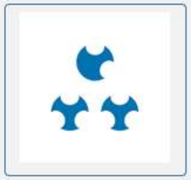

Sans réponse

* Combien de bonnes réponses pensez-vous avoir obtenu au test précédent?

Si vous estimez correctement le nombre exact de vos bonnes réponses, vous recevrez 1€. Si vous vous trompez d'une réponse (+1/-1), vous recevrez 50 centimes. Si vous vous trompez de davantage, vous ne recevrez pas d'argent pour cette question.

Veuillez sélectionner une réponse ci-dessous

Cette question est obligatoire

- 0
- 1
- 2
- 3
- 4
- 5
- 6
- 7
- 8
- 9
- 10

A la page suivante, vous allez voir trois problèmes de différents niveaux de difficultés. Essayez de répondre au maximum d'entre eux. Vous recevrez 1 euro pour chaque réponse correcte. Vous avez maximum 1 minute et demi par problème.

Voici trois problèmes. Vous avez maximum 4 minutes 30 pour répondre aux trois problèmes. Vous pouvez gagner jusqu'à 3 euros dans cette section.

A la boulangerie, vous achetez un pain et un croissant, que vous payez 6 € au total. Sachant que le pain coûte 5 € de plus que le croissant, combien coûte le croissant?

Veuillez sélectionner une réponse ci-dessous

S'il vous plaît notez que vous n'avez pas encore répondu à cette question. Vous pouvez cependant continuer sans répondre.

Temps restant 00:02:37

- 5 centimes
- 10 centimes
- 50 centimes
- 1 euro
- 2 euros
- je ne sais pas

Imaginez que vous inventiez une machine à fabriquer des pizzas. Si deux de ces machines fabriquent deux pizzas en deux minutes, de combien de temps auraient besoin 150 machines pour produire 150 pizzas ?

Veuillez sélectionner une réponse ci-dessous

S'il vous plaît notez que vous n'avez pas encore répondu à cette question. Vous pouvez cependant continuer sans répondre.

Temps restant 00:02:37

- 1 minute
- 2 minutes
- 5 minutes
- 10 minutes
- 100 minutes
- 150 minutes
- je ne sais pas

Imaginez que vous soyez un biologiste moléculaire dans un laboratoire de recherche, étudiant actuellement une souche de bactéries à croissance rapide qui pourrait s'avérer utile pour guérir des maladies rares. Lorsque vous observez une colonie de ces bactéries au microscope, vous remarquez que la colonie met presque exactement une heure à doubler de taille. Si la colonie de bactéries a rempli toute sa boîte de Petri en 10 heures, combien de temps a-t-il fallu à la colonie pour remplir la moitié de la boîte de Petri ?

Veuillez sélectionner une réponse ci-dessous

S'il vous plaît notez que vous n'avez pas encore répondu à cette question. Vous pouvez cependant continuer sans répondre.

Temps restant 00:02:37

- 2 heures
- 5 heures
- 6 heures
- 8 heures
- 9 heures
- je ne sais pas

Cette question est sans rémunération: Selon vous, quel sera le pourcentage des personnes participant à cette enquête qui répondront correctement aux trois questions précédentes ?

Votre réponse doit être comprise entre 0 et 100

Seul un nombre entier peut être inscrit dans ce champ.

Nous vous offrons maintenant la possibilité de participer à une loterie vous permettant de miser jusqu'à la moitié (3 euros) de l'indemnisation financière reçue au départ de l'enquête.

* La loterie à laquelle vous avez la possibilité de participer vous donne 50% de chances de perdre votre mise et 50% de chances de la multiplier par 3. Vous gardez tout l'argent que vous ne misez pas. Le tableau ci-dessous vous montre quel montant d'argent vous obtiendrez en fonction de votre mise et du résultat du tirage au sort.

Somme dont vous disposez au départ	Montant de votre mise	Si vous perdez à la loterie (50% de chance) : Montant qu'il vous reste après la loterie	Si vous gagnez à la loterie (50% de chances) : Montant disponible après la loterie
3	0	3	3
3	1	2	5
3	2	1	7
3	3	0	9

Combien d'argent voulez-vous miser ?

Veuillez sélectionner une réponse ci-dessous

Cette question est obligatoire

- 0€
- 1€
- 2€
- 3€

Nous avons un programme de coaching personnalisé d'insertion professionnelle à offrir.

Ce programme, d'une valeur de 385€, a été spécialement élaboré pour les participants de cette enquête, en partenariat avec l'entreprise de référence en coaching étudiant Futurness.

Le programme se déroulera entièrement à distance et consistera en :

- 3 tests personnels : orientation, personnalité, motivations
- 1 séance de restitution avec un coach professionnel : interprétation des tests et identification de 3 cibles de métiers qui vous correspondent

- 1 séance de restitution avec un coach professionnel : interprétation des tests et identification de 3 cibles de métiers qui vous correspondent
- 1 heure de coaching individuel portant sur 1 ou 2 thème(s) au choix parmi
  - Confiance en soi & Gestion du stress
  - Méthodologie de travail
  - Soutien psychologique
  - Simulation d'entretien d'embauche

* Merci d'indiquer ci-dessous votre thème préféré (vous pourrez modifier votre choix par la suite si vous gagnez le coaching) :

Veuillez sélectionner une réponse ci-dessous

Cette question est obligatoire

- Confiance en soi & Gestion du stress
- Méthodologie de travail
- Soutien psychologique
- Simulation d'entretien d'embauche
- Je ne sais pas
- Rien ne m'intéresse

* Merci d'indiquer ci-dessous votre second thème préféré (vous pourrez modifier votre choix par la suite si vous gagnez le coaching) :

Veuillez sélectionner une réponse ci-dessous

Cette question est obligatoire

- Confiance en soi & Gestion du stress
- Méthodologie de travail
- Soutien psychologique
- Simulation d'entretien d'embauche
- Je ne sais pas
- Rien ne m'intéresse

Comment gagner le coaching ?

Nous tirerons au sort le bénéficiaire parmi les personnes qui auront manifesté leur intérêt via l'achat d'un « ticket de loterie ». Nous avons 100 tickets de loterie à distribuer au total (c'est-à-dire que vous avez une chance sur 100 de gagner le coaching si vous achetez un ticket). Le tirage au sort sera réalisé à la fin de l'enquête. Si vous avez gagné le programme de coaching, nous vous contacterons sur votre adresse email dans quelques semaines et nous vous indiquerons les modalités pratiques pour en bénéficier.

Le prix d'un ticket de loterie permettant d'accéder au tirage au sort pour gagner le programme de coaching sera déterminé aléatoirement entre 0.5, 1, 2 ou 3 euros (chacun de ces prix ayant la même probabilité d'être choisi). Nous vous demandons d'indiquer ci-dessous si vous désirez acheter un ticket dans chacune des 4 possibilités.

Lorsque vous aurez indiqué vos choix, l'ordinateur sélectionnera aléatoirement le prix final et l'achat sera conclu si vous avez répondu "oui" à la question correspondant à ce prix. Dans ce cas, l'argent sera automatiquement déduit des 6€ d'indemnisation reçue pour votre participation à l'enquête. Si vous avez répondu "non" à la question correspondant au prix déterminé aléatoirement par l'ordinateur, vous ne participerez pas au tirage au sort et aucun argent ne vous sera prélevé.

Par exemple, si vous avez répondu "oui" aux deux premières questions (0.5 et 1 €), "non" à la troisième question (2€) et "non" à la dernière question (3€), et que l'ordinateur détermine aléatoirement que le prix final est de 2€, vous payerez 0€ et ne participerez pas au tirage au sort. Si, par contre, le prix final retenu par l'ordinateur est de 1€, vous payerez 1€ et participerez au tirage au sort.

Votre probabilité de participer au tirage au sort augmente donc avec le nombre de « oui » que vous indiquez. Mais rappelez-vous que vous devez être prêt.e à dépenser le prix correspondant à chaque « oui », puisque chaque prix a une chance sur quatre d'être sélectionné par l'ordinateur.

Pour être sûr.e de toujours participer au tirage au sort, répondez "oui" aux 4 questions (et vous payerez 0.5, 1, 2 ou 3€ en fonction du prix déterminé par l'ordinateur); pour être sûr.e de ne jamais participer au tirage au sort et garder les 6 €, répondez "non" aux 4 questions.

* Voulez-vous acheter un ticket de loterie pour participer au tirage au sort si le prix de ce ticket est de 0.5 € ?

Cette question est obligatoire

Oui	Non

* Voulez-vous acheter un ticket de loterie pour participer au tirage au sort si le prix de ce ticket est de 1€?

Cette question est obligatoire

Oui	Non

* Voulez-vous acheter un ticket de loterie pour participer au tirage au sort si le prix de ce ticket est de 2€?

Cette question est obligatoire

Oui	Non

* Voulez-vous acheter un ticket de loterie pour participer au tirage au sort si le prix de ce ticket est de 3€?

Cette question est obligatoire

Oui	Non

Merci. Nous vous indiquerons à la fin de l'enquête si vous avez acheté un ticket de loterie et à quel prix.

Charger un questionnaire non terminé

Finir plus tard

Ce questionnaire n'est actuellement pas activé. Vous ne pourrez pas sauver vos réponses.

Etudiants 2021

Merci. Nous vous indiquerons à la fin de l'enquête si vous avez acheté un ticket de loterie et à quel prix.

Nous avons maintenant terminé la partie de l'enquête qui vous permettait de gagner de l'argent. A la fin de l'enquête, nous vous dirons combien d'argent vous avez gagné en tout et nous vous indiquerons comment les recevoir.

Les questions suivantes concernent vos opinions par rapport à l'avenir. Vous pouvez prendre le temps de réfléchir à chaque question pour répondre le plus sincèrement possible.

* Quand vous pensez à votre futur...

	1 - Pas du tout important	2 - Pas très important	3 - Assez important	4 - Très important	5 - Indispensable	Ne souhaite pas répondre

Quelle est l'importance selon vous de terminer vos études et d'obtenir un diplôme universitaire ?						
Quelle est l'importance **pour vos parents** de terminer vos études et d'obtenir un diplôme universitaire ?						
Quelle est l'importance selon vous d'obtenir une mention (plus de 12/20 de moyenne) en fin d'année ?						
Quelle est l'importance **pour vos parents** que vous obteniez une mention (plus de 12/20 de moyenne) en fin d'année ?						
Quelle est l'importance pour vous d'avoir une "belle carrière" ?						
Quelle est l'importance **pour vos parents** que vous ayez une "belle carrière" ?						
Quelle est l'importance pour vous d'avoir un boulot qui vous plaît vraiment ?						
Quelle est l'importance **pour vos parents** que vous ayez un boulot qui vous plaît vraiment ?						

* Quand vous pensez à votre futur...

	1 - Très peu probable	2 - Peu probable	3 - Assez probable	4 - Très probable	5 - Sûr	Ne souhaite pas répondre
Quelle est la probabilité selon vous que vous réussissiez vos études actuelles ?						
Quelle est la probabilité selon vous que vous ayez une belle carrière ?						
Quelle est la probabilité selon vous que vous ayez un boulot qui vous plaît vraiment ?						

* Quand vous pensez à votre futur...

	Moins de 8	Entre 8 et 10	Entre 10 et 12	Entre 12 et 14	Entre 14 et 16	Plus de 16	Ne souhaite pas répondre
Quelle moyenne générale pensez-vous obtenir lors de vos examens à la fin de ce semestre?							
Quelle moyenne générale pensez-vous obtenir en fin d'année (sur les 2 semestres) ?							

* Dans l'ensemble,

	1 - Je ne suis pas d'accord du tout	2 - je suis plutôt pas d'accord	3 - je suis plutôt d'accord	4 - Je suis entièrement d'accord	Ne souhaite pas répondre
[illegible]					

Je suis optimiste concernant ma vie dans les 5 prochaines années	○	○	○	○	○
Je suis optimiste concernant ma vie dans les 10 prochaines années	○	○	○	○	○

Avez-vous une idée claire du métier ou au moins du secteur d'activité dans lequel vous voudriez travailler ?

Oui | Non

Nous allons maintenant vous poser quelques questions sur votre expérience personnelle de l'épidémie de coronavirus et de cette période.

Où avez-vous passé le premier confinement de mars-avril 2020 ?

Veuillez sélectionner une réponse ci-dessous

- Dans le logement parental
- Dans un logement universitaire (CROUS)
- Logement individuel ou en colocation
- Chez des amis
- A l'étranger
- Autre :

Avec qui avez-vous vécu le premier confinement de mars-avril 2020 ?

Veuillez sélectionner une réponse ci-dessous

- Seul.e
- Avec 1 colocataire
- Avec 2 ou 3 colocataires
- Avec plus de 3 autres colocataires
- En couple
- Avec votre famille
- Avec partenaire et autre(s) colocataire(s)
- Autre :

Avez-vous occupé un emploi rémunéré durant le premier confinement de mars-avril 2020 ?

Oui | Non

Combien de fois vous êtes-vous fait testé pour la Covid-19 depuis le début de la pandémie de la Covid-19 (test nasopharyngé) ?

Veuillez sélectionner une réponse ci-dessous

Veuillez choisir ...

Avez-vous été testé positif à la Covid-19 ?

Oui | Non

Une personne de votre famille proche ou habitant dans votre logement a-t-elle été testée positive à la Covid-19 ?

Oui | Non

Connaissez-vous personnellement une personne (dans votre famille ou votre entourage) qui est décédée de la Covid-19 ?

Oui | Non

Une personne de votre famille proche a-t-elle perdu son emploi depuis le début de la crise de la Covid-19 ?

Oui | Non

* Selon vous, quel est le risque de transmission du virus de la Covid-19...

	Nul ou quasiment nul	Faible	Moyen	Elevé	Ne sais pas	Ne souhaite pas répondre
si vous parlez avec une autre personne à une distance d'1m **sans masque** et **dans un espace extérieur** avec peu d'autres personnes autour	○	○	○	○	○	○
si vous parlez avec une autre personne à une distance d'1m **sans masque** et **dans un espace intérieur** avec peu d'autres personnes autour	○	○	○	○	○	○
si vous parlez avec une autre personne qui n'est pas porteuse du virus de la Covid-19, **sans masque** et **dans un espace intérieur** avec personne autour	○	○	○	○	○	○

Pensez-vous vous faire vacciner contre la Covid-19 dès que possible?

Veuillez sélectionner une réponse ci-dessous

Veuillez sélectionner une réponse ci-dessous

- certainement
- probablement
- je ne sais pas encore
- probablement pas
- certainement pas
- ne souhaite pas répondre

Nous allons maintenant vous poser quelques questions sur vos habitudes.

Combien de jours vous êtes-vous rendu (physiquement) à l'université depuis le début de cette semaine (càd. depuis lundi matin)?

Veuillez sélectionner une réponse ci-dessous

- Aucun
- 1 jour
- 2 jours
- 3 jours
- 4 jours
- 5 jours

Combien de jours vous rendrez-vous (physiquement) à l'université au cours du reste de cette semaine (càd. jusque vendredi soir)?

Veuillez sélectionner une réponse ci-dessous

- Aucun
- 1 jour
- 2 jours
- 3 jours
- 4 jours
- 5 jours

Quelle est la nature de cette semaine?

Veuillez sélectionner une réponse ci-dessous

- Enseignement (normale)
- Vacances
- Révisions
- Stages

- Examens
- Recherche ou travail académique personnel (sans enseignement)
- Autre :

Sur les 5 jours (lundi-vendredi) de la semaine dernière, combien de jours vous êtes-vous rendu (physiquement) à l'université ?

Veuillez sélectionner une réponse ci-dessous

- Aucun
- 1 jour
- 2 jours
- 3 jours
- 4 jours
- 5 jours

Sur les 5 jours (lundi-vendredi) de la semaine dernière, combien d'heures par jour avez-vous consacré à vos études en moyenne (hors cours) ?

Veuillez sélectionner une réponse ci-dessous

- Aucune
- 1 heure par jour
- 2 heures par jour
- 3 heures par jour
- 4 heures par jour
- 5 heures par jour
- 6 heures par jour
- 7 heures par jour
- 8 heures par jour
- 9 heures par jour
- 10 heures par jour

Sur les 5 jours (lundi-vendredi) de la semaine dernière, combien de fois avez-vous rencontré un membre de votre famille en personne (en dehors de la/les personne(s) avec qui vous vivez de manière permanente)?

Veuillez sélectionner une réponse ci-dessous

- Aucune
- 1 fois
- 2 fois
- 3 fois
- 4 fois

- 5 fois
- Plus de 5 fois

Sur les 5 jours (lundi-vendredi) de la semaine dernière, combien de fois avez-vous parlé à un membre de votre famille à distance (téléphone, messagerie, vidéoconférence...) ?

Veuillez sélectionner une réponse ci-dessous

- Aucune
- 1 fois
- 2 ou 3 fois
- 4 ou 5 fois
- 6 à 8 fois
- 8 à 10 fois
- Plus de 10 fois

Sur les 5 jours (lundi-vendredi) de la semaine dernière, combien de fois avez-vous rencontré un.e ami.e proche en personne (en dehors de la/les personne(s) avec qui vous vivez de manière permanente ?

Veuillez sélectionner une réponse ci-dessous

- Aucune
- 1 fois
- 2 fois
- 3 fois
- 4 fois
- 5 fois
- Plus de 5 fois

Sur les 5 jours (lundi-vendredi) de la semaine dernière, combien de fois avez-vous parlé à un.e ami.e proche à distance (téléphone, messagerie, vidéoconférence...) ?

Veuillez sélectionner une réponse ci-dessous

- Aucune
- 1 fois
- 2 ou 3 fois
- 4 ou 5 fois
- 6 à 8 fois
- 8 à 10 fois
- Plus de 10 fois

Sur les 5 jours (lundi-vendredi) de la semaine dernière, combien de jours avez-vous bu de l'alcool ?

Veuillez sélectionner une réponse ci-dessous

- Aucun
- 1 jour
- 2 jours
- 3 jours
- 4 jours
- 5 jours
- Ne souhaite pas répondre

Sur les 5 jours (lundi-vendredi) de la semaine dernière, combien de jours avez-vous consommé de la drogue (dont cannabis) ?

Veuillez sélectionner une réponse ci-dessous

- Aucun
- 1 jour
- 2 jours
- 3 jours
- 4 jours
- 5 jours
- Ne souhaite pas répondre

* Sur les 5 jours (lundi-vendredi) de la semaine dernière, combien de fois...

	**Aucune**	**1 fois**	**2 fois**	**3 fois**	**4 fois**	**5 fois**	**Plus de 5 fois**	**Ne souhaite pas répondre**
Avez-vous fait du sport (à l'intérieur ou à l'extérieur) ?								
Avez-vous participé à une activité associative (en presentiel ou en ligne) ?								

Nous allons maintenant vous poser quelques questions sur vos revenus.

Quelle est votre principale source de revenus personnels ?

Veuillez sélectionner une réponse ci-dessous

- Salaire
- Aide familiale (argent de poche)

Aide familiale (argent de poche)

Bourse

Aide publique (CAF, APL, RSA...)

Autre :

Avez-vous un emploi étudiant rémunéré en ce moment ?

Veuillez sélectionner une réponse ci-dessous

Oui

Non

Ne souhaite pas répondre

Avez-vous un compte bancaire à votre nom spécifiquement dédié à l'épargne ?

Veuillez sélectionner une réponse ci-dessous

Oui

Non

Ne souhaite pas répondre

Vous arrive-t-il d'être à court d'argent à la fin du mois ?

Veuillez sélectionner une réponse ci-dessous

jamais

rarement

souvent

presque tous les mois

ne souhaite pas répondre

Dans un mois normal (sans grosse dépense imprévue), parvenez-vous à mettre de l'argent de côté (épargne) ?

Oui | Non

En ce moment, est-ce que vous épargnez pour un motif particulier ?

Cochez la ou les réponses

je n'épargne pas

aucun motif particulier

pour des dépenses éducatives futures

- [ ] en cas d'urgence
- [ ] pour des dépenses médicales futures
- [ ] pour déménager
- [ ] pour un équipement électronique (ordinateur, tablette...)
- [ ] pour des vêtements ou chaussures
- [ ] pour un véhicule (scooter, voiture...)
- [ ] pour d'autres biens
- [ ] pour des loisirs (vacances, voyages...)
- [ ] pour un évènement social (mariage, anniversaire...)
- [ ] pour améliorer mon logement (travaux, meubles, etc.)
- [ ] pour un achat immobilier
- [ ] pour ma retraite

En cas de besoin financier urgent, pourriez-vous demander de l'aide à quelqu'un ?

Oui | Non

Si vous aviez une dépense imprévue de 100€, pourriez-vous y faire face grâce à votre épargne actuelle ?

Oui | Non

* Au cours des deux dernières semaines, à quelle fréquence avez-vous été dérangé par les problèmes suivants ?

	**Jamais**	**Un jour**	**Plusieurs Jours**	**Plus de la moitié du temps**	**Presque tous les jours**	**Ne souhaite pas ré-pondre**
Eprouver peu d'intérêt ou de plaisir à faire les choses	○	○	○	○	○	○
Vous sentir triste, déprimé.e ou désespéré.e	○	○	○	○	○	○
Bouger ou parler si lentement ("au ralenti") que les autres personnes ont pu le remar-quer	○	○	○	○	○	○
Eprouver d'importantes difficultés à vous en-dormir, à rester endormi.e ou, au contraire, à vous réveiller	○	○	○	○	○	○
Vous sentir très fatigué.e ou avoir peu d'énergie	○	○	○	○	○	○
Avoir peu d'appétit ou trop d'appétit par rap-port à d'habitude	○	○	○	○	○	○
Avoir une mauvaise perception de vous-						

Avoir une mauvaise perception de vous-même, penser que vous êtes un.e perdant.e ou que vous n'avez pas satisfait vos propres attentes ou celles de votre famille						
Avoir des difficultés à vous concentrer sur vos supports de cours ou un ouvrage scientifique ou littéraire						
Avoir la bougeotte et ne pas arriver à tenir en place						
Etre facilement contrarié.e ou irritable						
Eprouver des difficulté à vous détendre						
Eprouver des sensations de peur et avoir l'estomac noué						
Ne plus m'intéresser à mon apparence						
Prendre du plaisir à lire un livre ou regarder un film						
Être si découragé.e que rien ne pouvait vous remonter le moral						

*

	1 - beaucoup plus malheureux.se	2 - un peu plus malheureux.se	3 - ni plus heureux.se ni plus malheureux.se	4 - un peu plus heureux.se	5 - beaucoup plus heureux.se	ne souhaite pas répondre
Par rapport au début de l'année universitaire (septembre 2020), je me sens...						
Par rapport à la période du premier confinement (mars-mai 2020), je me sens...						
Par rapport à la période du deuxième confinement (novembre-décembre 2020), je me sens...						

Voici maintenant quelques questions sur votre perception de vous-même.

* Sur une échelle de 1 à 4, où 1 signifie "Je suis en désaccord complet" et 4 signifie "Je suis entièrement d'accord", veuillez indiquer votre opinion pour chaque phrase ci-dessous.

Dans la liste, certaines phrases décrivent peut-être des sentiments similaires. Veuillez ne pas vous en irriter et juger chaque phrase indépendamment.

Répondez uniquement en fonction de ce que vous ressentez vraiment en ce moment (et non pas ce que vous ressentez parfois ou ce que vous voudriez pouvoir faire dans l'idéal).

	1 - Je suis en désaccord complet	2 - Je suis plutôt en désaccord	3 - Je suis plutôt d'accord	4 - Je suis entièrement d'accord	Ne souhaite pas répondre
Je suis en général prêt à accepter une part de risque si ça me permet de potentiellement obtenir un meilleur résultat					
J'essaie toujours de tirer le meilleur profit de chaque expérience.					

Nous voulons savoir si vous répondez avec attention, pour cela, veuillez sélectionner l'option 2 pour cette phrase quelle que soit votre opinion.	○	○	○	○	○
Je déteste perdre du temps	○	○	○	○	○
Quand quelque chose m'occupe l'esprit, j'ai beaucoup de mal à me concentrer sur autre chose	○	○	○	○	○
J'ai du mal à rester concentré très longtemps sur une même tâche	○	○	○	○	○
Je pense être plus intelligent que la moyenne des personnes de mon âge	○	○	○	○	○
Je regrette souvent d'avoir dépensé de l'argent après coup.	○	○	○	○	○

* Sur une échelle de 1 à 5, où 1 signifie “Je ne suis pas d'accord du tout” et 5 signifie “Je suis entièrement d'accord”, veuillez indiquer votre opinion pour chaque phrase ci-dessous.

Il n'y a pas de bonne ou mauvaise réponse. Répondez uniquement en fonction de ce que vous ressentez vraiment en ce moment (et non pas ce que vous ressentez normalement ou ce que vous croyez être "juste"). Certaines phrases décrivent peut-être des sentiments similaires; veuillez ne pas vous en irriter et juger chaque phrase indépendamment.

	1 - je ne suis pas d'accord du tout	2 - je suis plutôt pas d'accord	3 - ni d'accord, ni pas d'accord	4 - je suis plutôt d'accord	5 - je suis entièrement d'accord	ne souhaite pas répondre
La plupart des évènements malheureux dans la vie sont dûs à la malchance	○	○	○	○	○	○
Je me dis souvent que ce qui doit arriver arrivera d'une manière ou d'un autre	○	○	○	○	○	○
Quand je planifie attentivement mes actions, je suis certain.e de pouvoir les accomplir comme prévu	○	○	○	○	○	○
Les possibilités qu'une personne a dans la vie dépendent des conditions sociales dans lesquelles elle évolue	○	○	○	○	○	○
Je suis convaincu.e d'avoir les capacités nécessaires pour être un jour utile à la société	○	○	○	○	○	○
Pour réussir, il faut travailler dur; le succès n'a rien à voir avec la chance	○	○	○	○	○	○

Pour terminer, nous allons maintenant vous poser quelques questions générales sur votre profil familial.

Votre langue maternelle est-elle le français ?

Oui	Non

Êtes-vous né.e en France ?

Veuillez sélectionner une réponse ci-dessous

- Oui
- Non
- Ne souhaite pas répondre

Dans quel type d'environnement avez-vous passé votre enfance ?

Veuillez sélectionner une réponse ci-dessous

- village (moins de 3000 habitants)
- petite ville (entre 3000 et 15 000 habitants)
- ville moyenne (entre 15 000 et 100 000 habitants)
- grande ville (plus de 100 000 habitants)

Votre père est-il né en France ?

Veuillez sélectionner une réponse ci-dessous

- Oui
- Non
- Je ne sais pas
- Ne souhaite pas répondre

Votre mère est-elle née en France ?

Veuillez sélectionner une réponse ci-dessous

- Oui
- Non
- Je ne sais pas
- Ne souhaite pas répondre

Quelle est la situation professionnelle de votre père ?

Veuillez sélectionner une réponse ci-dessous

Veuillez choisir ...

Quelle est la situation professionnelle de votre mère ?

Veuillez sélectionner une réponse ci-dessous

Veuillez choisir ...

Votre père a-t-il un diplôme universitaire ?

Veuillez sélectionner une réponse ci-dessous

- Oui
- Non
- Je ne sais pas

Votre mère a-t-elle un diplôme universitaire ?

Veuillez sélectionner une réponse ci-dessous

- Oui
- Non
- Je ne sais pas

Le logement de vos parents se situe-t-il en France ? (S'il y en a plusieurs, répondez à propos du logement principal, où vous passez le plus de temps.)

Oui | Non

Quel est le type d'environnement de ce logement parental ?

Veuillez sélectionner une réponse ci-dessous

- village (moins de 3000 habitants)
- petite ville (entre 3000 et 15 000 habitants)
- ville moyenne (entre 15 000 et 100 000 habitants)
- grande ville (plus de 100 000 habitants)

Combien de pièces compte ce logement parental ? (n'incluez que les pièces d'habitation - hors salle de bain, toilette, garage, cave, etc.)

Veuillez sélectionner une réponse ci-dessous

- 1 pièce
- 2 pièces
- 3 pièces
- 4 pièces
- 5 pièces
- 6 pièces
- [illegible]

- 7 pièces
- 8 pièces
- 9 pièces
- 10 pièces

Quelle est la situation matrimoniale de vos parents ?

Veuillez sélectionner une réponse ci-dessous

Veuillez choisir ...

Combien de frères et sœurs avez-vous ?

Seuls des nombres peuvent être entrés dans ce champ.

Habitez-vous chez (un de) vos parents durant la semaine ?

Oui | Non

Avez-vous une connexion internet de qualité raisonnable dans ce lieu ?

Oui | Non

Avez-vous une pièce à vous pour étudier dans le logement parental ?

Oui | Non

Avez-vous un ordinateur personnel vous permettant d'étudier et de suivre les cours à distance ?

Oui | Non

Quelle est votre type de BAC / enseignement secondaire ?

Veuillez sélectionner une réponse ci-dessous

- Général
- Technologique
- Professionnel

Bac étranger

Êtes-vous membre d'une association (sociale, culturelle, sportive...) ou d'un collectif ?

Oui | Non

A quelle date aura lieu votre prochaine évaluation académique (examen sur table, travail à domicile, défense stage ou mémoire, etc.) ?

Veuillez sélectionner une réponse ci-dessous

Je ne sais pas

Autre :

Avez-vous déjà eu vos résultats du premier semestre ?

Oui | Non

Souffrez-vous de daltonisme ou d'achromatopsie ?

Veuillez sélectionner une réponse ci-dessous

Oui

Non

Ne souhaite pas répondre

Félicitations, vous voici à la fin du questionnaire. Merci beaucoup pour vos réponses.

Cette page présente vos gains accumulés au cours des différentes tâches. A la page suivante, nous vous demanderons d'indiquer votre forme de paiement préférée ainsi que votre adresse email qui est indispensable pour recevoir le paiement.

Comment avons-nous calculé vos gains:

Pour vous remercier de votre participation, vous avez reçu 6€ (dont 3€ que vous avez eu la possibilité de rejouer à la loterie).

Vos réponses aux questions:

- Pour les 4 premières images à 0,50c par bonne réponse vous avez gagné (sum(if(M1tf1u30.value == 1, 0.5, 0), if(M1tf1u64.value == 1, 0.5, 0), if(M1tf2u14.value == 1, 0.5, 0), if(M1tf2u52.value == 1, 0.5, 0)))€.

- Pour les 10 images suivantes, vous avez gagné MontantM2€.

- Pour la question vous demandant d'évaluer votre nombre de bonne réponse, vous avez gagné result€.

- Pour les 3 questions de réflexion, vous avez gagné (sum(if(CR1.value == 3, 1, 0), if(CR2.value == 2, 1, 0), if(CR3.value == 5, 1, 0)))€.

La loterie:

Vous avez misé RA.value€ à la loterie (cette somme sera déduite).

Cette loterie vous a permis d'avoir un gain de Proba * Gain * 1.5€.

Ticket coaching :

Le prix du ticket pour participer au tirage au sort du programme de coaching d'insertion professionnelle est de if((PxAlea == 0), 0.5, PxAlea) €.

if((WTP0 == "Y" and PxAlea == 0) or (WTP1 == "Y" and PxAlea == 1) or (WTP2 == "Y" and PxAlea == 2) or (WTP3 == "Y" and PxAlea == 3), "Vous avez acheté un ticket de loterie", "Vous n'avez pas acheté de ticket de loterie")

,

if((WTP0 == "Y" and PxAlea == 0) or (WTP1 == "Y" and PxAlea == 1) or (WTP2 == "Y" and PxAlea == 2) or (WTP3 == "Y" and PxAlea == 3), "nous déduirons donc cette somme de vos gains.", "nous ne déduirons donc rien de vos gains.")

Vos gains s'élèvent donc à
(sum(3, result, Gain, retrait, MontantM2)) + (sum(if(Consent.value == "Y", 3, 0), if(M1tf1u30.value == 1, 0.5, 0), if(M1tf1u64.value == 1, 0.5, 0), if(M1tf2u14.value == 1, 0.5, 0), if(M1tf2u52.value == 1, 0.5, 0), if(CR1.value == 3, 1, 0), if(CR2.value == 2, 1, 0), if(CR3.value == 5, 1, 0)))
€.

Pour valider votre participation et recevoir votre carte cadeau, n'oubliez pas d'indiquer votre adresse e-mail et de cliquer sur ENVOYER à la page suivante.

* Afin de vous envoyer votre carte cadeau dématérialisée, veuillez renseigner votre adresse **email AMU** (nous ne pourrons pas vous envoyer votre carte cadeau à une autre adresse que celle AMU).

(Nous vous rappelons que cette information ne sera pas reliée à vos réponses au questionnaire; votre email servira uniquement à votre rémunération)

* Veuillez saisir à nouveau votre adresse e-mail AMU :

Les deux adresses e-mail saisies sont différentes, merci de les vérifier.

* Pour votre carte cadeau vous avez le choix entre 2 enseignes, laquelle souhaitez-vous :

Veuillez sélectionner une réponse ci-dessous

- Cultura
- Amazon

* Vous recevrez votre carte cadeau dématérialisée d'une valeur de (sum(3, result, Gain, retrait, MontantM2)) + (sum(if(Consent.value == "Y", 3, 0), if(M1tf1u30.value == 1, 0.5, 0), if(M1tf1u64.value == 1, 0.5, 0), if(M1tf2u14.value == 1, 0.5, 0), if(M1tf2u52.value == 1, 0.5, 0), if(CR1.value == 3, 1, 0), if(CR2.value == 2, 1, 0), if(CR3.value == 5, 1, 0))) € à l'adresse : dans un délai de une à deux semaines.

Pour consulter les conditions d'utilisation des bons d'achat, veuillez cliquer sur l'enseigne qui vous intéresse : Amazon, Cultura

* Acceptez-vous d'être recontacté.e par email pour participer à d'autres enquêtes rémunérées ?

Veuillez sélectionner une réponse ci-dessous

- ○ Oui
- ○ Non

Si vous voulez faire une **remarque** ou une **suggestion**, c'est ici :

Pour vous remercier de votre participation, nous voudrions vous suggérer quelques ressources qui pourraient vous être utiles. Quels thèmes vous intéressent ?

Cochez la ou les réponses

- [x] Informations de qualité sur la Covid-19
- [x] Sport, activités, culture
- [x] Services d'aide AMU
- [x] Ressources d'orientation professionnelle
- [ ] Non merci

Informations de qualité sur la Covid-19:

De la BU AMU

De santé publique France

Du ministère de la santé

Du gouvernement

De l'OMS

Du quotidien Le Monde - les décodeurs

Du quotidien Le Monde

De France TV info

Sport, activités, culture:

Visites virtuelles du Louvre

Page "sport à là maison" du SUAPS

Chaîne Youtube "Gym Direct"

Liste des associations étudiantes AMU

Site "Culture chez nous" du Ministère de la Culture (réunissant l'offre en ligne de près de 500 acteurs culturels)

Services d’aide AMU:

Page d'informations AMU sur la Covid-19

Bureau d'aide psychologique universitaire

Service inter universitaire de médecine préventive et de promotion de la santé (SIUMPPS)

Point d'Accueil Ecoute Jeunes (PAEJ)

Espace d'auto-formation aux outils numériques Practicéa

Site de la vie étudiante

Dispositif d'alerte Inst'AMU

Ressources d'orientation professionnelle:

Plan « Un jeune, une solution » du gouvernement

Site universitaire d'insertion et d'orientation (SUIO)

APEC

ONISEP

CEREQ

Etudiants.gouv.fr

Test d'orientation Studyrama

References

Athey, S., & Imbens, G. (2016). Recursive partitioning for heterogeneous causal effects. *Proceedings of the National Academy of Sciences*, *113*(27), 7353–7360.

Athey, S., & Wager, S. (2019). Estimating treatment effects with causal forests: An application. *Observational Studies*, *5*(2), 37–51.

Dinno, A. (2017). tostregress: Linear regression tests for equivalence. stata software package.

Lakens, D. (2017). Equivalence tests: A practical primer for t tests, correlations, and meta-analyses. *Social psychological and personality science*, *8*(4), 355–362.

McKenzie, D. (2012). Beyond baseline and follow-up: The case for more t in experiments. *Journal of development Economics*, *99*(2), 210–221.

Romano, J. P., & Wolf, M. (2005). Stepwise multiple testing as formalized data snooping. *Econometrica*, *73*(4), 1237–1282.

Schuirmann, D. J. (1987). A comparison of the two one-sided tests procedure and the power approach for assessing the equivalence of average bioavailability. *Journal of pharmacokinetics and biopharmaceutics*, *15*, 657–680.

Wager, S., & Athey, S. (2018). Estimation and inference of heterogeneous treatment effects using random forests. *Journal of the American Statistical Association*, *113*(523), 1228–1242.
